# ALS blood expression profiling identifies new biomarkers, patient subgroups, and evidence for neutrophilia and hypoxia

**DOI:** 10.1186/s12967-019-1909-0

**Published:** 2019-05-22

**Authors:** William R. Swindell, Colin P. S. Kruse, Edward O. List, Darlene E. Berryman, John J. Kopchick

**Affiliations:** 1 Heritage College of Osteopathic Medicine, Ohio University, Athens, OH 45701 USA; 2 Department of Internal Medicine, The Jewish Hospital, Cincinnati, OH 45236 USA; 3 Department of Environmental and Plant Biology, Ohio University, Athens, OH 45701 USA; 4 Edison Biotechnology Institute, Ohio University, Athens, OH 45701 USA; 5 The Diabetes Institute, Ohio University, Athens, OH 45701 USA

**Keywords:** Amyotrophic lateral sclerosis, Biomarker, GWAS, Hypoxia, Machine learning, Microarray, Neutrophil, Ribosome, Translation

## Abstract

**Background:**

Amyotrophic lateral sclerosis (ALS) is a debilitating disease with few treatment options. Progress towards new therapies requires validated disease biomarkers, but there is no consensus on which fluid-based measures are most informative.

**Methods:**

This study analyzed microarray data derived from blood samples of patients with ALS (*n* = 396), ALS mimic diseases (*n* = 75), and healthy controls (*n* = 645). Goals were to provide in-depth analysis of differentially expressed genes (DEGs), characterize patient-to-patient heterogeneity, and identify candidate biomarkers.

**Results:**

We identified 752 ALS-increased and 764 ALS-decreased DEGs (FDR < 0.10 with > 10% expression change). Gene expression shifts in ALS blood broadly resembled acute high altitude stress responses. ALS-increased DEGs had high exosome expression, were neutrophil-specific, associated with translation, and overlapped significantly with genes near ALS susceptibility loci (e.g., *IFRD1*, *TBK1*, *CREB5*). ALS-decreased DEGs, in contrast, had low exosome expression, were erythroid lineage-specific, and associated with anemia and blood disorders. Genes encoding neurofilament proteins (*NEFH*, *NEFL*) had poor diagnostic accuracy (50–53%). However, support vector machines distinguished ALS patients from ALS mimics and controls with 87% accuracy (sensitivity: 86%, specificity: 87%). Expression profiles were heterogeneous among patients and we identified two subgroups: (i) patients with higher expression of *IL6R* and myeloid lineage-specific genes and (ii) patients with higher expression of *IL23A* and lymphoid-specific genes. The gene encoding copper chaperone for superoxide dismutase (*CCS*) was most strongly associated with survival (HR = 0.77; P = 1.84e−05) and other survival-associated genes were linked to mitochondrial respiration. We identify a 61 gene signature that significantly improves survival prediction when added to Cox proportional hazard models with baseline clinical data (i.e., age at onset, site of onset and sex). Predicted median survival differed 2-fold between patients with favorable and risk-associated gene expression signatures.

**Conclusions:**

Peripheral blood analysis informs our understanding of ALS disease mechanisms and genetic association signals. Our findings are consistent with low-grade neutrophilia and hypoxia as ALS phenotypes, with heterogeneity among patients partly driven by differences in myeloid and lymphoid cell abundance. Biomarkers identified in this study require further validation but may provide new tools for research and clinical practice.

**Electronic supplementary material:**

The online version of this article (10.1186/s12967-019-1909-0) contains supplementary material, which is available to authorized users.

## Background

Amyotrophic lateral sclerosis (ALS) is a fatal disease with inherited (familial) forms and sporadic subtypes arising spontaneously from gene-environment interactions. Mutations in superoxide dismutase (*SOD1*) were the first to be associated with ALS [1], but in recent decades additional susceptibility genes have been identified (e.g., *TDP43*, *C9orf72*), reflecting a complex genetic basis for most forms of the disease. Ongoing epidemiologic studies have also uncovered environmental risk factors, which appear to include smoking, low body mass index, poor dietary antioxidant intake, vigorous physical activity, head injury, and occupational exposures to heavy metals or pesticides [2]. At present, few ALS treatment options are available, including the glutamate antagonist riluzole (Rilutek/Teglutik) and antioxidant edaravone (Radicava/Radicut), along with dextromethorphan/quinidine (Nuedexta) for pseudobulbar affect. However, despite frequent clinical trial setbacks [3, 4], research towards new ALS treatments has pressed forward, and promising candidate therapies are now at various stages of development and clinical testing (e.g., Methylcobalamin, Mastinab and NP001) [5]. In this setting, the lack of ALS biomarkers has been cited as a factor limiting the identification, development and testing of new drug candidates [6, 7]. Investigators have thus worked to expand the set of available ALS biomarkers, which now includes clinical performance measures, genetic risk factors, and measures derived from biological fluids (CSF, blood and urine) and neurophysiology or neuroimaging studies [8, 9]. Despite this progress, ALS biomarkers selected for use in clinical trials have varied from study-to-study, reflecting the absence of definitive “gold standard” biomarkers widely agreed upon by ALS researchers [8, 9].

Fluid-based ALS biomarkers have been suggested from studies of CSF, blood, urine and saliva, and in principle would offer objective, quantitative, and potentially multi-dimensional tools for investigators [6, 10]. CSF biomarkers have been viewed as the most promising due to direct contact between CSF and central nervous system tissues [11], but a drawback is that CSF sampling requires lumbar puncture, which is time-consuming, cannot be performed in all patients, and may cause adverse effects (e.g., headache). As an alternative, peripheral blood is easily sampled and a promising biomarker source [12]. Although ALS is primarily a disease of motor neurons, the rationale for blood-based biomarkers is supported by factor exchange at the blood-CSF barrier [6], which may be enhanced in ALS patients due to barrier damage and loss of pericytes [13, 14]. Experimental evidence also supports a role for immune cells in disease progression [15–17] with protective and deleterious immune responses in ALS patients [18, 19]. Blood-based biomarkers with clinical utility for ALS appear to include phosphorylated neurofilament heavy chain (pNFH), neurofilament light chain (NFL), microRNAs (e.g., miRNA-1234-3p), inflammatory markers (e.g., IL-6, IL-8, IL-5 and IL-2), TDP-43, and metabolites (e.g., glutamate and lysine) [6]. Serum and plasma NFL levels, for example, were shown to be effective for distinguishing ALS patients from healthy CTL subjects with a sensitivity of 89–90% and specificity of 71–75% [20]. If sufficiently validated, such biomarkers could be used for ALS diagnosis, prognosis of clinical course, prediction of treatment response, and pharmacodynamic monitoring [8]. Blood-based biomarkers can also be used to screen drug responses in humans or mice to identify compounds warranting investigation as new ALS drug candidates [21]. Finally, given that development and validation of ALS mouse models remains a longstanding research challenge [22, 23], blood-based biomarkers could be used to assess whether ALS-like mouse phenotypes have biomarker profiles similar to the human disease [24, 25].

Gene expression profiling has previously been used to comprehensively analyze mRNA abundance to identify neurodegenerative disease biomarkers [26, 27]. Along these lines, prior studies have used microarray or RNA-seq expression profiling of whole blood or peripheral blood mononuclear cells (PBMCs) to compare gene expression in ALS patients and control (CTL) subjects (Additional file 1) [28–32]. This has led to the identification of differentially expressed mRNAs with altered expression between ALS patients and CTL subjects. In these studies, however, sample sizes have been limited (*n* ≤ 43 individuals in ALS and CTL groups), which may be insufficient for a heterogeneous disease such as ALS [33] and increase the risk for type I and II errors and findings with poor repeatability [34]. Recently, however, a large microarray dataset from peripheral blood of ALS patients and controls was generated [31] with sample sizes far exceeding those in prior work (*n* = 1117 participants). These data represent the best resource now available for identifying ALS blood biomarkers, although an initial analysis was challenged by technical issues related to batch effects and the combination of data from two cohorts with expression evaluated using different microarray platforms [31]. Using several classification modeling approaches, expression-based models from this initial study could discriminate between ALS and CTL subjects (0.87 ≤ area under curve (AUC) ≤ 0.90), but were less effective at discriminating ALS patients from those with ALS-mimic diseases (MIM) (0.65 ≤ AUC ≤ 0.68) [31]. It was also concluded that prediction of ALS patient survival using blood gene expression markers was poor [31]. These results raise questions regarding the clinical utility of blood-based ALS biomarkers, although it remains possible that alternative analysis approaches may resolve technical variability in these data to generate new insights.

This study provides an independent analysis of the large-cohort microarray dataset generated by van Rheenen et al. [31]. We apply an alternative data normalization strategy [35] and implement a series of analyses not applied previously. Our results provide new insights into processes and pathways associated with differentially expressed genes (DEGs) [36, 37], expression of DEGs in exosomes [38, 39], overlap between DEGs and genes near ALS GWAS loci [40], shifts in immune cell abundance or activity in ALS patients [41], and patient subgroups based upon expression profile heterogeneity among patients [42, 43]. We utilize multiple machine learning approaches [44] to generate diagnostic models (ALS vs. CTL/MIM), and use Cox proportional hazards (PH) models to generate a multivariate expression signature that predicts ALS patient survival.

## Methods

### Patient cohorts

The study was performed with two cohorts (GSE112676 and GSE112680) and gene expression evaluated using two microarray platforms (Illumina HumanHT-12 V3.0 and HumanHT-12 V4.0 expression beadchip arrays) [31]. The V3.0 platform was used to profile expression in the GSE112676 cohort (*n* = 233 ALS and 508 CTL samples), and the V4.0 platform was used to profile expression in the GSE112680 cohort (*n* = 164 ALS, 137 CTL and 75 MIM samples) (Additional file 2A). Cohort demographics have been described previously [31]. In brief, ALS, CTL and MIM groups each included a higher percentage of males (≥ 55.3%; Additional file 2B) with average ages of 63.6, 62.6 and 57.9, respectively (Additional file 2C). Most patients (> 60%) had spinal- rather than bulbar-onset ALS (Additional file 2D). The GSE112680 cohort had a larger percentage of patients with *C9orf72* repeat expansions (12.8% vs. 5.2%, Additional file 2G). Survival was defined as the time interval between disease onset to death, tracheostomy or noninvasive ventilation [31]. Given this definition, median survival was 2.44 years with 50% of patients surviving 1.59 to 3.87 years (Additional file 2F). The 75 MIM patients had been diagnosed with diverse ALS-like conditions, but the most common diagnoses were benign fasciculations (*n* = 9), spinal muscular atrophy (*n* = 8) and myelopathy (*n* = 8) (Additional file 2G).

### Microarray normalization and integration approach

An original analysis of these data had identified “batch effects” as a complicating factor [31], and we further expected that microarray platform-specific effects would be present [45]. To avoid confounding batch and platform-specific effects, we followed a “late stage” data integration strategy [46], by first analyzing data from each platform individually (V3 and V4) and correcting for batch effects as appropriate. This proved to be preferable to combining V3 and V4 data and attempting to correct batch and platform-specific effects simultaneously. Differential expression analyses were thus performed using data from each platform separately, and differentially expressed genes (DEGs) were identified by integrating summary statistics (fold-change and standard errors) via a random effects meta-analysis model. For other analyses (aside from differential expression testing), we adopted an “early stage” integration approach [46], in which it was necessary to combine expression values from both datasets for corresponding genes. In such cases, batch-corrected log_2_-scaled expression values from each platform were *Z*-score normalized, respectively, to remove any platform-specific effects and maximize comparability of expression values.

### GSE112676 processing and normalization

Gene expression estimates for 741 samples were generated using the Illumina HumanHT-12 V3.0 expression beadchip platform (233 ALS and 508 CTL samples) [31]. Raw expression intensities and detection p-values were downloaded in June 2018 (GSE112676_HT12_V3_preQC_nonnormalized.txt). Detection p-values for 520 samples were negatively correlated with expression intensities as expected (*r*_s_ < − 0.90), but detection p-values for 221 samples were positively correlated with expression intensities (*r*_s_ > 0.90). Given this pattern, it was likely that detection p-values for the latter group did not correspond to actual p-values (P) but rather corresponded to 1 − P. Detection p-values for these 221 samples were thus subtracted from unity and after correction there was a strong negative correlation between detection p-values and expression intensity for all 741 samples as expected (*r*_s_ < − 0.90). The 741 samples varied with regard to median intensity, intensity IQR, and the number of protein-coding genes with detectable expression at a threshold of P < 0.05 (Additional file 3A–C). Overall, 8847 protein-coding genes were detected per sample on average (range: 5035–11,392; Additional file 3C). Sample index plots for these parameters were suggestive of a batch effect, with a higher signal IQR and number of detected genes for the first set of 448 samples (GSM3076582-GSM3077650) as compared to the second set of 293 samples (GSM3077652-GSM3078510) (Additional file 3D–F).

Background correction was performed using the normal–exponential convolution model, in which intensities are assumed to be the sum of two components, including a normally distributed background noise component and an exponentially distributed signal component (R package: limma; function: backgroundCorrect) [47]. Quantile normalization was applied after background correction to normalize expression intensities across the 741 samples (R package: limma; function: normalizeBetweenArrays) [47]. This generated normalized intensity estimates for 48,803 probes, although some of these were unannotated or quantified expression for the same gene (R annotation package: illuminaHumanv3.db). For each human gene, therefore, a single representative probe was selected, yielding a reduced set of 19,236 probes each corresponding to a unique human gene. For genes associated with multiple probes, the single probe with highest average expression across the 741 microarray samples was chosen as a representative. A final filtering step was performed to include 18,035 probes assaying the expression of protein-coding genes, i.e., those genes with an “NM_” and “NP_” prefix in their Reference Sequence (RefSeq) database identifiers.

These preprocessing steps generated a normalized expression matrix for 741 samples and 18,035 probes associated with a non-redundant set of protein-coding genes. The 741 samples were plotted with respect to the first 2 and 3 principal component (PC) axes, which again demonstrated a clear batch effect unrelated to disease status or sex (Additional file 3G, H). Similar to the pattern described above, the first PC score (PC1) differed according to sample index, with one batch corresponding to 448 samples (GSM3076582-GSM3077650) and a second batch corresponding to 293 samples (GSM3077652-GSM3078510) (Additional file 3I). To correct this effect, we first applied surrogate variable analysis (R package: sva; function: sva), as implemented previously for these data [31], with data adjusted using 1 surrogate variable chosen based upon asymptotic conditional singular value decomposition (R package: sva; function: num.sv) [48]. This improved the batch effect in some analyses (Additional file 3J, K), but did not resolve the relationship between PC1 scores and the sample index (Additional file 3L). We therefore applied the ComBat algorithm as an alternative strategy (R package: sva; function: ComBat) [35]. This approach removed any apparent batch effect in PC plots (Additional file 3M, N) and also resolved the relationship between sample index and PC1 score (Additional file 3O).

Following ComBat correction, one outlier sample (GSM3077426) was identified with respect to PCs 1 and 3 (Additional file 3M–O). This sample was removed prior to further analyses, but otherwise no outliers were visually evident from PC plots (Additional file 3M–O). Following removal of GSM3077426, Grubb’s test [49] for univariate outliers was non-significant with respect to each of the first 2 PC axes (P ≥ 0.74; R package: outliers; function: grubbs.test). The removal of 1 sample outlier (GSM3077426) was appropriate in our judgement and may be considered a less aggressive approach to outlier exclusion. For comparison, 67 outlier samples (34 ALS, 33 CTL) were identified and excluded in the initial GSE112676 analysis reported by van Rheenen et al. [31].

### GSE112680 processing and normalization

Gene expression estimates for 376 samples were generated using the Illumina HumanHT-12 V4.0 expression beadchip platform. Raw expression intensities and detection p-values were downloaded in June 2018 (GSE112680_HT12_V4_preQC_nonnormalized.txt). The 376 samples included 164 from ALS patients, 137 from control subjects, and 75 from MIM patients (Additional file 2G). Detection rate p-values were negatively correlated with signal intensity estimates for all samples as expected (*r*_s_ < − 0.99). Samples with a higher GSM identifier index tended to have increased median intensity and signal IQR but a lower number of detected genes (Additional file 4A–F). An average of 9152 protein-coding genes was detected among the 376 samples (range: 6939–11,061). Background correction and quantile normalization was performed as described above (R package: limma; functions: backgroundCorrect and normalizeBetweenArrays) [47]. This yielded normalized expression intensities for 47,323 probes and 376 samples. To limit redundancy in subsequent analyses, multiple probes associated with the same gene were filtered and a single representative probe was selected (R annotation package: illuminaHumanv4.db). This generated a filtered expression matrix consisting of 20,937 probes each representing a unique human gene. These probes were further filtered to include only protein-coding human genes (with “NM_” and “NP_” prefixes in RefSeq identifiers), leaving 18,490 probes upon which further analyses were based.

The 376 samples were plotted with respect to the first 3 PC axes, which suggested a weak batch effect involving only a small number of samples (Additional file 4G, H). Inspection of PC1 scores suggested that this effect was again (as above) related to sample ordering (GSM indices), with higher scores for the last 82 samples (GSM3080099–GSM3080180) as compared to the first 294 (GSM3079737–GSM3080098) (Additional file 4I). We first attempted to remove this effect using surrogate variable analysis (R package: sva; function: sva), with data adjusted using 2 surrogate variables (R package: sva; function: num.sv) [48]. As in the other cohort, this improved the batch effect for some analyses (Additional file 4J, K), but not the association between PC1 score and sample index (Additional file 4L). The alternative correction using ComBat was therefore applied (R package: sva; function: ComBat) [35], which did succeed in removing the batch effect (Additional file 4M, N) and the association between PC1 scores and sample index (Additional file 4O). There was no strong visual evidence for outliers and accordingly Grubb’s test for univariate outliers was non-significant with respect to the first 2 PC axes (P = 1.00) (R package: outliers; function: grubbs.test). The initial analysis of van Rheenen et al. [31] had identified and excluded 20 samples as outliers (13 ALS, 7 CTL) for the GSE112680 dataset, but this did not appear necessary following the normalization, batch correction and filtering procedures applied in the current analysis.

### GSE112676 differential expression analysis

The above processing steps yielded an expression matrix for 18,035 protein-coding genes and 740 samples (232 ALS and 508 CTL samples). Differential expression analyses were performed using a subset of 11,210 genes with detectable expression in at least 20% (> 148/740) of samples. Effects of sex were removed by fitting a linear regression model for each gene, with expression as the response variable and sex (male or female) coded as a 0–1 categorical predictor variable. Residuals from the regression fit were used as sex-corrected expression values in subsequent analyses. Differential expression was evaluated using limma generalized least square linear models with moderated t-statistics (R package: limma; functions: lmFit and eBayes) [50]. To control the false discovery rate (FDR), raw p-values were corrected using the Benjamini–Hochberg method [51]. In all analyses, we define DEGs as genes with at least 10% expression change (FC > 1.10 or FC < 0.909) and FDR less than 0.10. Given these thresholds, we identified 2381 ALS-increased DEGs and 2646 ALS-decreased DEGs using only GSE112676 samples (Additional file 5). Up- and down-regulated FC estimates were symmetrically distributed and not associated with mRNA abundance (see Volcano and MA plots; Additional file 5).

### GSE112680 differential expression analysis

The above processing steps yielded an expression matrix for 18,490 protein-coding genes and 376 samples (164 ALS, 137 CTL and 75 MIM samples). Differential expression analyses were performed for two comparisons (1: ALS vs. CTL, 2: MIM vs. CTL). For the ALS vs. CTL comparison, differential expression analyses were performed using 10,670 genes with detectable expression in 20% (61/301) of ALS and CTL samples. For the MIM vs. CTL comparison, differential expression analyses were performed using 10,679 genes with detectable expression in 20% (43/212) of MIM and CTL samples. Raw expression values were adjusted using residual analysis to remove effects of sex as described above. Differential expression testing was performed using linear models and moderated t-statistics (R package: limma; functions: lmFit and eBayes) [50] with correction of raw p-values using the Benjamini–Hochberg method [51]. These steps led to the identification of 1380 ALS-increased DEGs and 1186 ALS-decreased DEGs based upon the GSE112680 dataset (FDR < 0.10, FC > 1.10 or FC < 0.909; Additional file 6). Volcano and MA plots demonstrated that up- and down-regulated FC estimates were symmetrically distributed and not associated with mRNA abundance (Additional file 6).

### Differential expression meta-analysis

A “late stage” meta-analysis data integration framework [46] was used to identify DEGs through integration of summary statistics generated from each cohort, respectively (GSE112676 and GSE112680). 9822 protein-coding genes were assayed in both datasets with detectable expression in at least 20% of GSE112676 samples (> 148/740) and at least 20% of GSE112680 samples (> 61/301). For these 9822 genes, log_2_-adjusted FC estimates and their standard errors from both cohorts were combined using a random effects meta-analysis model, with pooling of FC estimates based upon inverse variance weighting (R package: meta; function: metagen). The mean expression difference between log_2_-normalized expression intensities in ALS and CTL patients was used as the meta-analysis summary measure (equivalent to log_2_-scaled FC estimates). This generated a meta-FC estimate for each of the 9822 genes, with a pooled meta-p-value providing a test for consistent differential expression in both cohorts. Meta-p-values were corrected for multiple hypothesis testing using the Benjamini–Hochberg method [51]. Up- and down-regulated meta-FC estimates were symmetrically distributed and not associated with mRNA abundance based upon inspection of volcano and MA plots (Additional file 7).

### Gene expression responses to riluzole

Riluzole (RZE) is the first-line ALS treatment [52], and since patients in our analyses would likely have taken this drug, some genes with ALS-altered expression may represent RZE responses. To address this possibility, we analyzed microarray data from a previous study of MDA-MB-231 cells (GSE96653) that compared expression between vehicle (DMSO)-treated cells (*n* = 3) and cells treated with 25 µM RZE for 24 h (*n* = 3) [53]. MDA-MB-231 is a breast adenocarcinoma cell line commonly used in cancer research [54]. Although this cell type is not directly related to ALS pathogenesis, we reasoned that RZE expression responses in the MDA-MB-231 cell line may be representative of those in blood-derived cells. Furthermore, expression responses in MDA-MB-231 cells had been profiled using the same Illumina HumanHT-12 V4.0 platform used to measure gene expression in the GSE112680 cohort (see above). This limited platform-based variation and it was possible to apply the same methods for background correction, quantile normalization, probe annotation, and probe filtering. The 6 array samples were mutually similar in terms of median intensity, IQR, and number of genes with detectable expression (P < 0.05, 11,362 on average), and there was no evidence for sample outliers (Additional file 8). Differential expression analyses were performed for 11,868 protein-coding genes with detectable expression in 2 of the 6 samples (R package: limma; functions: lmFit and eBayes) [50], leading to the identification of 255 RZE-increased DEGs (FC > 1.10) and 192 RZE-decreased DEGs (FC < 0.909) at an FDR threshold of 0.10 (Additional file 8).

### Comparison to prior microarray analysis of ALS patient whole blood

The study of van Rheenen et al. [31] included a supplemental data file listing 2593 genes identified as differentially expressed (ALS vs. CTL) in the original analysis of the GSE112676 and GSE112680 datasets (FDR < 0.05 with FC > 1.50 or FC < 0.67). This list was used to assess overlap between ALS DEGs identified in the current study to those identified in the prior analysis [31]. Otherwise, among prior ALS patient blood gene expression studies (Additional file 1), one analysis considered whole blood [32] such that results should be comparable to those from the current analysis. This study had evaluated expression in smaller ALS and CTL patient cohorts (*n* = 30 per group) using the Illumina Sentrix HumanRef-8 Expression BeadChip microarray platform [32]. Raw data from this study has not been submitted to a public database, but a list of genes differentially expressed between ALS and CTL patients has been provided (based upon results from a two-sample t-test with FDR threshold of 0.05; see Additional file 1 from [32]). The supplemental file from this study was filtered to remove redundant or unannotated probes, leading to a filtered set of 2163 genes with differing expression between ALS and CTL patients (1089 ALS-increased and 1074 ALS-decreased). 1584 of these 2163 genes were included in our meta-analysis with detectable expression in both GSE112676 and GSE112680 cohorts (793 ALS-increased and 791 ALS-decreased). Fisher’s exact test was used to evaluate the overlap of these 1584 genes with those identified as altered in ALS patients from the current study. Additionally, using differential expression statistics reported previously [32], we extend the meta-analysis approach described above (R package: meta; function: metagen) to integrate FC estimates and standard errors with those obtained in our analysis (GSE112676 and GSE112680), leading to a “high confidence” set of genes differentially expressed in ALS blood. Results from this extended meta-analysis are included as supplemental material, although in this manuscript we focus on genes identified from the GSE112676 and GSE112680 cohort meta-analysis (for which all raw data are available).

### ALS DEG functional analysis database sources

Functional properties of ALS DEGs were evaluated by testing for enrichment of Gene Ontology [36] and Kyoto Encyclopedia of Genes and Genomes (KEGG) [37] annotations using a conditional hypergeometric test (R package: GOstats; function: hyperGTest) [55]. Non-conditional hypergeometric tests were performed to assess DEG enrichment for Reactome [56] and Disease Ontology (DO) [57] annotations (R packages: ReactomePA and DOSE; functions: enrichPathway and enrichDO) [58, 59]. ALS-associated genes were identified based upon a previous analysis of 9 database resources linking genes to specific diseases [60]. A gene was considered to be ALS-associated if it was linked to ALS based upon at least 2 of the 9 databases included in the analysis [60]. ALS DEGs were additionally assessed for enrichment of annotations included in the Pathway Commons database (R package: paxtoolsr) [61]. ALS DEGs were also evaluated to assess for overlap with gene sets included in the MSigDB database (R package: msigdbr) [62]. The MSigDB database is a collection of annotated gene sets developed to be used for gene set enrichment analysis (GSEA). Our analysis considered the “C7” MSigDB database collection, which includes 4872 gene sets derived from microarray studies of immune cells [62]. In addition to the MSigDB database, ALS expression changes were further compared with those observed in a previously compiled set of 462 gene expression signatures, where each signature was established from comparisons among human PBMC microarray samples available in Gene Expression Omnibus (GEO) [63]. Genetic loci associated with ALS were identified using the NHGRI GWAS catalog [40].

### Analysis of exosome-associated genes

The expression of exosome-associated genes [39, 64] was evaluated to determine if corresponding mRNAs were disproportionately increased or decreased in ALS patients. We evaluated 91 proteins with altered abundance in serum exosomes from ALS patients compared to control subjects (*n* = 3 per group; 41 ALS-increased, 50 ALS-decreased), which had previously been identified using mass spectrometry (see Table S4 from Tomlinson et al. [65]). We also evaluated 83 exosome-associated mRNAs from the ExoCarta database [66] that had been identified in exosomes from at least 4 separate experiments (download file: EXOCARTA_PROTEIN_MRNA_DETAILS_5.txt). We further evaluated a list of the 100 mRNAs most frequently detected in human exosomes compiled by the EVpedia database [67]. Expression of ALS DEGs in blood-derived exosomes was quantitatively compared to that of non-DEGs using TPM-normalized (Transcript Per Million) expression values downloaded from exoRBase [68] (filename: Normal_mRNA_TPM.txt), which had been generated by mapping of mRNA-seq reads (hg38 reference genome) generated by Li et al. [69] (GSE100206). TPM values were averaged across samples from 32 normal subjects [69].

### Whole blood gene expression deconvolution analyses

Since gene expression in unfractionated blood is partly determined by the abundance of constituent immune cells, in silico approaches have been developed to quantify inter-sample variation in cell type abundance based upon gene expression in blood or other whole tissues [70–73]. We assembled a gene expression database to identify genes with cell type-specific expression in 12 immune cell types (neutrophils (NP), monocytes (MC), dendritic cells (DC), macrophages (MP), platelets (PL), red blood cells (RBC), eosinophils (ES), CD4 T cells (CD4), CD8 T cells (CD8), gamma-delta T cells (GDT), B cells (B) and NK cells (NK)). We here define a “cell type-specific expression pattern” as one in which a gene’s expression is quantitatively higher in one cell type as compared to other cell types, even though expression of a gene may be qualitatively detectable in multiple cell types. In this sense, cell type-specific expression is not a binary concept (expressed vs. not expressed) but varies along a continuum. To identify genes exhibiting such a pattern, we assembled a database of samples deposited in the GEO database [74] that had been generated using the Affymetrix Human Genome U133 Plus 2.0 platform. Database samples were identical to those described in a previous analysis [41] for 8 cell types (NP, *n* = 492; MC, *n* = 560; DC, *n* = 491; MP, *n* = 450; CD4, *n* = 574; CD8, *n* = 161; B, *n* = 435; NK, *n* = 160). For the other 4 cell types, additional samples were added to the database (PL, *n* = 48; RBC, *n* = 41; ES, *n* = 7; GDT, *n* = 17), but procedures followed for normalization and identification of cell type-specific genes were otherwise consistent with those previously described [41]. We refer to the RBC group as “RBC/erythroid lineage cells”, since experiments used for gene expression analysis evaluated RBC precursors such as erythroblasts and reticulocytes (see GEO accessions GSE17639, GSE18679, GSE22552, GSE24849, GSE65577).

Samples for each cell type were normalized using Robust Multichip Average (RMA) [75], and if more than 50 samples were available for a given cell type, the 50 samples with lowest average Euclidean distance to all other samples were selected as representatives [41]. Following normalization and sample selection, cell type-specific genes were identified by two-group comparisons between each cell type and all samples associated with the other 11 cell types (R package: limma; functions: lmFit and eBayes) [41]. To select signature genes for a given cell type, we first identified the set of 150 genes with elevated expression in that cell type with lowest p-values from the two-group comparison. These 150 genes were then sorted based upon FC (target cell type/other cell types), and the 100 genes with highest FC were selected as signature genes for the target cell type. Given these thresholds, the final set of 100 signature genes chosen for each cell type exhibited higher expression in samples from the target cell type when compared to the pooled set of samples from the 11 other cell types, with no less than 2.48-fold elevated expression compared to the 11 other cell types (P ≤ 1.64e−07 in all comparisons). In every case, median expression of the 100 signature genes was at least 60% greater in the target cell type than median expression in every other cell type individually (Additional file 9).

To obtain signature scores for each blood sample, we calculated the (weighted) average of *Z*-score normalized expression estimates for the 100 signature genes identified for each cell type. This average was calculated with greater weight assigned to genes with a stronger cell type-specific expression pattern (R function: weighted.mean). Preliminary weights assigned to each gene were equal to *w*^1/2^, with *w* = 100 for the top-ranked gene, *w* = 99 for the second-ranked gene, *w* = 98 for the third ranked gene, and so on. Preliminary weights were then scaled to the [0, 1] interval by dividing preliminary weights by the maximum value, i.e., (100)^1/2^, yielding final weights used for the weighted average signature score calculation [41].

These same procedures were followed to calculate M1 and M2 macrophage signature scores based upon a previously published dataset (GSE5099) [76]. M1 signature genes were identified from the two-group comparison between M1 macrophages (GSM115055–GSM115057, GSM115070–GSM115072) and the combined set of M2 and non-polarized macrophages (GSM115052–GSM115054, GSM115058–GSM115060). Similarly, M2 signature genes were identified by comparing expression between M2 macrophages (GSM115058–GSM115060, GSM115073–GSM115075) and the combined set of M1 and non-polarized macrophages (GSM115052–GSM115057, GSM115067–GSM115072). Normalization and differential expression analyses were performed as described above using RMA and linear models (R package: limma; functions: lmFit and eBayes). Experiments were performed using two early-generation Affymetrix microarray platforms (U133A and U133B). Differential expression analyses were thus performed for each platform separately, followed by late-stage integration of differential expression summary statistics [46], excluding any genes from the U133B analysis that were already included from analysis of the more comprehensive U133A platform. As above, signature genes were identified by first selecting the 150 genes with lowest p-values in two-group comparisons (M1 vs. other, M2 vs. other), and of these the 100 genes with high FC were chosen as signature genes (M1/other or M2/other). M1 and M2 signature scores were then calculated for each blood sample as described above, with weighted averaging of Z-score normalized expression of signature genes, with increased weight assigned to those genes having expression most specific to M1 or M2 macrophages.

Alternative methods have been proposed for cell type deconvolution, which utilize different algorithms and cell type marker genes chosen from compiled databases [70–73]. Results obtained using the above approach were thus compared with those obtained using the ImSig algorithm, which calculates sample-specific cell type scores based on a different algorithm with independently identified cell type marker genes (R package: ImSig) [70].

### Accuracy of gene expression for predicting ALS diagnosis

Diagnostic accuracy was defined as the ability of blood gene expression measures to discriminate ALS from non-ALS patients (MIM and CTL). Area under the curve (AUC) statistics were calculated to assess diagnostic accuracy based upon the expression of individual genes (R library: pROC, function: ci.auc) [77]. Cross-validation analyses were performed using randomly chosen training (296 ALS patients vs. 296 CTL/MIM subjects) and testing sets (100 ALS patients vs. 100 CTL/MIM subjects) with 10,000 simulation trials. For individual genes, classification models were generated from training data using logistic regression (R function: glm). For multigene models, classification models were generated from training data using the random forest algorithm (R package: randomForest; R function: randomForest) [78], logistic regression [79] (R function: glm; binomial error distribution), and support vector machines [80] (R package: e1071; function: svm). In each simulation trial, accuracy, sensitivity and specificity were calculated (R library: caret; function: confusionMatrix) and McNemar’s Chi squared test was used to test the hypothesis of confusion matrix marginal homogeneity (R function: mcnemar.test) [81]. We then calculated the proportion of significant McNemar tests and average accuracy, sensitivity and specificity across the 10,000 simulation trials.

### Accuracy of gene expression for predicting ALS patient survival

Cox proportional hazard (PH) models were used to evaluate the significance of gene expression-survival associations and estimate corresponding hazard ratios in covariate-adjusted models (R library: survival; function: coxph). Analyses were performed for 11,480 protein-coding genes with detectable expression in at least 20% of ALS patients (> 80/396). All Cox PH models included age, sex, site of onset, and cohort as covariates, with additional variables corresponding to the expression of one or more genes. A multigene Cox PH model was developed using stepwise variable selection with p-value of 0.15 required for variables to enter or remain within the regression model (R library: My.stepwise; function: My.stepwise.coxph). To assess survival prediction accuracy using cross-validation, 10,000 simulation trials were performed, with patients randomly assigned to a training (*n* = 296) or test set (*n* = 100) in each trial. The training set was used to estimate coefficients for a Cox PH model, which was then applied to the testing set, yielding linear predictor scores used to stratify patients in terms of predicted survival (function: predict.coxph). Correspondence between linear predictors and test set survival times was evaluated using the AUC concordance index proposed by Heagerty and Zheng (R library: risksetROC; function: risksetAUC) [82]. For any two randomly chosen test set patients, this index estimates the probability that Cox PH model outputs successfully determine which patient survives longer [82, 83]. A total of 10,000 simulation trials were performed and the average concordance index was determined. The average concordance was compared between a base model (clinical covariates only) and a full model (clinical covariates + gene expression) to assess the contribution of expression variables to survival prediction accuracy.

## Results

### Genes with elevated expression in ALS blood are associated with ribosomes, translation and neutrophil activation

**Fig. 1:**
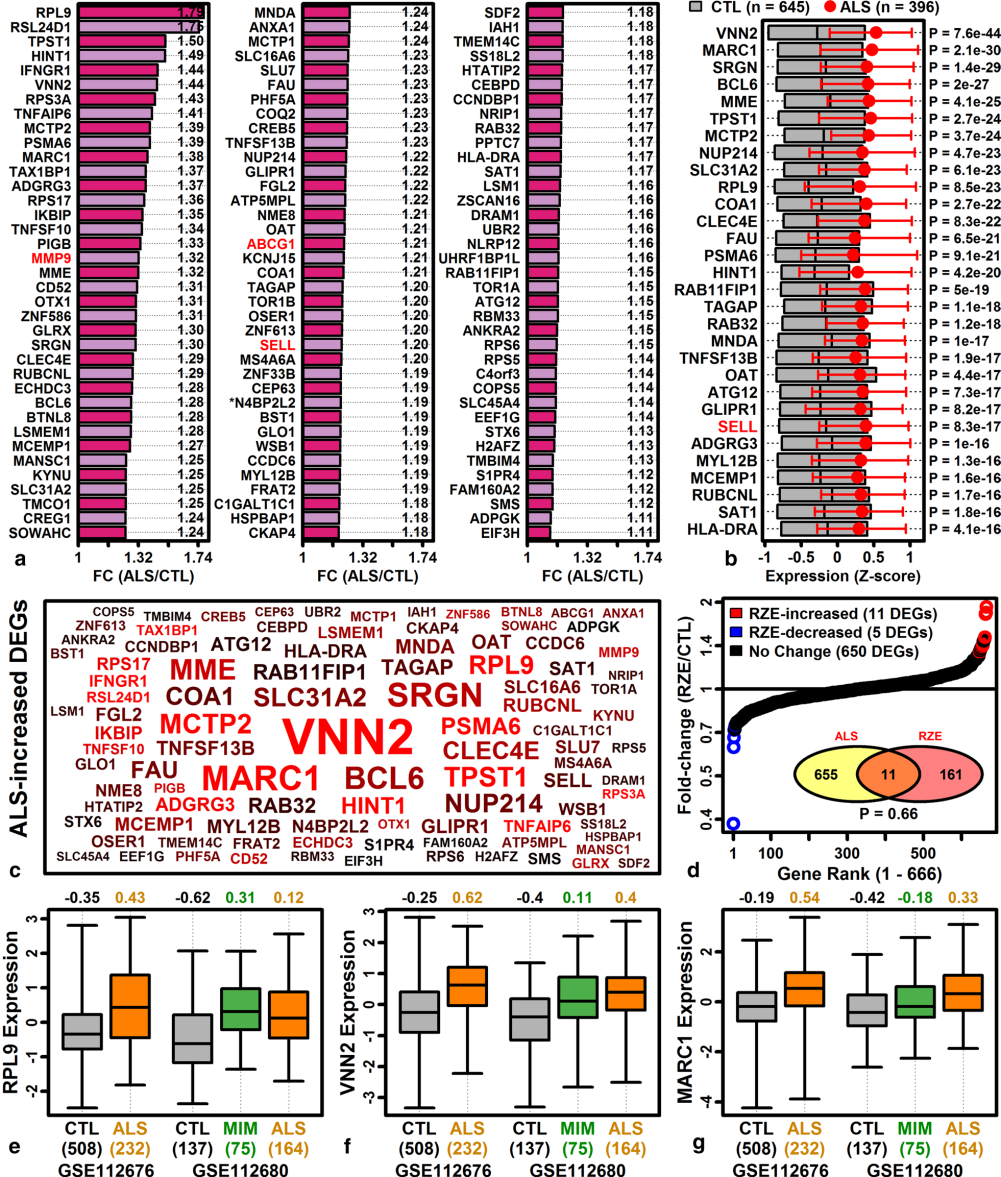
Top-ranked ALS-increased DEGs. **a** Top ALS-increased DEGs ranked by FC (red font: ALS-associated genes; *riluzole-increased DEG, FDR < 0.10). **b** Top ALS-increased DEGs ranked by p-value. *Z*-score normalized expression values were combined across cohorts. Grey boxes outline the middle 50% of CTL expression values (midpoint: median), and magenta error bars outline the middle 50% of ALS expression values (circle: median). **c** ALS-increased DEG symbol cloud. Gene symbol size is inversely proportional to differential expression analysis p-values (ALS vs. CTL) and colors are proportional to FC estimates (black: lower FC, red: higher FC). **d** Riluzole (RZE) effects on ALS-increased DEGs (GSE96653, MDA-MB-231 cells). FC estimates are plotted for 666 ALS-increased DEGs (red symbols: RZE-increased, FDR < 0.10, FC > 1.10; blue symbols: RZE-decreased: FDR < 0.10, FC < 0.91). The Venn diagram (bottom-right) shows the overlap between ALS-increased and RZE-increased DEGs (p-value: Fisher’s exact test). **e** Ribosomal protein L9 (*RPL9*) expression. **f** Vanin 2 (*VNN2*) expression. **g** Mitochondrial amidoxime reducing component 1 (*MARC1*) expression. In **e**–**g**, *Z*-score normalized expression values are shown for each cohort (boxes: middle 50% of values; whiskers: 10th to 90th percentiles) with sample sizes in each group (bottom margin, parentheses)

Meta-analysis identified 752 ALS-increased DEGs (FDR < 0.10 with FC > 1.10) with a consistent differential expression pattern in both cohorts (GSE112676 and GSE112680) (Additional file 10A). Genes most strongly elevated in ALS blood included ribosomal protein L9 (*RPL9*), ribosomal L24 domain containing 1 (*RSL24D1*), vanin 2 (*VNN2*) and mitochondrial amidoxime reducing component 1 (*MARC1*) (Fig. 1). Several top-ranked ALS-increased DEGs had previously been associated with ALS, such as matrix metallopeptidase 9 (*MMP9*) [84], ATP binding cassette subfamily G member 1 (*ABCG1*) [85], and selectin L (*SELL*/*CD62L*) [86] (Fig. 1a, b). There was no significant overlap between ALS-increased DEGs and genes up-regulated in MDA-MB-231 cells following riluzole treatment (25 µM for 24 h; P = 0.66; Fig. 1d) [53]. ALS-increased DEGs were most strongly enriched with respect to ribosome-associated genes (Additional file 11) and were additionally associated with translation and neutrophil activation (Additional file 11A). Comparison to MSigDB gene sets [62] showed that ALS-increased DEGs overlapped significantly with genes elevated in neutrophils compared to B or T cells (Additional file 11G). ALS-increased DEGs were also significantly associated with immune cell activation, viral transcription, and RAGE receptor binding (Additional file 11).

The 752 ALS-increased DEGs were compared to those identified in the original analysis of van Rheenen et al. [31]. Of the 752 ALS-increased DEGs, 208 (28%) had been identified as differentially expressed in the original analysis (P = 5.6e−26; Fisher’s exact test). We next compared the 752 DEGs we identified to DEGs from the prior study of Saris et al. [32], which compared whole blood gene expression in ALS patients and controls using a different set of samples (*n* = 30 per group) and different microarray platform. Of the 752 ALS-increased DEGs, 225 (29.9%) had been reported as differentially expressed by Saris et al. [32], and nearly all of these (224/225) were ALS-increased as observed in the current study (P = 2.1e−76, Fisher’s exact test; Additional file 12A). Conversely, the complete set of ALS-increased DEGs reported by Saris et al. [32] was biased towards increased expression in ALS blood samples from the current study (P = 1.51e−183, Wilcoxon rank sum test; Additional file 12B). These results demonstrate good directional consistency between our results and those reported by Saris et al. [32]. A ranked list of “high confidence” ALS-increased genes, generated from meta-analysis of results from the current study and results from Saris et al. [32], is included in supplemental materials with gene set enrichment analysis findings (Additional file 12C–E).

### Genes with reduced expression in ALS blood are associated with TGF-beta responses, anemia, bleeding disorders, and RSV infection

**Fig. 2:**
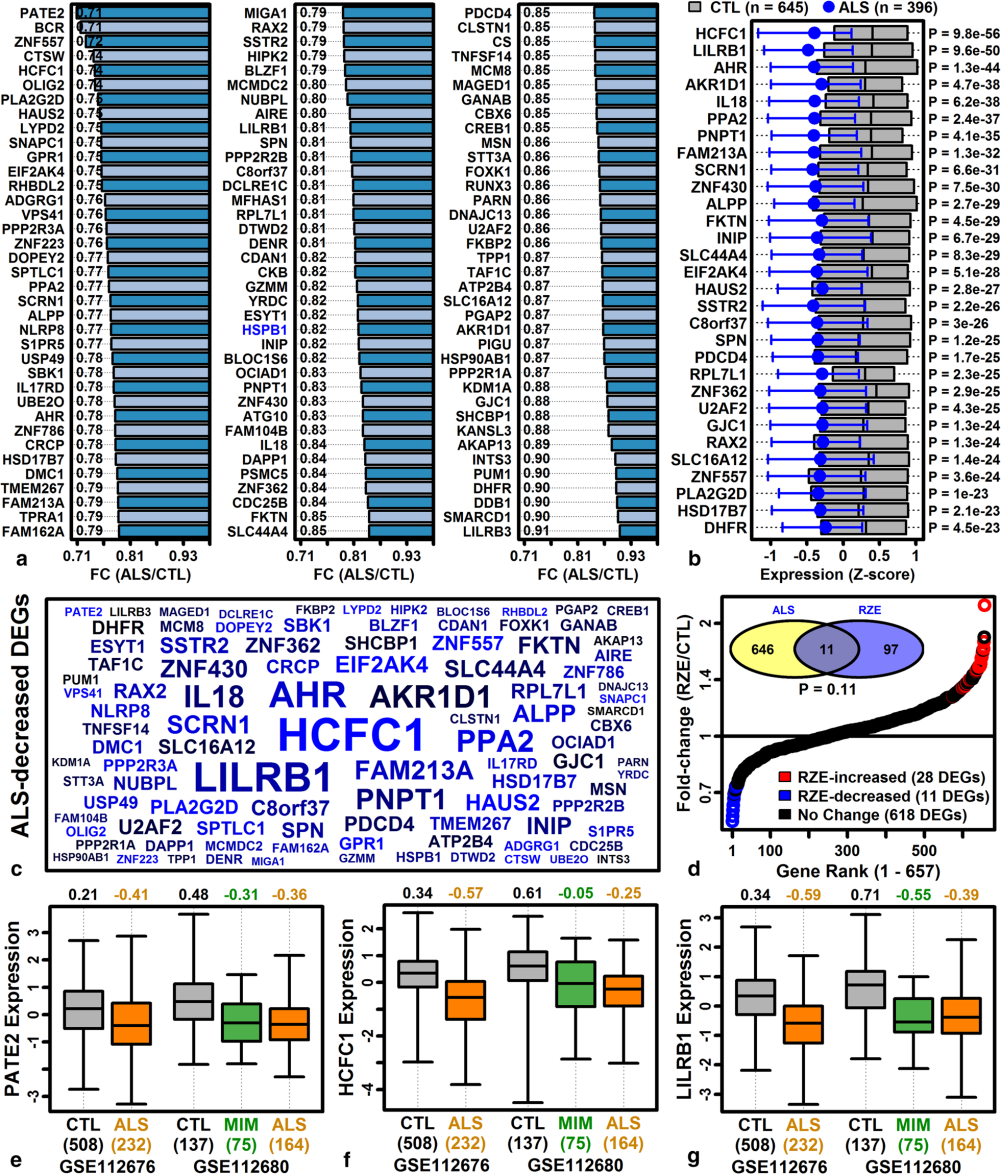
Top-ranked ALS-decreased DEGs. **a** Top ALS-decreased DEGs ranked by FC (blue font: ALS-associated genes). **b** Top ALS-decreased DEGs ranked by p-value. *Z*-score normalized expression values were combined across cohorts. Grey boxes outline the middle 50% of CTL expression values (midpoint: median), and magenta error bars outline the middle 50% of ALS expression values (circle: median). **c** ALS-decreased DEG symbol cloud. Gene symbol size is inversely proportional to differential expression analysis p-values (ALS vs. CTL) and colors are proportional to FC estimates (black: higher FC, blue: lower FC). **d** Riluzole (RZE) effects on ALS-decreased DEGs (GSE96653, MDA-MB-231 cells). FC estimates are plotted for 657 ALS-decreased DEGs (red symbols: RZE-increased, FDR < 0.10, FC > 1.10; blue symbols: RZE-decreased: FDR < 0.10, FC < 0.91). The Venn diagram (top-left) shows the overlap between ALS-decreased and RZE-decreased DEGs (p-value: Fisher’s exact test). **e** Prostate and testis expressed 2 (*PATE2*) expression. **f** Host cell factor C1 (*HCFC1*) expression. **g** Leukocyte immunoglobulin like receptor B1 (*LILRB1*) expression. In **e**–**g**, *Z*-score normalized expression values are shown for each cohort (boxes: middle 50% of values; whiskers: 10th to 90th percentiles) with sample sizes in each group (bottom margin, parentheses)

Meta-analysis identified 764 ALS-decreased DEGs (FDR < 0.10 with FC < 0.909) (Additional file 10B). Genes most strongly decreased in ALS blood included prostate and testis expressed 2 (*PATE2*), BCR, RhoGEF and GTPase activating protein (*BCR*), host cell factor C1 (*HCFC1*), and leukocyte immunoglobulin like receptor B1 (*LILRB1*) (Fig. 2). At least one top-ranked ALS-decreased gene had previously been associated with ALS (i.e., heat shock protein family B small member 1, *HSPB1*; Fig. 2a) [87]. Overlap between ALS-decreased DEGs and genes down-regulated in MDA-MB-231 cells following riluzole treatment (25 µM for 24 h) was non-significant (P = 0.11; Fig. 2d) [53]. Genes decreased in ALS patient blood were associated with TGF-beta response, Z disc, antigen processing/presentation, and platelet/clotting disorders (e.g., blood platelet disease, hemorrhagic thrombocythemia, congenital hemolytic anemia) (Additional file 13). The strongest association was significant overlap between ALS-decreased genes and genes up-regulated in PBMC from infants with acute respiratory syncytial virus (RSV) infection (as compared to infants with influenza; Additional file 13G).

The 764 ALS-decreased DEGs were compared to those identified in the original analysis of van Rheenen et al. [31]. 236 of the 764 (30.9%) had been identified as differentially expressed in the original analysis (P = 1.55e−37; Fisher’s exact test). The 764 DEGs were next compared to those identified in the prior microarray study performed by Saris et al. using a different sample set (*n* = 30 per group) [32]. Of the 764 ALS-decreased DEGs, 186 (24.3%) had been identified as differentially expressed by Saris et al. [32], and the majority of these (174/186) were ALS-decreased as in the current analysis (P = 1e−43, Fisher’s exact test; Additional file 14A). The complete set of ALS-decreased genes identified by Saris et al. [32] was biased towards ALS-decreased expression in the current study (P = 7.7e−140, Wilcoxon rank sum test; Additional file 14B). High confidence ALS-decreased genes identified from meta-analysis of results from the current analysis and prior study of Saris et al. [32] are listed with further analysis in supplemental materials (Additional file 14C–E).

### ALS blood has increased expression of neutrophil-expressed genes but decreased expression of genes expressed by RBC lineage cells

**Fig. 3:**
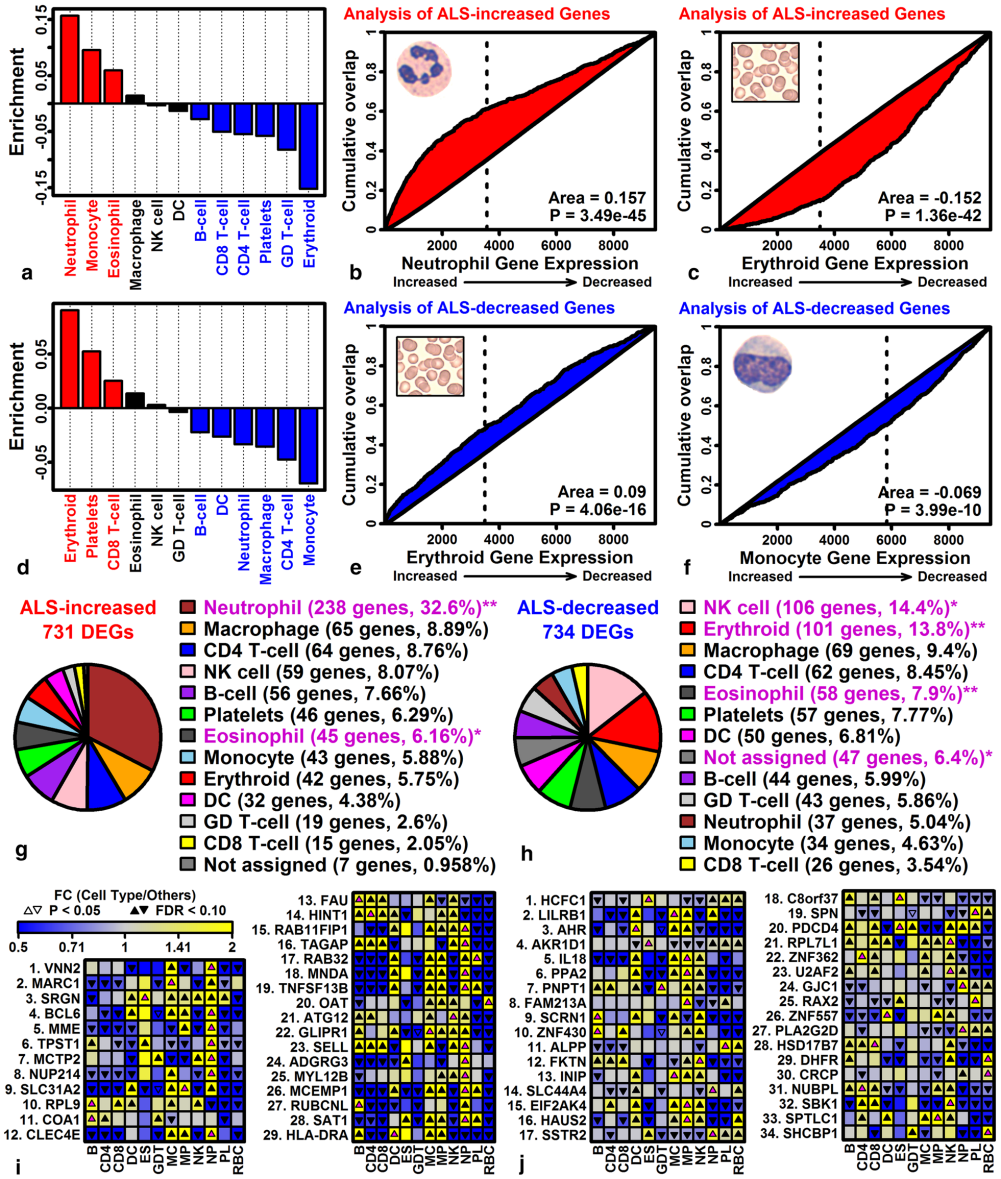
Cell types associated with ALS-increased and ALS-decreased DEGs. **a** Enrichment statistics for 12 cell types (ALS-increased DEGs). **b** Neutrophil GSEA analysis (ALS-increased DEGs). **c** RBC lineage GSEA analysis (ALS-increased DEGs). **d** Enrichment statistics for 12 cell types (ALS-decreased DEGs). **e** RBC lineage GSEA analysis (ALS-decreased DEGs). **f** Monocyte GSEA analysis (ALS-decreased DEGs). In **a**, **d**, positive statistics indicate over-representation of cell type-specific genes among ALS DEGs (P < 0.05, red bars), and negative statistics indicate under-representation of cell type-specific genes among ALS DEGs (P < 0.05, blue bars). In **b**, **c**, **e** and **f**, genes are ranked according to their expression in the indicated cell type (horizontal axis), and cumulative abundance of ALS DEGs is shown (vertical axis). The area (lower-right) between the cumulative abundance curve and diagonal is equal to enrichment statistics shown in parts A and D (p-values: Wilcoxon rank sum test). **g** Cell type assignments (ALS-increased DEGs). **h** Cell type assignments (ALS-decreased DEGs). In **g**, **h**, genes were assigned to the cell type for which they were most highly expressed as compared to other cell types. Pie charts denote the proportion of genes assigned to each cell type (*P < 0.05; **FDR < 0.05, Fisher’s exact test). Genes were not assigned to any cell type if expression was not detectable in at least 10% of samples for any cell type (P < 0.05, Signed rank test). **i** Top-ranked ALS-increased DEGs and their expression across 12 cell types. **j** Top-ranked ALS-decreased DEGs and their expression across 12 cell types. In **i** and **j**, magenta up-triangles denote the cell type with highest expression for each gene

Annotation-based enrichment analyses showed that ALS-increased DEGs were linked to neutrophil-associated terms (Additional file 11A), whereas ALS-decreased DEGs were linked to anemia and bleeding disorders (Additional file 13F). To dissect this further, DEGs were compared to lists of genes specifically expressed in 12 peripheral blood cell types [41]. Among the 12 cell types, the 752 ALS-increased DEGs were most significantly enriched for genes with neutrophil-specific expression (Fig. 3a, b), whereas ALS-increased DEGs included very few genes with RBC lineage-specific expression (Fig. 3a, c). An opposite pattern was observed among ALS-decreased DEGs, which were enriched for RBC lineage-specific genes but few neutrophil-specific genes (Fig. 3d, e). Overall, 32.6% of ALS-increased DEGs (238/731) had higher expression in neutrophils than any other cell type (Fig. 3g), while 13.8% of ALS-decreased DEGs (101/734) had higher expression in RBC lineage cells than any other cell type (Fig. 3h). ALS-increased DEGs most highly expressed in neutrophils included *VNN2*, *BCL6* and *MME* (Fig. 3i), and ALS-decreased DEGs most highly expressed in RBC lineage cells included *DHFR*, *CRCP*, and *SHCBP1* (Fig. 3j).

The analysis was repeated using the ImSig database [70], which provides signature gene sets for 8 cell types and 2 biological processes (translation and proliferation) with calculation of scores based upon signature gene expression and co-expression. This again showed that neutrophil signature scores were most strongly elevated in ALS patients, with significant elevation of translation, plasma cell and macrophage scores as well (Additional file 15A). In contrast, NK cell signature scores were reduced in ALS patients (Additional file 15A), in agreement with over-abundance of NK cell-specific genes among ALS-decreased DEGs (Fig. 3h). 13 of the ImSig neutrophil signature genes were ALS-increased DEGs, whereas none were ALS-decreased DEGs (Additional file 15D, E). Similarly, 29 translation signature genes were ALS-increased DEGs and none were ALS-decreased DEGs (Additional file 15F, G).

### ALS-increased DEGs have high blood exosome expression and ALS-decreased DEGs have low blood exosome expression

Enlargement of blood-derived exosomes was recently demonstrated in ALS patients [38], and proteins with altered abundance in blood exosomes from ALS patients have been identified [65]. We compared mRNA FC estimates in the current study to those obtained previously for exosome proteins altered in ALS patients compared to CTL subjects [65], which revealed a weak positive FC correlation (*r*_s_ = 0.16, P = 0.25) and 5 mRNA-protein pairs with consistent changes in abundance (ALS-increased: *THBS1*; ALS-decreased: *DPYSL5*, *SLC4A1*, *TTN*, *TLN1*) (Additional file 16A). We next identified exosome-associated mRNAs from the ExoCarta [66] and EVpedia [67] databases and showed that these were biased towards ALS-increased expression (P ≤ 6.3e−3; Additional file 16B, C). Genes with higher expression in blood-derived exosomes from normal subjects (*n* = 32) [68, 69] were also more likely to be elevated in ALS blood (Additional file 16D). Conversely, genes most strongly elevated in ALS blood tended to have high blood exosome expression (Additional file 16E), while genes most strongly decreased in ALS blood had lower blood exosome expression (Additional file 16F). ALS-increased DEGs most highly expressed in blood-derived exosomes encoded ribosomal subunits and other translation-associated proteins (e.g., *EEF1A1*, *RPL13A*, *RPS6*; Additional file 16G, H).

### The ALS blood transcriptome broadly resembles expression responses to acute high altitude stress

**Fig. 4:**
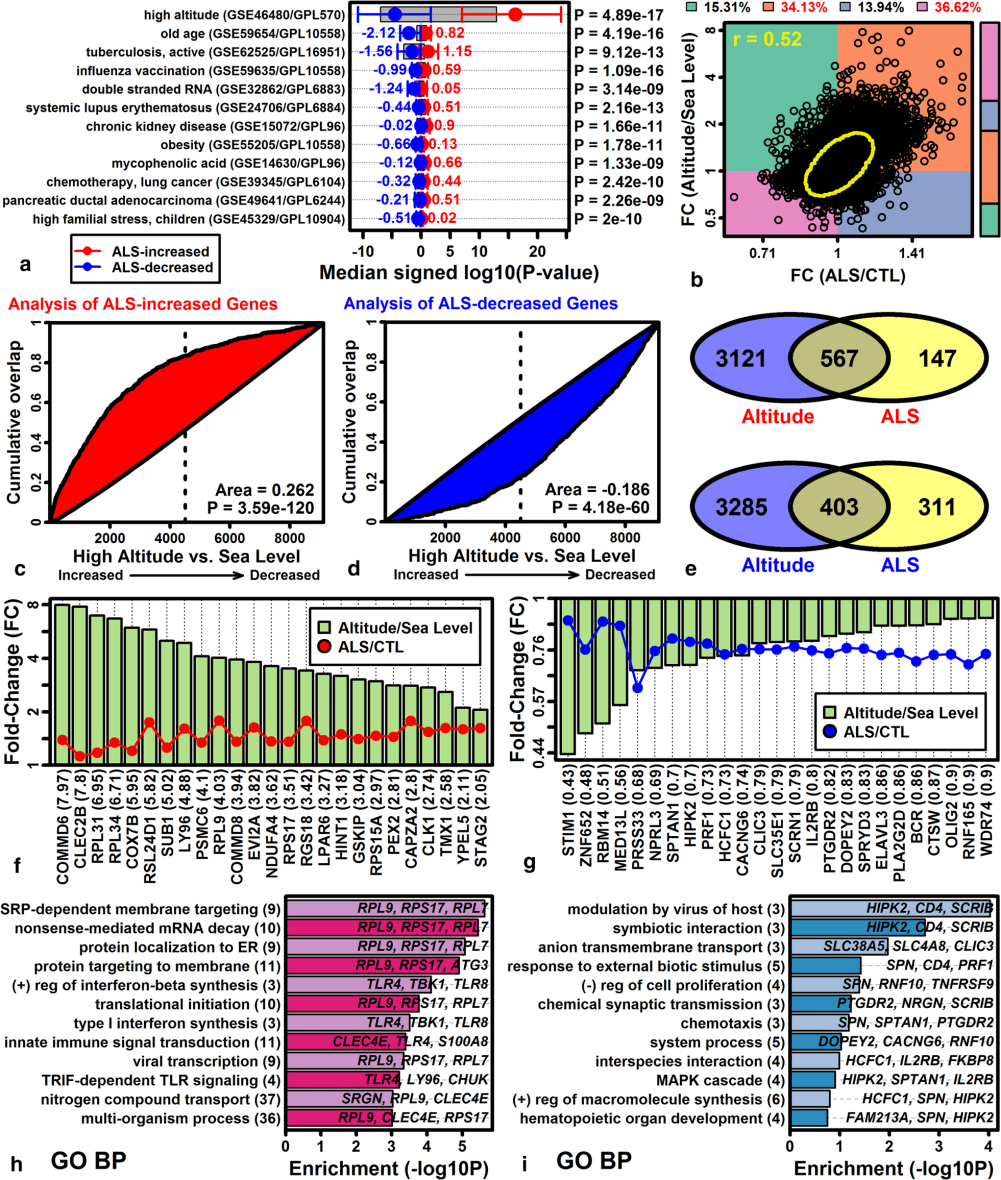
PBMC gene expression signatures resembling the ALS transcriptome. **a** Top 12 ALS-matching PBMC signatures. 422 PBMC gene expression signatures were screened to identify those with elevated expression of ALS-increased DEGs and decreased expression of ALS-decreased DEGs. Expression shifts in each signature were quantified using signed log_10_-transformed p-values (Log10P; positive values: increased expression; negative values: decreased expression). Round symbols represent the median Log10P for the 100 genes most strongly elevated in ALS patients (red), and the median Log10P for the 100 genes most strongly decreased in ALS patients (blue). Whiskers outline the middle 50% of Log10P values (25th to 75th percentile). **b** High altitude versus ALS scatterplot (9130 genes). FC estimates are shown from the comparison of blood samples obtained at high altitude versus sea level (vertical axis), and from the comparison of samples from ALS versus control subjects (horizontal axis). The yellow ellipse outlines the middle 50% of genes closest to the bivariate median (Mahalanobis distance). The spearman rank correlation coefficient is shown (upper-left). The percentage of genes in each quadrant is indicated (top margin; red font: P < 0.05, Fisher’s exact test). The color-coded vertical bar (right margin) reflects the proportion of genes in each quadrant. **c** High altitude GSEA analysis (ALS-increased DEGs). **d** High altitude GSEA analysis (ALS-decreased DEGs). In **c**, **d**, genes were ranked according to their expression change in the comparison between subjects at high altitude and sea level (horizontal axis), and cumulative overlap between the ranked gene list and ALS DEGs is shown (vertical axis; p-values: Wilcoxon rank sum test). **e** Venn diagrams. Top: Overlap between ALS-increased and altitude-increased DEGs (FDR < 0.10). Bottom: Overlap between ALS-decreased and altitude-decreased DEGs (FDR < 0.10). **f** Genes increased in ALS patients and high altitude subjects. **g** Genes decreased in ALS patients and high altitude subjects. In **f**, **g**, FC estimates obtained from the comparison between high altitude and sea level subjects is shown in parentheses (bottom margin). **h** Gene ontology biological process terms enriched among 144 genes with increased expression in ALS patients and high altitude subjects (FDR < 0.10, FC > 1.25). **i** Gene ontology biological process terms enriched among 34 genes with decreased expression in ALS patients and high altitude subjects (FDR < 0.10, FC < 0.83)

Gene expression shifts in ALS blood were compared to 462 signatures derived from human PBMC gene expression datasets [63]. This was a hypothesis-generating analysis for which the goal was to determine if ALS-like expression patterns could be discerned from existing datasets, which may help to explain patterns observed in our study. There was significant correspondence to PBMC signatures associated with old age, active tuberculosis and influenza vaccination (Fig. 4a). However, the strongest match was obtained with respect to a gene expression signature derived from the comparison of PBMC from subjects at sea level to those rapidly transported to high altitude (GSE46480; P = 4.89e−17; Wilcoxon rank sum test; Fig. 4a). Consistent with this, there was a significant genome-wide correlation between ALS/CTL and high altitude/sea level FC estimates (*r*_s_ = 0.52; Fig. 4b). The 752 ALS-increased DEGs were significantly enriched for genes elevated with altitude stress (Fig. 4c, and likewise, the 764 ALS-decreased DEGs were enriched for genes decreased by altitude stress (Fig. 4d). Among 714 ALS-increased DEGs assayed in both experiments, 567 (79.4%) were elevated in high altitude subjects (Fig. 4e). Likewise, among 714 ALS-decreased DEGs assayed in both experiments, 403 (56.4%) were decreased in high altitude subjects (Fig. 4e). ALS-increased genes most strongly elevated in high altitude subjects included COMM domain containing 6 (*COMMD6*), C-type lectin domain family 2 member B (*CLEC2B*), and ribosomal protein L31 (*RPL31*) (Fig. 4f) and as a group these genes were associated with protein targeting to membrane, mRNA decay and interferon-beta synthesis (Fig. 4h). ALS-decreased genes most strongly decreased in high altitude subjects included stromal interaction molecule 1 (*STIM1*), zinc finger protein 652 (*ZNF652*) and RNA binding motif protein 14 (*RBM14*), and collectively such genes were associated with modulation by virus of host, anion transmembrane transport, and the MAPK cascade (Fig. 4i).

### Genes with elevated expression in ALS blood overlap significantly with genes near ALS susceptibility loci

**Fig. 5:**
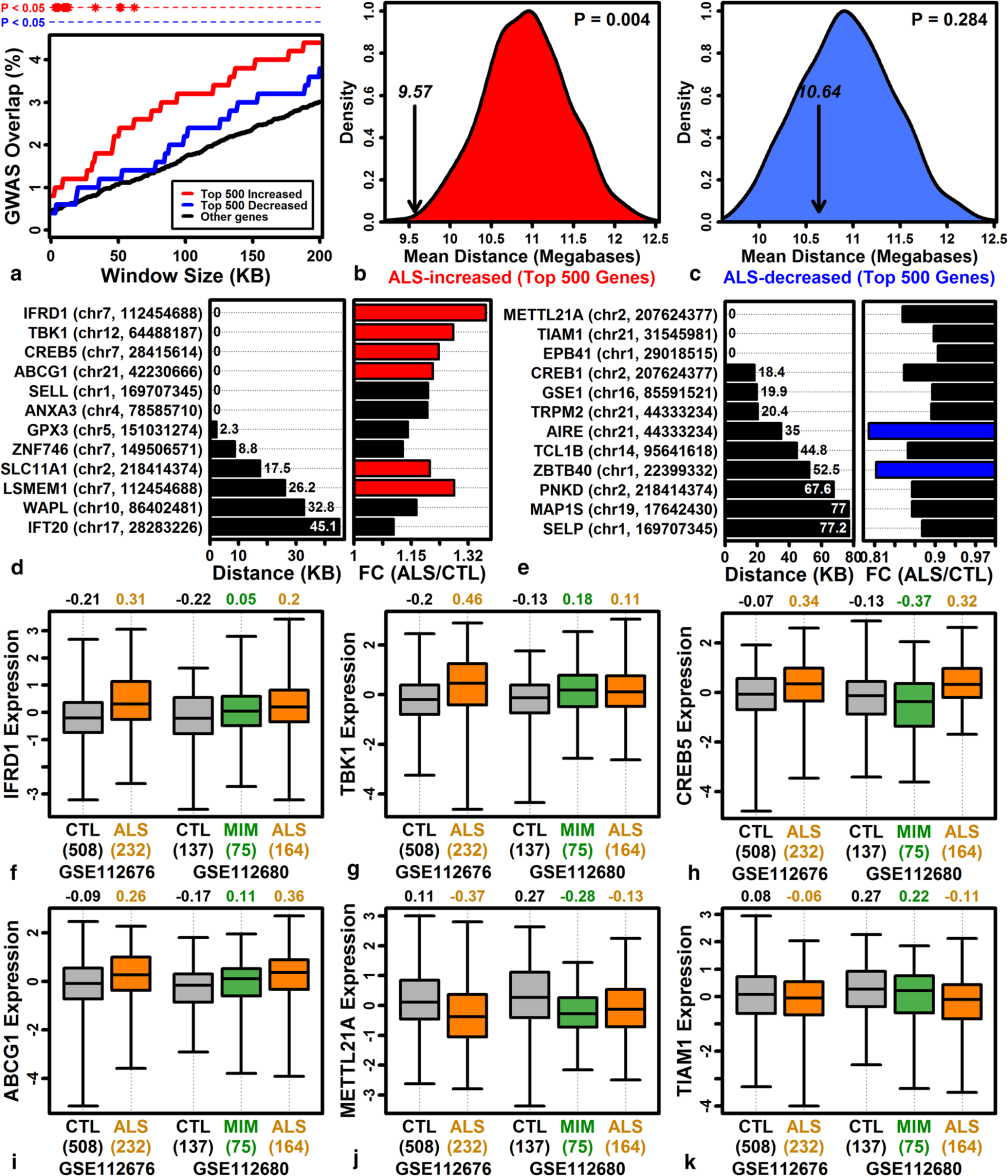
ALS transcriptome overlap with genes near GWAS loci. **a** ALS transcriptome-GWAS overlap. The 500 genes with most strongly increased (red) or decreased (blue) expression in ALS patients were evaluated for overlap (vertical axis) with genes at varying distances from 215 ALS GWAS loci (horizontal axis). **b**, **c** Average distance between ALS DEGs and the nearest GWAS locus. Figures show null distributions for the distance obtained when sampling sets of 500 genes at random (10,000 simulation trials). Vertical arrows denote average distance among the 500 genes most strongly **b** increased or **c** decreased in ALS (p-values: upper-right). **d**, **e** ALS DEGs near GWAS loci. DEGs are listed in the left margin with chromosome and nearest GWAS loci in parentheses. Figures show the distance to the nearest GWAS locus and FC estimate (red: FC > 1.20; blue: FC < 0.83). **f**–**k** Expression of ALS DEGs near GWAS loci. Expression values were Z-score normalized for each cohort (boxes: middle 50% of expression value; whiskers: 10th to 90th percentiles). The sample size for each group is shown in parentheses (bottom margin)

It has been unclear whether genes with altered expression in ALS blood are simply responses to disease progression or instead involved with disease-causing pathogenetic mechanisms [88]. To address this, we evaluated overlap between the top 500 ALS increased/decreased DEGs and genes near ALS GWAS susceptibility loci [40, 89]. ALS-increased DEGs overlapped significantly with genes near susceptibility loci (distance ≤ 62 kb; P < 0.05, Fisher’s exact test), but did not overlap significantly with genes distant from susceptibility loci (distance > 62 kb) (Fig. 5a). For instance, among the top 500 ALS-increased DEGs, 6 of 500 (1.2%) overlapped significantly with genes less than 9 kb from ALS GWAS loci, and this fraction was significantly greater than observed for non-DEGs, i.e., only 49 of 8790 (0.56%) non-DEGs were within 9 kb of a GWAS locus (P = 0.028, Fisher’s exact test). Consistent with this, the average distance between each ALS-increased DEG and its nearest susceptibility locus was significantly reduced compared to randomly sampled genes (P = 0.004; Fig. 5b). In contrast, ALS-decreased DEGs were not significantly more likely to be near a GWAS locus, although similar trends were noted in both analyses (Fig. 5a, c). ALS-increased DEGs nearest to susceptibility loci included interferon related developmental regulator 1 (*IFRD1*), TANK binding kinase 1 (*TBK1*), cAMP responsive element binding protein 5 (*CREB5*), ATP binding cassette subfamily G member 1 (*ABCG1*), selectin L (*SELL*) and annexin A3 (*ANXA3*) (Fig. 5d, f–j). ALS-decreased DEGs nearest to susceptibility loci included methyltransferase like 21A (*METTL21A*), T cell lymphoma invasion and metastasis 1 (*TIAM1*) and erythrocyte membrane protein band 4.1 (*EPB41*) (Fig. 5e, j, k).

### Support vector machines distinguish ALS, CTL and MIM subjects with 87% accuracy based upon blood gene expression (sensitivity 86%, specificity 87%)

Prior studies have suggested that diagnostic blood indicators for ALS may include increased abundance of phosphorylated neurofilament heavy chain (pNFH) and neurofilament light chain (NFL) proteins [6, 90]. We evaluated whether expression of corresponding genes (*NEFH* and *NEFL*) could discriminate ALS and non-ALS (CTL + MIM) subjects, but cross-validation analyses showed that expression of these genes could predict diagnosis with only 49–53% accuracy (logistic regression; Additional file 17A–C). We next searched for single genes for which low or high expression might be diagnostic, focusing on ALS-increased DEGs with low MIM/CTL FC estimates (e.g., *MCTP2, MGAM, CREB5*; Additional file 17D) and ALS-decreased DEGs with high MIM/CTL FC estimates (e.g., *LDHB, METTL16, FAM102A*; Additional file 17E). All such genes had significant AUC statistics for discrimination between ALS and non-ALS subjects (Additional file 17F, G). The highest AUCs (≥ 0.66) were calculated for multiple C2 and transmembrane domain containing 2 (*MCTP2*) and RNA polymerase III subunit C (*POLR3C*) (Additional file 17F, G). Cross-validation testing showed that expression of these genes could predict ALS diagnosis with 62–63% accuracy (logistic regression; Additional file 17H–J).

Genes with altered expression in ALS blood were specifically expressed by neutrophils and RBC-lineage cells, and ALS-like expression shifts were observed in subjects transported to high altitudes (Figs. 3 and 4). Signature scores for immune cell types and altitude stress were calculated and several had significant AUC statistics (P < 0.05), with highest AUCs obtained for neutrophil (0.64), NK cell (0.60) and eosinophil (0.58) scores (Additional file 18A). Closer inspection of the neutrophil signature showed it was elevated in ALS patients, but not similarly increased in MIM subjects (Additional file 18B), and cross-validation analyses showed that neutrophil score could predict ALS diagnosis with 60% accuracy (logistic regression; Additional file 18C). The ratio of neutrophils to monocytes has previously been shown to predict ALS diagnosis correlate with disease progression [91]. Along these lines, combining neutrophil scores with those from other cell types in bivariate models improved AUCs slightly (+monocytes: 0.642, +macrophage: 0.655; +eosinophils: 0.660). However, among bivariate score combinations, the highest AUC was obtained for models combining neutrophil and altitude stress scores (Additional file 18D). These two scores were negatively correlated among patients (*r* = − 0.21; Additional file 18E) and together predicted ALS diagnosis with 64% accuracy (logistic regression; Additional file 18F).

**Fig. 6:**
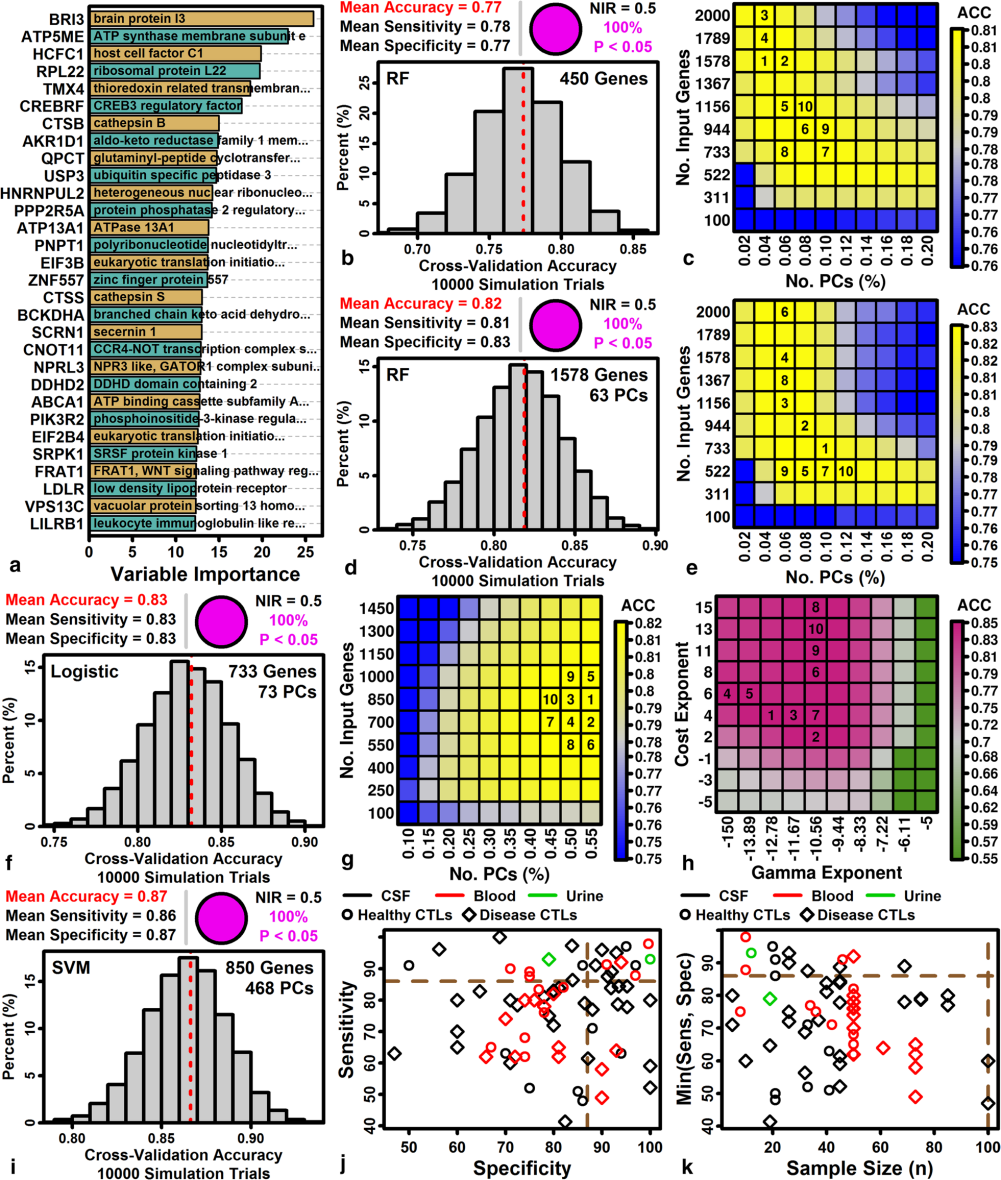
Classifiers for ALS diagnosis. **a** Random forest variable importance scores. Scores are shown for the top 30 genes. Tuning parameters yielding the highest out-of-bag (OOB) accuracy in preliminary trials were used (450 input genes, ntree = 400, mtry = 50). **b** Random forest prediction accuracy. The expression of 450 input genes was used as predictors. **c** Random forest PC analysis parameters. **d** Random forest prediction accuracy (with PC score predictors). **e** Logistic regression PC analysis parameters. **f** Logistic regression accuracy (PC predictors). **g** SVM PC analysis parameters. **h** SVM cost and gamma parameters. **i** SVM prediction accuracy. In **b**, **f** and **i**, histograms show the accuracy obtained across cross-validation trials (upper right: proportion of trials with accuracy significantly greater than non-information rate (NIR) of 50%, i.e., McNemar’s test). For each cross-validation trial, 592 subjects were used for training (296 ALS patients vs. 296 CTL/MIM subjects) and 200 subjects were used for testing (100 ALS patients vs. 100 CTL/MIM subjects). In **c**, **e**, **g** and **h**, cross-validation accuracy is shown for the analysis parameters as indicated on vertical and horizontal axes. For **c**, **e** and **g**, the number of PCs evaluated for each square is equal to the number of input genes (left axis) multiplied by the percentage of PCs (bottom axis). The 10 parameter combinations with highest accuracy are labeled (1 = highest accuracy). **j** Paired sensitivity and specificity estimates from 72 prior studies. Studies with disease control cohorts (diamonds) used patients with non-ALS neurological diseases, or the combination of healthy controls and patients with non-ALS neurological diseases. Dashed brown lines denote sensitivity and specificity estimates from the current study. **k** Min(Sens, Spec) versus sample size. For each pair of sensitivity and specificity estimates, the lower value is plotted (vertical axis) relative to the lower of the two ALS and CTL cohort sample sizes (horizontal axis). Dashed brown lines denote values from the current study

We next evaluated the diagnostic performance of multigene classifiers using random forest [78], logistic regression [79] and support vector machines (SVMs) [80]. Random forest variable importance scores were calculated to determine which genes, may be most important to prediction accuracy within multigene models (Fig. 6a. This highlighted genes such as brain protein I3 (*BRI3*), ATP synthase membrane subunit e (*ATP5ME*), and host cell factor C1 (*HCFC1*) as important multigenic model predictors (Fig. 6a). Cross-validation analysis showed that random forests using 450 input genes could predict ALS diagnosis with 77% accuracy on average (sensitivity: 78%, specificity: 77%) (Fig. 6b). However, using 63 PC scores as random forest predictors, rather than 450 input genes, improved prediction accuracy to 82% (sensitivity: 81%, specificity: 83%; Fig. 6c, d). Using logistic regression models with PC predictors further improved prediction accuracy to 83% (sensitivity: 83%, specificity 83%; Fig. 6e, f). Ultimately, our best results were obtained using SVMs, which could predict ALS diagnosis with 87% accuracy (sensitivity 86%, specificity 87%; Fig. 6g, h, I).

We reviewed the literature to identify 72 pairs of sensitivity and specificity estimates from previous fluid-based biomarker studies comparing ALS patients to controls (healthy controls or patients with non-ALS neurological diseases) (Additional file 19). The 86% sensitivity and 87% specificity obtained from SVM classification was better than 21 of 25 prior blood studies (Fig. 6j). Notably, however, most of the higher sensitivity/specificity estimates had been generated from small cohort studies (e.g., *n* ≤ 50 per group), and none of the prior blood biomarker studies performed with larger sample sizes (*n *≥ 60 per group) had achieved greater than 70% sensitivity and specificity (Fig. 6k).

### Copper chaperone for superoxide dismutase (*CCS*) is the gene with expression most strongly associated with ALS patient survival

Disease course varies among ALS patients with rapid progression in some and slow progression in others [92], or in rare cases, apparent disease reversal [93]. We evaluated 11,480 protein-coding genes to determine if expression was associated with survival in covariate-adjusted Cox PH models, and found that a larger-than-expected proportion of genes (1204/11,480, 10.5%) were survival-associated (P < 0 0.05). No single gene was significant at a stringent FDR threshold (FDR < 0.10), although 6 genes were survival-associated at a less conservative threshold (i.e., FDR < 0.30; Additional file 20A, C). The most significant survival-associated gene was copper chaperone for superoxide dismutase (*CCS*), which was expressed at higher levels in patients with improved survival (HR = 0.77; P = 1.84e−05; FDR = 0.14) (Additional file 20C). In contrast, genes encoding neurofilament proteins were not significantly associated with survival (*NEFH*: HR = 1.02, P = 0.77; *NEFL*: HR = 1.03, P = 0.59). Survival-associated genes with high expression linked to poor survival (P < 0.01) were associated with protein metabolism, macrophage differentiation, and platelet activation (Additional file 20B). Survival-associated genes with high expression linked to improved survival (P < 0.01) were associated with the mitochondrial respiratory chain, semaphorin-plexin signaling and nucleotide phosphorylation (Additional file 20D). Cross-validation analyses were performed to determine if individual genes could predict survival, but adding the expression of single genes only slightly improved performance of Cox PH models already including clinical covariates (age, site-of-onset, sex and cohort; mean *C* = 0.62 vs. mean *C* = 0.60–0.61; Additional file 20E–G). Covariate-adjusted accelerated failure time models [94], however, showed that predicted median survival differed 20–33% depending upon whether an individual gene had low (20th percentile) or high (80th percentile) expression (Additional file 20H–J).

Gene signature scores were calculated by averaging *Z*-score normalized expression for the 100 genes most specifically expressed by each immune cell type (or the 100 genes most highly elevated with high altitude stress). Higher RBC and MP scores were associated with improved survival in covariate-adjusted Cox PH models (RBC: HR = 0.71, FDR = 0.018; MP: HR = 0.41; FDR = 0.018) (Additional file 21A). Consistent with this, the 2-way combination RBC + MP predicted survival better than any other combination, and RBC + PL + MP was the best 3-way combination (likelihood ratio tests; Additional file 21B, C). The combination MC + NP, previously reported to predict disease progression [91], was a less effective combination for predicting survival (Additional file 21B). Notably, altitude stress signatures were not significantly associated with survival (HR = 1.06; P = 0.47; FDR = 0.79) (Additional file 21A). Signature scores or combinations thereof only slightly improved performance of predictive models in cross-validation analyses (Additional file 21D–F), although predicted median survival improved 27% in patients with favorable RBC + PL + MP scores (Additional file 21I).

### Heterogeneity of immune cell signatures suggests two patient subgroups with myeloid- and lymphoid-dominant expression patterns

**Fig. 7:**
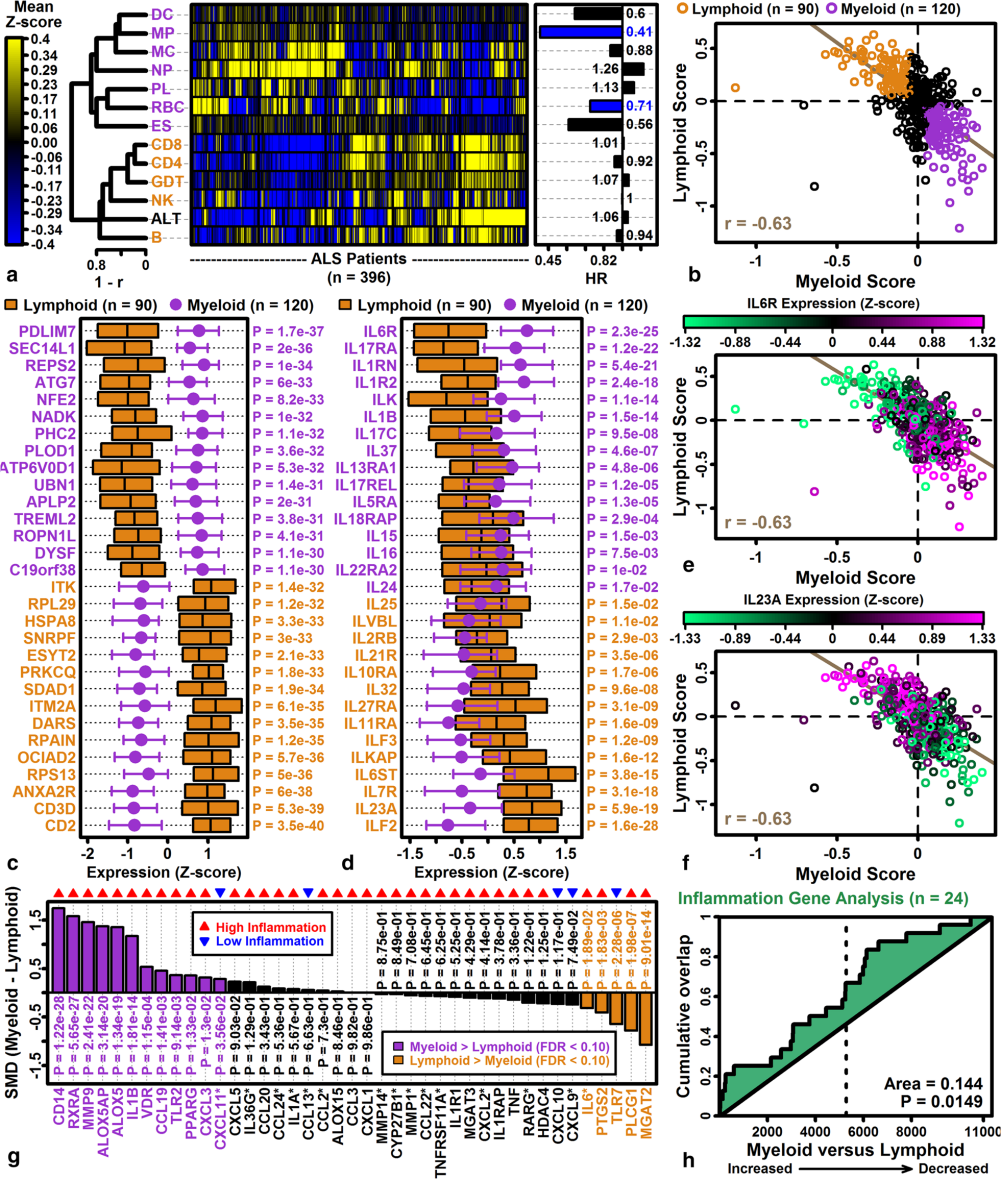
Myeloid and lymphoid ALS patient subgroups. **a** Cell type signature scores and hazard ratios (HRs). Scores were calculated by averaging Z-score normalized expression of the 100 genes most specifically expressed in each cell type (or the 100 genes most strongly induced with acute high altitude stress; see Methods). Heatmap rows and columns are clustered (rows: 1–correlation; columns: Euclidean distance; right: hazard ratios; covariates: age, sex, site of onset, and cohort; *n* = 396 patients). **b** Myeloid and lymphoid patient subgroups. Myeloid signatures were calculated for each patient by averaging DC, MP, MC, NP, PL, RBC and ES scores, and lymphoid signatures were calculated by averaging CD8, CD4, GDT, NK and B scores. Each symbol represents an individual patient (myeloid group: myeloid score > 0.05 and lymphoid score < − 0.05; lymphoid group: myeloid score < − 0.05 and lymphoid score > 0.05; green line: robust regression estimate). **c** Genes with strongest expression differences in myeloid and lymphoid group patients. **d** Interleukin genes. In **c**, **d**, boxes or error bars span the middle 50% of *Z*-score normalized expression values in each group (median: midline or circle). **e** *IL6R* expression. **f** *IL23A* expression. In **e**, **f**, symbol colors denote expression of *IL6R* and *IL23A*, respectively. **g** Inflammation-associated genes. The standardized mean difference (SMD) is shown for each gene (red triangle: genes with higher expression in “high inflammation” ALS patients; blue triangle: genes with higher expression in “low inflammation” ALS patients; see Figure 1A from Mizwicki et al. [42]). Gene labels with an asterisk (*) had detectable expression in fewer than 20% (< 42/210) of patients from the myeloid and lymphoid groups. **h** High inflammation gene GSEA analysis. Genes are ranked according to their expression difference in myeloid versus lymphoid patients (horizontal axis) and cumulative abundance of high inflammation genes is shown (vertical axis) (p-value: Wilcoxon rank sum test). The analysis excludes genes with low expression (*)

To deal with ALS patient heterogeneity, clinical trials have increasingly defined more homogeneous patient cohorts for enrollment [95]. This strategy can be biomarker-driven and is well-suited for immune-modulating drug candidates with specific targets (e.g., the anti-IL6R drug tocilzumab) [42, 43, 96]. Immune cell and high altitude stress signature scores varied among the 396 patients in our analysis (Fig. 7a). We discerned two patient groups, with one group having higher expression of genes expressed by myeloid-derived cells (NP, MC, DC, MP, PL, RBC, ES), and the other having higher expression of genes expressed by lymphoid-derived cells (CD8, CD4, GDT, B, NK) or high altitude response genes (Fig. 7a). Scores were averaged for each cell type group, yielding negatively correlated “myeloid” and “lymphoid” composite scores, respectively (Fig. 7b).

We identified 120 patients with high myeloid but low lymphoid scores, and conversely 90 patients with high lymphoid but low myeloid scores (Fig. 7b). Comparing these groups, T-cell marker gene (*CD2*, *CD3D*) and *IL23A* expression was increased in the lymphoid group (Fig. 7c, d, f). The myeloid group had higher expression of *IL6R*, IL-17 family genes (*IL17RA*, *IL17C*, *IL17REL*) and IL-1 family genes (*IL1RN*, *IL1R2*, *IL1B*) (Fig. 7d, e). We considered whether myeloid and lymphoid groups corresponded to “high inflammation” and “low inflammation” groups identified in a prior PBMC gene expression study [42]. Among genes previously shown to be elevated in “high inflammation” ALS patients, there was indeed a significant trend towards higher expression in our myeloid patient group (e.g., *CD14*, *RXRA*, *MMP9*; P = 0.015; Fig. 7g, h). However, consistent trends were not observed for all genes (e.g., *MGAT2*, *PLCG1*, *PTGS2*) including interleukin 6 (*IL6*) and interleukin 6 receptor (*IL6R*) (Fig. 7d, g).

Circulating monocytes may develop a pro-inflammatory phenotype in ALS patients with early stages of M1 macrophage polarization discernable from gene expression [97]. Signature scores were calculated based upon the expression of genes induced during macrophage polarization [76], which revealed a trend towards improved survival in patients with high M2 signatures (HR = 0.76; P = 0.39) and worse survival in patients with high M1 signatures (HR = 1.17), although neither association was significant (P = 0.53 and P = 0.39, respectively) (Additional file 22A). M1 and M2 signatures were weakly associated with myeloid and lymphoid scores (Additional file 22B, C), with both scores marginally elevated in myeloid group patients (M1: P = 0.056; M2: P = 0.161; Additional file 22D, E). Genes most strongly elevated during M1 or M2 polarization did not differ consistently between myeloid and lymphoid group patients (Additional file 22F, G).

### A 61 gene blood expression signature predicts post-diagnosis survival

**Fig. 8:**
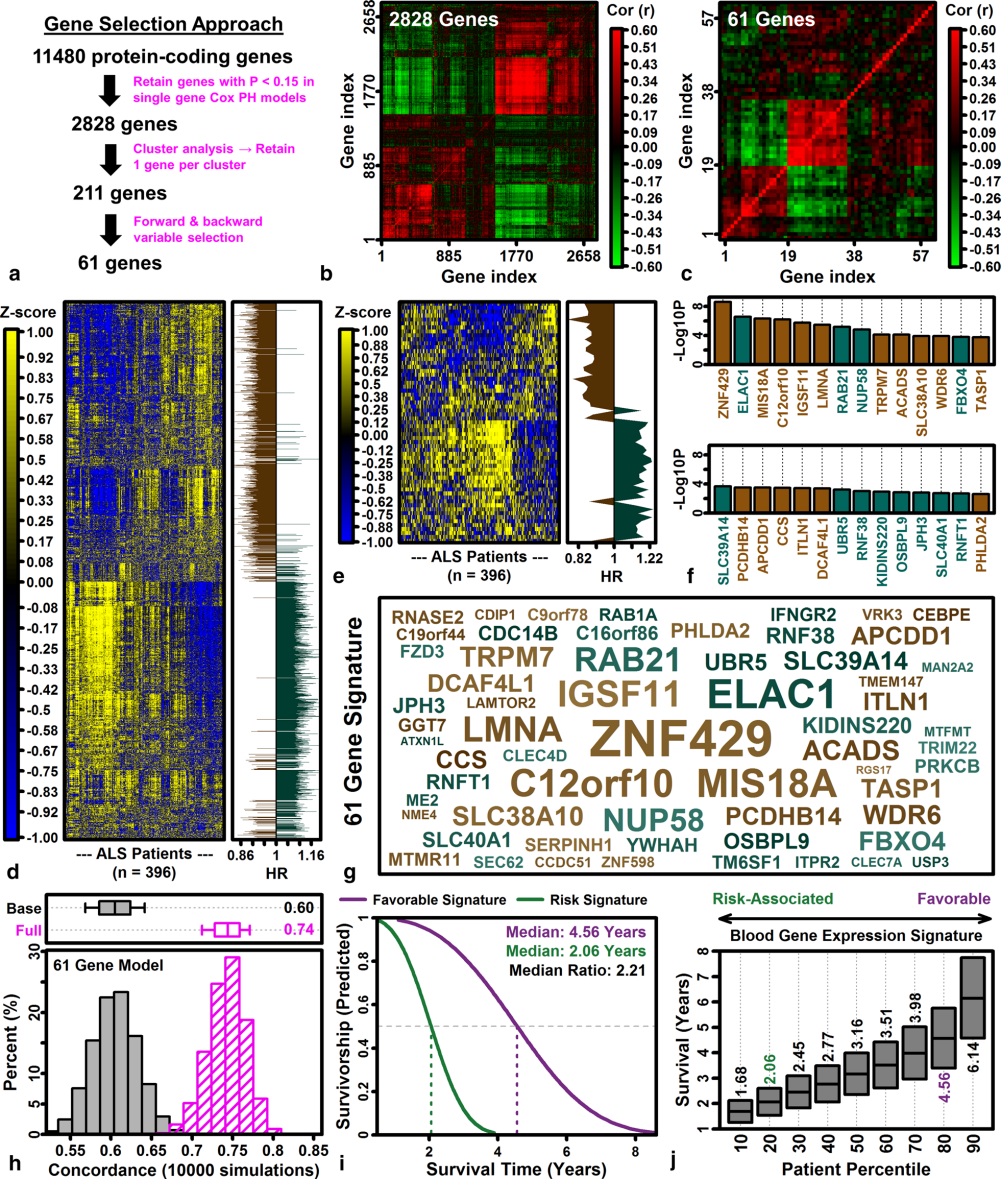
Blood gene expression signature to predict ALS patient survival. **a** Gene selection approach. 11,480 protein-coding genes expressed in at least 20% of ALS samples were filtered to include 2828 genes associated with survival in covariate-adjusted Cox PH models (P < 0.15; covariates: age of onset + site of onset + sex + batch). These genes were clustered into 211 groups with one representative gene chosen per group. Forward and backward variable selection was then used to obtain the final set of 61 genes. **b**, **c** Correlation matrices. Heatmaps show expression correlations among the filtered sets of 2828 and 61 genes. **d**–**f** Expression heatmaps and hazard ratios. Blue-yellow heatmaps show Z-score normalized expression (rows: genes; columns: patients; clustering: hierarchical based upon Euclidean distance) with hazard ratios (right) (**d**: 2828 gene set; **e**: 61 gene set). **f** Gene importance (likelihood ratio tests). P-values were calculated by comparing the full model log-likelihood (61 genes + covariates) with that of reduced models (60 genes + covariates) obtained by dropping each gene from the full model. The 28 genes with lowest likelihood ratio test p-values are shown (vertical axis: -log10-transformed p-values; red font: HR > 1.00; blue font: HR < 1.00). **g** Gene symbol cloud. The 61 signature genes are shown with size inversely proportional to likelihood ratio test p-values (red: HR > 1.00; blue: HR < 1.00). Gene symbol colors are proportional to hazard ratio estimates among from monogenic Cox PH models relating ALS patient survival to gene expression and covariates (brown: HR < 0, turquoise: HR > 0). **h** Cross-validation analysis of survival prediction accuracy (Cox PH model with 61 genes + covariates; training set: 296 ALS patients; testing set: 100 ALS patients). Concordance index distributions are shown for the base model (clinical covariates only) and full model (clinical covariates + 61 signature genes). Boxes (top) outline the middle 50% of outcomes (middle line: median; whiskers: 10th to 90th percentiles). **i** Predicted survivorship with favorable and risk gene expression signatures. The Cox PH model was fit and predicted 50% survival times were obtained for each patient (*n* = 396; clinical covariates + 61 gene signature). The favorable signature was obtained from the patient with predicted 50% survival time nearest to the 80th percentile, and the risk signature was obtained from the patient with predicted 50% survival time nearest to the 20th percentile. Estimated median survivorship for each group is shown (dashed lines) with the between-group ratio (upper-right). **j** Predicted survival times across the observed range of gene expression signatures. Patient percentiles were calculated from predicted survival times and boxes outline the middle 50% of survivorship times for each percentile (25th to 75th percentile; middle line: median)

Our analysis identified individual genes and signature scores associated with survival in Cox PH models, although these did not substantially improve prediction performance when evaluated by cross-validation (Additional file 19E–G; Additional file 20D–F). We therefore determined whether cross-validation performance could be improved by developing a more complex multigenic Cox PH model (Fig. 8). 2828 genes marginally associated with survival in single-gene Cox PH models were isolated (P < 0.15) and, as expected, expression of many of these genes was correlated (Fig. 8a, d). The 2828 genes were clustered (distance metric: |1–*r*|), yielding 211 non-overlapping gene groups (13.4 genes/group on average; range: 5–90 genes/group), from which we selected the 1 gene per group most strongly associated with survival in single-gene Cox PH models (Fig. 8b, e). These 211 genes were iteratively entered into a multigenic Cox PH regression model (i.e., forward and backward variable selection), leading to a final set of 61 genes (Fig. 8c, f, g; Additional file 23). Cross-validation showed that adding these 61 genes to Cox PH models with clinical covariates (age, site-of-onset, sex and cohort) substantially improved performance (mean *C* = 0.74 vs. mean *C* = 0.60; Fig. 8h). Predicted median survival differed 2-fold depending upon whether patients had a favorable or risk-associated expression pattern among the 61 predictor genes (Fig. 8i, j).

## Discussion

ALS primarily affects motor neurons in the brain and spinal cord, but peripheral blood analysis may provide a source for non-invasive biomarkers. This study provides a comprehensive analysis of blood gene expression in the largest expression profiling study of ALS patients performed to date [31]. Expression shifts in ALS blood frequently involved genes specifically expressed by certain immune cell types (e.g., neutrophils), and we show that expression of such genes can be used to identify patient subgroups (Fig. 7). The value of blood expression markers in ALS, however, is not limited to inflammation monitoring alone. Our results suggest that blood gene expression may additionally provide insights into sub-clinical hypoxia and respiratory function [98, 99], as well as pathogenetic mechanisms suggested by GWAS findings [88]. Our findings support the potential of blood-derived expression markers as tools for prediction of ALS diagnosis and patient survival. Sensitivity and specificity estimates from this study were better than those reported previously in large cohort studies of blood proteins, and were competitive with estimates from CSF protein studies (Fig. 6j, k). At present, no major technical barriers exist that would prevent investigators from exploiting blood gene expression biomarkers in ALS more fully. It is nearly certain that further progress can be achieved through analysis of larger patient cohorts and leveraging of high-throughput sequencing technology. Ultimately, we expect that the most useful clinical tools will emerge from predictive “hybrid models” that combine blood-derived biomarkers with those obtained from other biofluids (e.g., CSF, urine and saliva) and/or validated clinical measures (e.g., FVC and the revised ALS functional rating scale (ALSFRS-R)) [44].

Transcriptomic datasets can be analyzed from multiple perspectives and the purpose of online data repositories (e.g., GEO) is to facilitate re-analysis of data using alternative methods [74]. This study focused on data originally generated by van Rheenen et al. [31] and demonstrates challenges that arise when combining data across batches and microarray platforms [100]. ALS DEGs identified in our study overlapped significantly with those from van Rheenen et al. [31], but only 30% of DEGs identified in our study were identified as differentially expressed by van Rheenen et al. [31]. This lack of overlap may be explained by alternative batch correction and differential expression analysis approaches. We used a different batch correction algorithm (ComBat) [35] and avoided simultaneous correction for batch and platform-specific variability, with DEGs identified by “late stage” meta-analysis [46] to ensure DEGs exhibited consistent patterns in both cohorts. Outlier removal may also be an important difference between analyses. The analysis by van Rheenen et al. [31] identified and removed 87 samples as outliers, whereas our analysis removed only 1 outlying sample. The lack of prominent outliers in our analysis may reflect improved resolution of technical variability using the ComBat algorithm [101]. Despite these differences, some key trends were similar between our analysis and that of van Rheenen et al. [31]. For instance, ALS-increased DEGs we identified frequently encoded RNA-binding proteins and ribosome components (Additional file 11), consistent with findings from van Rheenen et al. [31]. As noted by van Rheenen [31], genes linked to RNA processing have been identified in ALS genetic association studies (e.g., *TARDBP*, *FUS*) and there is evidence for RNA-mediated toxicity in cells harboring *C9orf72* repeat expansions [102]. Both our study and that of van Rheenen et al. [31] therefore appear to have identified dysregulation of RNA processing genes in the ALS transcriptome, which has increasingly been viewed as a component of ALS pathogenesis [102].

An immunological component to ALS pathophysiology has also been recognized [19, 103], and prior studies have identified alterations in immune cell abundance and activity in ALS patients [104]. Blood gene expression is partly determined by the fractional abundance of constituent immune cells, and as such in silico approaches have been developed to “deconvolute” aggregate expression signals to allow inferences regarding fractional cell abundance [70–73]. Using this approach, the strongest trend we identified was over-representation of neutrophil-specific genes among ALS-increased DEGs (Fig. 3a). This result is consistent with neutrophilia in ALS patients as previously demonstrated in smaller patient cohorts [91, 105–108] and confirmed by flow cytometry [91]. In ALS, neutrophilia and associated low-grade inflammation [107] may be reactive and secondary to motor neuron degeneration, although some evidence supports a direct and causal role in disease pathogenesis [109]. Recently, for example, heavy neutrophil infiltration surrounding motor axons was reported in SOD1G93A rats, and neuron degeneration and myofibril loss in this model were prevented by the tyrosine kinase inhibitor masitinib [109]. Notably, increased expression of neutrophil-specific genes in ALS patients was not mirrored in patients with ALS mimic diseases (Additional file 18B), suggesting that neutrophilia may be an ALS-specific phenotype and potentially useful for excluding differential diagnoses. In prior work, degree of neutrophilia has been correlated with functional decline as measured by ALSFRS-R [91, 104]. This trend was weakly supported by our analysis, since high neutrophil signature scores were marginally associated with decreased survival (HR = 1.26; P = 0.069; Fig. 7a).

ALS patients in this study had lower expression of genes specifically expressed by RBC lineage cell types (i.e., erythroblasts and reticulocytes; Fig. 3d). This trend was less robust but bolstered by associations between ALS-decreased DEGs with anemia and blood diseases (Additional file 13F). One possible interpretation is that ALS patients develop reduced RBC numbers, potentially leading to sub-clinical anemia at certain stages of the disease course. Early studies have demonstrated increased mechanical fragility of erythrocytes from ALS patients, with enhanced sensitivity to haemolysis following lead exposure [110, 111]. ALS patient erythrocytes were also reported to have greater sensitivity to oxidative stress along with reduced activity of antioxidant defense enzymes such as glutathione peroxidase [112–114]. Other unique RBC phenotypes have been described in ALS patients as well, such as increased erythrocyte deformability and acetylcholinesterase activity [115], accumulation of altered aspartyl residues [116], and decreased nitric oxide efflux and intraerythrocytic nitrite [115]. In this study, ALS patients not only had reduced expression of RBC lineage-specific genes in blood, but those with higher expression of such genes had improved survival (HR = 0.71; P = 0.002; Fig. 7a). In agreement with this finding, a retrospective cohort study of 1.8 million young men enlisted in the Swedish military recently showed that a 1% increase in erythrocyte volume fraction (EVF) was associated with 4% decreased risk of later developing ALS (P = 0.05) [117]. In view of these results, it is interesting to note that blood-CSF barrier defects have been documented in ALS patients and rodent models [13], which may provide a route for erythrocyte extravasation into spinal cord regions with high motor neuron density [13], where deposition of hemoglobin may be toxic [118]. Our findings therefore augment evidence for unique RBC phenotypes in ALS patients, which may be useful as disease biomarkers [115] or potentially play a direct role in disease onset and/or progression [13, 118].

The idea that ALS patients can be divided into “high” and “low” inflammatory groups has previously been suggested based upon gene expression analysis of PBMCs from small patient cohorts (i.e., *n* ≤ 9 patients) [42, 43]. Based upon our large cohort analysis (*n* = 396 patients), using whole blood immune cell expression signatures, we identified two patient subgroups with myeloid- and lymphoid-dominant expression patterns, respectively (Fig. 7b). These groups approximate the “high” versus “low” inflammation groups suggested previously (Fig. 7g, h) [42], although this distinction may not be fully applicable since certain cytokine mRNAs were elevated in both groups (Fig. 7d). The significance of these findings is that blood gene expression signatures may provide tools to screen patients prior to clinical trial enrollment. In recent years, an objective in clinical trial design has been to enroll more homogenous ALS patient groups, based upon biomarkers and/or measures that reflect disease progression or physiological function [95, 119]. For example, a recent phase 2 study of the macrophage activation inhibitor NP001 [120] only enrolled ALS patients with plasma C-reactive protein (CRP) concentration greater than or equal to 0.113 mg/dL (NCT02794857) [96]. Likewise, a phase 2 study of the IL-6 receptor antibody tocilizumab enrolled ALS patients with a “high inflammatory profile” based upon PBMC gene expression analysis (NCT02469896) [42, 121]. In our study, *IL6* expression was slightly lower in the myeloid group (P = 1.89e−02), but expression of *IL6R* was elevated with greater statistical significance (P = 2.3e−25), suggesting the hypothesis that myeloid group patients would respond more favorably to tocilizumab. Myeloid and lymphoid groups also differed in the expression of mRNAs encoding IL-1, IL-17 and IL-23 pathway components (Fig. 7d), which have been less studied in ALS but can be targeted using biologic therapies now available [122, 123]. Myeloid and lymphoid subgroups identified here may thus represent sub-cohorts enriched for patients more likely to respond to immunomodulatory agents targeting IL-1, IL-6, IL-17 or IL-23.

Blood gene expression has most often been incorporated into ALS clinical trials as an inflammation biomarker [121], but our results suggest a broader role that extends beyond inflammation monitoring alone. Notably, gene expression shifts in ALS blood showed striking correspondence with those observed during acute high altitude stress (Fig. 4). Respiratory decline is expected with ALS progression and may have physiological effects at early disease stages, prior to the onset of overt dyspnea or measureable FVC decline [98, 99]. A recent study, for example, documented poor sleep quality in most ALS patients (63%) at the time of diagnosis, likely due to nocturnal hypoventilation and respiratory muscle weakness [124]. In some ways, this respiratory dysfunction may be mimicked by the reduced oxygen pressures associated with high altitude stress, which are responsible for symptoms of “acute mountain sickness” (e.g., headache, nausea, pulmonary hypertension, cerebral edema) [125, 126]. Similar to ALS respiratory decline, moreover, physiological responses to high altitude are broad and may involve release of hypoxia inducible factor (HIF) and activation of renal compensatory mechanisms [125–127]. Our results therefore suggest the novel possibility that peripheral blood gene expression can provide a sensitive indicator of respiratory dysfunction at early and late stages of the disease, which may be of clinical value since standard tests such as vital capacity may not be fully indicative of diaphragm atrophy or function [128]. Interestingly, altitude stress scores among patients were not themselves associated with survival in covariate-adjusted models (HR = 1.06; P = 0.47; Additional file 21A). This may indicate that the high altitude blood signature is not a “marker” of severe respiratory distress, emergent at later disease stages, but may instead reflect degrees of sub-clinical hypoxia and respiratory muscle weakness, which can be present even at the time of diagnosis [124].

Early ALS symptoms may go unnoticed or mimic other diseases, which can lead to diagnostic delay and compromised quality of care [129–131]. One study reported a median time to diagnosis of 11 months with one-third of ALS patients having initially been misdiagnosed [130]. Such delays hinder care plan formulation, slow initiation of treatments that might improve survival or quality of life, reduce time available for clinical trial enrollment, and add to the frustration and expense of ALS patients and caregivers [129–131]. Diagnostic delays are in part due to the complex process for ALS diagnosis, which requires full exclusion of other conditions, requiring multiple referrals and rounds of testing [132]. Development of an accurate biomarker test, however, would simplify the process and likely lead to earlier diagnosis [12]. In our analyses, mRNAs encoding proteins previously identified as possible blood biomarkers (e.g., pNFH and NFL) [6, 90] weakly discriminated ALS from CTL and MIM patients (Additional file 16A–C). We identified individual genes that could diagnose ALS patients with 62–63% accuracy (Additional file 17H, I), but ultimately best results were obtained using an SVM classifier with PC scores as predictors, which yielded 87% accuracy (sensitivity: 86%, specificity: 87%) (Fig. 6i). These sensitivity and specificity estimates are better than those previously reported for blood biomarkers in large cohort studies (≥ 60 patients per group) and comparable to CSF protein biomarkers (Fig. 6k). Our estimates also compare well to those reported in studies using other technologies such as transcranial magnetic stimulation (sensitivity: 73%, specificity: 81%) [133], conventional MRI (sensitivity: 48%, specificity: 76%) [134], and diffusion tensor imaging (sensitivity: 65%, specificity: 67%) [135]. Our findings thus support the idea that blood-derived mRNA biomarkers can be superior to some diagnostic approaches and competitive with CSF protein analysis [11, 136–138].

Exosomes are extracellular vesicles ranging in size from 40 to 100 nm, which are generated from endosome membranes with cargos consisting of proteins and nucleic acid species (e.g., mRNAs and microRNAs) [64]. Because of their unique role in intercellular transport and communication, and their ability to cross the blood-CSF barrier [139], exosomes have drawn interest as candidate neurodegenerative disease biomarkers [39, 140]. ALS-increased DEGs had higher expression in blood exosomes from normal subjects (Additional file 16D, E), and exosome-associated mRNAs [66, 67] were disproportionately increased in ALS blood samples (Additional file 16B, C). ALS-increased DEGs with highest blood exosome expression frequently encoded ribosomal subunits and other translation-associated proteins (Additional file 16G, H). These observations connect well with those from a recent study demonstrating an increased diameter of exosomes extracted from ALS patient plasma, which further showed that such exosomes have increased abundance of disease-related proteins (e.g., SOD1, TDP-43 and FUS) [38]. The ability to quantify exosome mRNA strengthens the biomarker value of ALS patient blood samples, particularly since exosome mRNA may originate from motor neurons and transit the blood-CSF barrier to enter peripheral circulation [139]. Along these lines, it is important to note that our study analyzed oligonucleotide microarray data, which compared to RNA-seq has a more limited dynamic range and less sensitivity for quantifying expression of low-abundance transcripts [141]. Since extracellular vesicles passing into blood from CSF may be present in low abundance, RT-PCR or RNA-seq would likely provide improved quantification [141], potentially leading to biomarkers with improved diagnostic accuracy. To our knowledge, a comprehensive comparison of exosome mRNAs in blood of ALS patients and controls has not been performed, but our findings, combined with other recent data [38], provide rationale for such work using a deep sequencing approach.

Superoxide dismutase 1 (*SOD1*) mutations were the first to be associated with ALS [1], since replicated in multiple cohorts [142], and may contribute to disease by causing SOD1 destabilization and mitochondrial accumulation [143, 144]. Among 11,480 protein-coding genes evaluated, expression of copper chaperone for superoxide dismutase (*CCS*) was most strongly associated with survival (P = 1.84e−05; FDR = 0.14), with increased expression favoring improved survival (HR = 0.77) (Additional file 20C). *CCS* encodes a copper chaperone for SOD1 and interacts with SOD1 to facilitate copper uptake, promote structural maturation, and control intracellular localization [145]. Consistent with this, CCS and SOD1 proteins are co-localized in human cortical pyramidal neurons, cerebellar Purkinje cells, and spinal cord motor neurons [146]. In principle, CCS-SOD1 interactions may enhance SOD1 stability and possibly protect against misfolding and aggregation of the mutated variant [147]. Paradoxically, however, when *CCS* is overexpressed in G93A-SOD1 mice, *CCS*-G93A-SOD1 transgenics die 8 times more quickly with increased G93A-SOD1 mitochondrial localization [148]. This seems difficult to reconcile with improved survival in ALS patients having elevated blood *CCS* mRNA levels (Additional file 20C). Notably, however, SOD1 aggregates are a key ALS hallmark but are absent in spinal cords from CCS/G93-SOD1 mice [149], and mortality in these mice may be explained entirely by copper deficiency during the first 10 days of life [150], in some ways resembling Menke’s disease better than adult-onset ALS [151]. Further investigation may therefore be needed to understand the significance of *CCS* in ALS pathophysiology apart from the copper deficiency phenotype observed in *CCS*-G93-SOD1 transgenic mice.

The ability to predict ALS patient survival has been an ongoing challenge [152–154], and biomarkers for this purpose would be especially valuable as early indicators of efficacy in ALS clinical trials [7]. Although *CCS* and other individual genes (e.g., *JAK1*, *CEBPE, KEL*) were marginally associated with survival (FDR < 0.30), such genes, taken individually, only modestly improved survival forecasts when combined with baseline clinical data in Cox PH models (Additional file 20). We therefore developed a multivariate model with 61 predictor genes and showed that adding these genes to a Cox PH model with clinical data substantially improved survival forecasts, yielding an overall concordance index of 0.74 (Fig. 8h). In comparison, a recent study reported a concordance index of 0.78 using prediction models based upon 8 variables, including 2 used in our analyses (age at onset and site of onset) and 6 others not included in our analyses (FVC, definite versus probable or possible ALS, diagnostic delay, progression rate, frontotemporal dementia, and presence of a *C9orf72* repeat expansion) [92]. Including these clinical variables along with others in our model would likely have improved performance, although new datasets will be needed to confirm this expectation. Important survival-associated genes in our signature include *ZNF429*, *ELAC1*, *MIS18A* (Fig. 8f, g), although given our heuristic variable selection approach, we do not expect that the 61 genes are necessarily optimal and potentially other gene sets could be identified with similar predictive performance. Our findings, however, in contrast to an earlier report [31], provide proof-of-principle to support the use of blood-derived expression biomarkers as predictors of ALS patient survival. Although further validation of our signature is needed, development of a prognostic blood biomarker panel would alter the landscape of tools now available to ALS researchers and clinicians [7, 152–154].

## Conclusions

Development of fluid-based ALS biomarkers remains a longstanding research challenge, and blood biomarkers would be especially useful since blood draws can be performed quickly and easily [10, 12]. Prior gene expression analyses of ALS blood samples have been performed [28–30] but have been under-replicated given the heterogeneous nature of this disease [33] (Additional file 1). This study therefore analyzed data from two large patient cohorts with a combined total of 396 ALS patients, 75 patients with ALS-like disease, and 645 control subjects [31]. We identify a robust blood transcriptome signature consistent with neutrophilia, altered translation and hypoxia resembling an acute altitude stress response, and show that ALS-increased DEGs have high exosome expression and overlap significantly with genes near ALS GWAS loci. These results suggest that ALS blood gene expression may provide a window into multiple aspects of the disease beyond inflammation, including respiratory dysfunction [98, 99, 107] and pathogenetic mechanisms underlying disease onset and progression [88]. We identified considerable immunological heterogeneity among ALS patients, leading to the identification of two patient groups with distinctive cytokine profiles and differing in the expression of genes specifically expressed by cells from the myeloid and lymphoid lineages, respectively. We show that blood-derived biomarkers can be incorporated into SVM models to predict ALS diagnosis with 87% accuracy (sensitivity: 86%, specificity: 87%), and develop a 61 gene signature that improved survival forecasts substantially when added to Cox PH regression models with baseline clinical data (74% concordance). These findings provide rationale for further studies of blood gene expression biomarkers in ALS research, which may be used for screening patients prior to clinical trial enrollment, informing ALS diagnosis, and forecasting disease trajectory.

## Additional files

**Additional file 1.** Whole-genome expression analyses of ALS and CTL patient blood samples. Table columns list the first author with publication year, gene expression profiling platform, PubMed identifier, GEO series accession (if available), cell types analyzed, and the number of samples used for expression profiling. Only studies of blood-derived cell types are included. Full references are listed below the table with additional relevant information (see footnotes).

**Additional file 2.** Description of patient cohorts. (A) Number of subjects per group. (B) Male and female frequencies. (C) Average age per group. (D) Site of onset. (E) C9orf72 status. (F) ALS patient survival (*n* = 397). The Kaplan–Meier (KM) survival estimate is shown with 95% confidence intervals (upper right: survivorship quantiles). (G) MIM cohort diagnoses (*n* = 75). The frequency of cases is shown for each condition (CBD: corticobasal degeneration; CIPD: chronic inflammatory demyelinating polyneuropathy; FOSMN: facial onset sensory and motor neuronopathy; HSP: hereditary spastic paraplegia; MMN: multifocal motor neuropathy; SMA: spinal muscular atrophy).

**Additional file 3.** Microarray normalization and batch adjustment (GSE112676). (A) Median signal intensity histogram. (B) Interquartile range (IQR) histogram. (C) Number of protein-coding genes with detectable expression histogram. In (A–C), histograms show the distribution among 741 samples, and the median and range is given (top margin). (D) Median intensity sample index plot. (E) Interquartile range sample index plot. (F) Number of protein-coding genes with detectable expression sample index plot. In (D–F), the horizontal axis corresponds to the ordering of samples as listed in the GEO entry (GSM3076582–GSM3078510). (G) PC plot (2 dimensions). (H) PC plot (3 dimensions). (I) PC 1 sample index plot. (J) PC plot (2 dimensions) after SVA batch adjustment. (K) PC plot (3 dimensions) after SVA batch adjustment. (L) PC 1 sample index plot after SVA batch adjustment. (M) PC plot (2 dimensions) after ComBat batch adjustment. (N) PC plot (3 dimensions) after ComBat batch adjustment. (O) PC 1 sample index plot after ComBat batch adjustment. In (M)–(O), the sample designated as an outlier and removed from analyses is indicated (GSM3077426).

**Additional file 4.** Microarray normalization and batch adjustment (GSE112680). (A) Median signal intensity histogram. (B) Interquartile range (IQR) histogram. (C) Number of protein-coding genes with detectable expression histogram. In (A–C), histograms show the distribution among 376 samples, and the median and range is given (top margin). (D) Median intensity sample index plot. (E) Interquartile range sample index plot. (F) Number of protein-coding genes with detectable expression sample index plot. In (D–F), the horizontal axis corresponds to the ordering of samples as listed in the GEO entry (GSM3076582–GSM3078510). (G) PC plot (2 dimensions). (H) PC plot (3 dimensions). (I) PC 1 sample index plot. (J) PC plot (2 dimensions) after SVA batch adjustment. (K) PC plot (3 dimensions) after SVA batch adjustment. (L) PC 1 sample index plot after SVA batch adjustment. (M) PC plot (2 dimensions) after ComBat batch adjustment. (N) PC plot (3 dimensions) after ComBat batch adjustment. (O) PC 1 sample index plot after ComBat batch adjustment.

**Additional file 5.** Differential expression plots (GSE112676). (A) P-value distribution (males + females). (B) P-value distribution (male only analysis). (C) P-value distribution (female only analysis). In (A)–(C), the distribution of raw p-values among protein-coding genes is shown. The inset (upper right) shows the distribution of p-values less than 0.05. (D) Volcano plot (males + females). (E) Volcano plot (male only analysis). (F) Volcano plot (female only analysis). (G) MA plot (males + females). (H) MA plot (males only analysis). (I) MA plot (female only analysis). In (D)–(I), the number of increased (red) and decreased (blue) DEGs is shown in the upper margin (FDR < 0.10 with FC > 1.10 or FC < 0.91).

**Additional file 6.** Differential expression plots (GSE112680). (A) P-value distribution (males + females). (B) P-value distribution (male only analysis). (C) P-value distribution (female only analysis). In (A)–(C), the distribution of raw p-values among protein-coding genes is shown. The inset (upper right) shows the distribution of p-values less than 0.05. (D) Volcano plot (males + females). (E) Volcano plot (male only analysis). (F) Volcano plot (female only analysis). (G) MA plot (males + females). (H) MA plot (males only analysis). (I) MA plot (female only analysis). In (D)–(I), the number of increased (red) and decreased (blue) DEGs is shown in the upper margin (FDR < 0.10 with FC > 1.10 or FC < 0.91).

**Additional file 7.** Meta-analysis differential expression plots. (A) FC scatterplot (GSE112676 versus GSE112680; 9822 protein-coding genes). The yellow ellipse outlines the middle 50% of genes closest to the bivariate median (Mahalanobis distance). The spearman rank correlation coefficient is shown (upper-left). The percentage of genes in each quadrant is indicated (top margin; red font: P < 0.05, Fisher’s exact test). The color-coded vertical bar (right margin) reflects the proportion of genes in each quadrant. (B) P-value distribution (inset: p-values less than 0.05). (C) Volcano plot. (D) MA plot. In (C) and (D), the number of increased (red) and decreased (blue) DEGs is shown in the upper margin (FDR < 0.10 with FC > 1.10 or FC < 0.91).

**Additional file 8.** Gene expression responses to riluzole (GSE96653). (A) Riluzole chemical structure. (B) Median intensity prior to quantile normalization. (C) Signal IQR prior to quantile normalization. (D) Number of protein-coding genes with detectable expression in each sample (P < 0.05). (E) Cluster analysis. The 6 samples were clustered hierarchically based upon the Euclidean distance with average linkage. (F) PC plot. The 6 samples are plotted with respect to the first 2 PC axes. (G) Differential expression analysis raw p-value distribution (RZE vs. CTL; 11,868 protein-coding genes). (H) Volcano plot. (I) MA plot. In (H) and (I), the number of increased (red) and decreased (blue) DEGs is shown in the upper margin (FDR < 0.10 with FC > 1.10 or FC < 0.91).

**Additional file 9.** Signature gene expression in 12 cell types. (A) Erythroid lineage. (B) Platelets. (C) CD4 + T cells. (D) NK cells. (E) CD8 + T cells. (F) B cells. (G) Macrophages. (H) Monocytes. (I) Dendritic cells. (J) Neutrophils. (K) Eosinophils. (L) Gamma-delta T cells. In (A)–(L), expression is shown for the 100 signature genes identified for each cell type. Boxes outline average expression among samples for the middle 50% of signature genes (midline: median expression). Median expression for each cell type is listed in the top margin. Relative gene expression is normalized to an average value of 1 across the 12 cell types.

**Additional file 10.** DEG lists and annotation-based enrichment analyses. (A) List of 752 ALS-increased DEGs (FDR < 0.10, FC > 1.10). (B) List of 764 ALS-decreased DEGs (FDR < 0.10, FC < 0.909). (C) GO BP increased DEGs. (D) GO BP decreased DEGs. (E) GO BP increased DEGs + decreased DEGs. (F) GO CC increased DEGs. (G) GO CC decreased DEGs. (H) GO CC increased DEGs + decreased DEGs. (I) GO MF increased DEGs. (J) GO MF decreased DEGs. (K) GO MF increased DEGs + decreased DEGs. (L) KEGG increased DEGs. (M) KEGG decreased DEGs. (N) KEGG increased DEGs + decreased DEGs. (O) REACTOME increased DEGs. (P) REACTOME decreased DEGs. (Q) REACTOME increased DEGs + decreased DEGs. (R) DO increased DEGs. (S) DO decreased DEGs. (T) DO increased DEGs + decreased DEGs. (U) MSigDB increased DEGs. (V) MSigDB decreased DEGs. (W) MSigDB increased DEGs + decreased DEGs. (X) Pathway commons increased DEGs. (Y) Pathway commons decreased DEGs. (Z) Pathway commons increased DEGs + decreased DEGs. In (C)–(Z), spreadsheets list annotation terms most significantly enriched with respect to ALS-increased DEGs, ALS-decreased DEGs, or the combined set of ALS-increased + ALS-decreased DEGs (p-values: conditional hypergeometric test or Fisher’s exact test). The final column in each spreadsheet lists DEG symbols associated with each annotation term.

**Additional file 11.** Gene annotations enriched among ALS-increased DEGs. (A) Gene Ontology biological processes. (B) Gene Ontology cell components. (C) Gene Ontology molecular functions. (D) Kyoto Encyclopedia of Genes and Genomes (KEGG) pathways. (E) Reactome pathway database. (F) Disease Ontology. (G) Molecular signatures database (MSigDB). (H) Pathway commons. In (A)–(H), enrichment was evaluated with respect to 580 ALS-increased DEGs (FC > 1.10 with FDR < 0.10). The 12 most significantly over-represented annotations are listed for each analysis (Fisher’s exact test or conditional hypergeometric test). The number of genes associated with each annotation is listed in parentheses, and exemplar ALS-increased genes associated with each annotation are shown. In part (G), MSigDB annotations correspond to gene sets generated from the comparison of two sample groups (*X*|*Y*), with genes in each set having higher expression in group *X* as compared to group *Y.*

**Additional file 12.** ALS-increased DEG overlap with genes identified by Saris et al. [32]. (A) FC distribution for 793 genes previously identified as elevated in ALS patient whole blood (red symbols: ALS-increased DEGs, FDR < 0.10, FC > 1.10; blue symbols: ALS-decreased DEGs, FDR < 0.10, FC < 0.91). The Venn diagram (top-left) shows the overlap between ALS-increased genes from both studies (p-value: Fisher’s exact test). (B) GSEA analysis. Genes are ranked based upon their expression difference in ALS vs. CTL subjects from the current study (horizontal axis), and cumulative overlap with ALS-increased genes from Saris et al. [32] is shown (vertical axis) (p-value, lower right, Wilcoxon rank sum test). (C) Top ALS-increased DEGs ranked by FC (red font: ALS-associated genes; *riluzole-increased DEG, FDR < 0.10). Meta-FC estimates were obtained using a random effects meta-analysis model to integrate results from all 3 blood studies (i.e., GSE112676, GSE112680 and Saris et al. [32]). (D) Gene Ontology biological processes. (E) Kyoto Encyclopedia of Genes and Genomes (KEGG) pathways. In (D) and (E), enrichment was evaluated with respect to 572 ALS-increased DEGs (FC > 0.90 with FDR < 0.10). The 12 most significantly over-represented annotations are listed for each analysis (Fisher’s exact test or conditional hypergeometric test). The number of genes associated with each annotation is listed in parentheses, and exemplar ALS-increased genes associated with each annotation are shown.

**Additional file 13.** Gene annotations enriched among ALS-decreased DEGs. (A) Gene Ontology biological processes. (B) Gene Ontology cell components. (C) Gene Ontology molecular functions. (D) Kyoto Encyclopedia of Genes and Genomes (KEGG) pathways. (E) Reactome pathway database. (F) Disease Ontology. (G) Molecular signatures database (MSigDB). (H) Pathway commons. In (A)–(H), enrichment was evaluated with respect to 666 ALS-decreased DEGs (FC < 0.91 with FDR < 0.10). The 12 most significantly over-represented annotations are listed for each analysis (Fisher’s exact test or conditional hypergeometric test). The number of genes associated with each annotation is listed in parentheses, and exemplar ALS-decreased genes associated with each annotation are shown. In part (G), MSigDB annotations correspond to gene sets generated from the comparison of two sample groups (*X*|*Y*), with genes in each set having higher expression in group *X* as compared to group *Y.*

**Additional file 14.** ALS-decreased DEG overlap with genes identified by Saris et al. [32]. (A) FC distribution for 791 genes previously identified as decreased in ALS patient whole blood (red symbols: ALS-increased DEGs, FDR < 0.10, FC > 1.10; blue symbols: ALS-decreased DEGs, FDR < 0.10, FC < 0.91). The Venn diagram (top-left) shows the overlap between ALS-decreased genes from both studies (p-value: Fisher’s exact test). (B) GSEA analysis. Genes are ranked based upon their expression difference in ALS vs. CTL subjects from the current study (horizontal axis), and cumulative overlap with ALS-decreased genes from Saris et al. [32] is shown (vertical axis) (p-value, lower right, Wilcoxon rank sum test). (C) Top ALS-decreased DEGs ranked by FC (blue font: ALS-associated genes; *riluzole-decreased DEG, FDR < 0.10). Meta-FC estimates were obtained using a random effects meta-analysis model to integrate results from all 3 blood studies (i.e., GSE112676, GSE112680 and Saris et al. [32]. (D) Gene Ontology biological processes. (E) Kyoto Encyclopedia of Genes and Genomes (KEGG) pathways. In (D) and (E), enrichment was evaluated with respect to 441 ALS-decreased DEGs (FC < 0.91 with FDR < 0.10). The 12 most significantly over-represented annotations are listed for each analysis (Fisher’s exact test or conditional hypergeometric test). The number of genes associated with each annotation is listed in parentheses, and exemplar ALS-decreased genes associated with each annotation are shown.

**Additional file 15.** Cell type enrichment (ImSig algorithm). (A) Cell type ranking. Grey boxes outline the middle 50% of ImSig cell type scores for CTL subjects (*n* = 645; middle line: median). Magenta bars span the middle 50% of ImSig scores for ALS patients (*n* = 396; circle: median). P-values obtained from the test of an ALS vs. CTL score difference are listed (right margin; two-tailed t-test; red: FDR < 0.05 with ALS > CTL; blue: FDR < 0.05 with CTL > ALS). Cell type scores (horizontal axis) were normalized using a Z-score transformation and combined across the two cohorts (GSE112676 and GSE112680). (B) Neutrophil score histograms. (C) Translation score histograms. In (B) and (C), cell type scores from each cohort (GSE112676 and GSE112680) were normalized using the *Z*-score transformation and expression values from the two cohorts were combined. Boxplots (top margin) outline the middle 50% of expression values in each group (whiskers: 10th to 90th percentile; p-values: two-tailed t-test). (D) Top 40 neutrophil signature genes with lowest p-value (ALS vs. CTL). (E) Neutrophil signature gene GSEA analysis. (F) Top 40 translation signature genes with lowest p-value (ALS vs. CTL). (G) Translation signature GSEA analysis. (H) Top 40 macrophage signature genes with lowest p-value (ALS vs. CTL). (I) Macrophage signature GSEA analysis. In (D), (F) and (H), red bars/font indicates ALS-increased DEGs (FDR < 0.10 with FC > 1.10) and blue bars/font indicates ALS-decreased DEGs (FDR < 0.10 with FC < 0.91). The pie chart (upper right) indicates the proportion of genes not significantly altered in the ALS vs. CTL comparison (black), ALS-increased DEGs (red) and ALS-decreased DEGs (blue). In (E), (G) and (I), genes are ranked based upon their expression difference in ALS vs. CTL subjects (horizontal axis), and cumulative overlap of cell type signature genes is shown (vertical axis) (p-value, lower right, Wilcoxon rank sum test).

**Additional file 16.** ALS DEG exosome expression. (A) Comparison of blood mRNA and exosome protein changes. FC estimates from the current study (ALS/CTL) were compared to those estimated in protein exosomes by Tomlinson et al. [65]. Red symbols denote mRNAs and proteins with ALS-increased expression in both studies (mRNA: FC > 1.10, FDR < 0.10; protein: FC > 2.0, P < 0.05) and blue symbols denote mRNAs and proteins with ALS-decreased expression in both studies (mRNA: FC < 0.909, FDR < 0.10; protein: FC < 0.50, P < 0.05). The spearman rank correlation is shown (lower left) with robust regression fit (dotted green line). (B) ExoCarta exosome-associated mRNA GSEA analysis. (C) EVpedia exosome-associated mRNA GSEA analysis. In (B) and (C), genes are ranked based upon their expression change in ALS vs. CTL patients (horizontal axis) and cumulative overlap with exosome-associated mRNAs is shown (vertical axis) (p-values: Wilcoxon rank sum test). (D) Percentage of ALS DEGs (vertical axis) in groups of genes stratified according to average blood exosome expression (horizontal axis) (*n* = 32 subjects; yellow font or black asterisk: P < 0.05, Fisher’s exact test; GSE100206). (E) ALS-increased DEG GSEA analysis. (F) ALS-decreased DEG GSEA analysis. In (E) and (F), genes were ranked based upon their average expression in blood exosomes (*n* = 32 subjects; GSE100206) (horizontal axis), and cumulative ALS DEG abundance is shown (vertical axis). The analysis was repeated with DEGs selected at varying FC thresholds (see legend; top margin) and large above-diagonal (positive) or below-diagonal (negative) indicate bias towards high and low exosome expression, respectively (p-values: Wilcoxon rank sum test). (G) ALS-increased DEGs with highest mean exosome expression. (H) GO CC terms. Enrichment was evaluated with respect to the 100 ALS-increased DEGs with highest mean exosome expression (GSE100206). The 12 most significantly over-represented annotations are listed (conditional hypergeometric test). The number of genes associated with each annotation is listed (parentheses) with exemplar ALS-increased genes for each annotation.

**Additional file 17.** Single gene biomarkers for ALS diagnosis. (A, B) Cross-validation analysis of *NEFH* and *NEFL* prediction accuracy (10,000 simulations; logistic regression; training set: 296 ALS patients vs. 296 CTL/MIM subjects; testing set: 100 ALS patients vs. 100 CTL/MIM subjects). Histograms show the accuracy obtained across cross-validation trials. The proportion of trials in which accuracy was significantly greater than the non-information rate (NIR) of 50% is indicated (upper-right). (C) *NEFH* expression. Boxes outline the middle 50% of *Z*-score normalized expression values (whiskers: 10th to 90th percentiles). (D) ALS-increased DEGs most strongly decreased in MIM patients. Genes are ranked based upon the ALS/CTL FC estimate (parentheses, bottom margin; blue font: significantly decreased in MIM patients, FDR < 0.10, FC < 0.91). (E) ALS-decreased DEGs most strongly increased in MIM patients. Genes are ranked based upon the ALS/CTL FC estimate (parentheses, bottom margin). (F, G) AUC estimates. Boxes outline AUC 95% confidence intervals (middle bar: AUC point estimate; magenta font: 95% lower confidence limit > 0.50; upper margin: sensitivity/specificity). (H, I) Cross-validation analysis of *MCTP2* and *POLR3C* prediction accuracy (as above). (J) *MCTP2* expression (as above).

**Additional file 18.** Gene signature biomarkers for ALS diagnosis. (A) AUC estimates. Boxes outline AUC 95% confidence intervals (middle bar: AUC point estimate; magenta font: 95% lower confidence limit > 0.50; upper margin: sensitivity/specificity). (B) Neutrophil signature scores. Boxes outline the middle 50% of scores in each group (whiskers: 10th to 90th percentiles). (C) Cross-validation analysis of NP signature prediction accuracy (10,000 simulations; logistic regression; training set: 296 ALS patients vs. 296 CTL/MIM subjects; testing set: 100 ALS patients vs. 100 CTL/MIM subjects). Histograms show the accuracy obtained across cross-validation trials. The proportion of trials in which accuracy was significantly greater than the non-information rate (NIR) of 50% is indicated (i.e., McNemar’s test; upper-right). (D) AUC estimates (logistic regression bivariate models). The heatmap shows AUC estimates for each bivariate combination (diagonal: univariate model AUCs). The 3 highest AUC estimates for each row are numbered (1 = highest AUC). (E) NP vs. ALT signature scatterplot. Dotted lines denote the median NP and ALS values and the percentage of ALS, MIM and CTL patients in each quadrant is indicated (magenta line: least squares regression estimate). (F) Cross-validation analysis of NP + ALT signature prediction accuracy (as above).

**Additional file 19.** Published sensitivity and specificity estimates for ALS sample classification. The table lists the first author of each study and publication year, PubMed identifier (PMID), biofluid source, samples sizes (ALS and CTL groups), type of CTL group, biomarker or rule applied for classification, reported sensitivity (Sens) and specificity (Spec). For the “CTL type” column, values are healthy controls (HC), diseased control (DC), or the combination of healthy and diseased controls (HC + DC).

**Additional file 20.** Genes with survival-associated expression. (A, C) Genes with expression (A) negatively associated with survival (HR > 1.00) or (C) positively associated with survival (HR < 1.00). Hazard ratios were estimated using Cox PH models (covariates: age, sex, site of onset, and cohort; *n* = 396 patients). Top-ranked genes were selected from among 11,480 protein-coding genes with detectable expression in at least 20% of ALS patients (> 80/396). (B, D) Gene Ontology Biological Process terms. Enrichment was evaluated with respect to survival-associated genes (P < 0.01; B: HR > 0; C: HR < 0). The 12 most significantly over-represented annotations are listed (conditional hypergeometric test). The number of genes associated with each annotation is listed (parentheses) with exemplar survival-associated genes. (E–G) Cross-validation analysis of survival prediction accuracy (10,000 simulations; Cox PH model; training set: 296 ALS patients; testing set: 100 ALS patients). Concordance index distributions are shown for the base model (clinical covariates only) and full model (clinical covariates + expression of the indicated gene). Boxes (top) outline the middle 50% of outcomes (middle line: median; whiskers: 10th to 90th percentile). (H–J) Predicted survivorship with low (20th percentile) and high (80th percentile) expression of (H) *SPAG9*, (I) *KEL* and (J) *CCS*. Median survivorship for each group is shown (dashed lines) with the median ratio between groups (upper-right).

**Additional file 21.** Cell type and altitude signature scores association with survival. (A) Cell type signature scores and hazard ratios (HRs) (blue font: P < 0.05; *n* = 396 patients; covariates: age, sex, site of onset, and cohort). (B) Top 2-way score combinations. The top 12 score combinations are listed. The MC + NP combination is shown for comparison (red bar). (C) Top 3-way score combinations. In (B) and (C), p-values were calculated by comparing full models (cell type scores + covariates) to reduced models (covariates only) using likelihood ratio tests. (D–F) Cross validation evaluation of immune cell score prediction accuracy (10,000 simulations; Cox PH model; training set: 296 ALS patients; testing set: 100 ALS patients). Concordance index distributions are shown for the base model (covariates only) and full model (covariates + immune cell scores). Boxes (top) outline the middle 50% of outcomes (middle line: median; whiskers: 10th to 90th percentile). (G, H) Predicted survivorship with low (20th percentile) and high (80th percentile) signature scores. (I) Predicted survivorship with favorable and risk-associated expression signatures (RBC + PL + MP + covariates; favorable signature: patient with 80th percentile predicted survival time; risk signature: patient with 20th percentile predicted survival time). In (G)–(I), median survivorship for each group is shown (dashed lines) with the median ratio between groups (upper-right).

**Additional file 22.** M1 and M2 macrophage signature scores in myeloid and lymphoid patient subgroups. (A) Monocyte-lineage signature scores and hazard ratios (HRs). Scores were calculated by averaging Z-score normalized expression of the 150 genes most specifically expressed in each cell type. Composite myeloid (MYE) and lymphoid (LYM) scores are also shown (MYE: DC, MP, MC, NP, PL, RBC and ES; LYM: CD8, CD4, GDT, NK and B). Heatmap rows and columns are clustered (rows: 1–correlation; columns: Euclidean distance; right: hazard ratios; covariates: age, sex, site of onset, and cohort; *n* = 396 patients). (B) M1 macrophage signature scores. (C) M2 macrophage signature scores. In (B) and (C), the 396 patients are plotted with respect to myeloid and lymphoid signature scores and colors denote M1 or M2 scores for each patient. (D) M1 score group comparison. (E) M2 score group comparison. In (D) and (E), boxes outline the middle 50% of patients in each group (middle line: median) and whiskers span the 10th to 90th percentiles. The group median is shown (top margin) with shared letters (parentheses) indicating no significant difference among groups (P > 0.05; Fisher’s Least Significant Difference). The p-value (green font) was obtained by comparing scores from the myeloid and lymphoid groups (Wilcoxon rank sum test). (F) Genes with elevated expression in M1 macrophages (compared to non-polarized macrophages; GSE5099). (G) Genes with elevated expression in M2 macrophages (compared to non-polarized macrophages; GSE5099). In (F) and (G), boxes outline the middle 50% of expression values for lymphoid group patients, and error bars outline the middle 50% of values for myeloid group patients. Genes with a significant difference in expression between groups (FDR < 0.10) are shown in colored font (bottom margin).

**Additional file 23.** 61 gene signature for prediction of ALS patient survival. The table lists the 61 genes incorporated into the final Cox PH regression model developed to predict ALS patient survival. Columns 2 and 3 provide hazard ratio (HR) estimates from unigenic Cox PH models, which were fit with five predictor variables, including expression of the gene listed in column 1 with four covariates (age, sex, site of onset, cohort). The second column gives the HR estimate with 95% confidence limits (HR > 1: increased expression = worse survival; HR < 1: increased expression = improved survival). Columns 4 and 5 provide results from likelihood ratio tests (LRT) comparing goodness of fit between full models (61 predictor genes + 4 covariates) and reduced models (60 predictor genes + 4 covariates, with removal of the gene listed in column 1). The χ2 statistic obtained from each LRT is given (column 4) with p-value obtained from the χ2 null distribution with 1 degree of freedom (column 5). Genes for which expression contributes most to goodness of fit have larger χ2 statistics (and lower p-values).

## Data Availability

Raw and processed data analyzed in this study are available from Gene Expression Omnibus (GSE112676 and GSE112680).

## Abbreviations

ALS: amyotrophic lateral sclerosis
ALSFRS-R: revised ALS functional rating scale
ALT: altitude
AUC: area under curve
B: B-cell
C: concordance
CBD: corticobasal degeneration
CD4: CD4 T cell
CD8: CD8 T-cell
CIPD: chronic inflammatory demyelinating polyneuropathy
CRP: C-reactive protein
DC: diseased control
GDT: gamma-delta T-cell
CSF: cerebrospinal fluid
CTL: control subject
DC: dendritic cell
DEG: differentially expressed gene
DO: disease ontology
ES: eosinophil
FC: fold change
FDR: false discovery rate
FOSMN: facial onset sensory and motor neuronopathy
FVC: forced vital capacity
GEO: gene expression omnibus
GO: Gene Ontology
GSEA: gene set enrichment analysis
GWAS: genome-wide association study
HC: healthy control
HR: hazard ratio
HSP: hereditary spastic paraplegia
IQR: interquartile range
KEGG: Kyoto Encyclopedia of Genes and Genomes
KM: Kaplan-Meier
LYM: lymphoid
MA plot: M (log ratio) and A (mean average) scale plot
MC: monocyte
MIM: patient with ALS-mimic disease
MMN: multifocal motor neuropathy
MP: macrophage
MYE: myeloid
NK: NK-cell
NP: neutrophil
OOB: out-of-bag
OOBA: out-of-bag accuracy
PBMC: peripheral blood mononuclear cells
PC: principal component
PH: proportional hazards
PL: platelet
PMID: pubMed identifier
RBC: red blood cell
RMA: robust multichip average
RNA-seq: RNA sequencing
RSV: respiratory syncytial virus
RZE: riluzole
SENS: sensitivity
SMA: spinal muscular atrophy
SPEC: specificity
SVA: surrogate variable analysis
SVM: support vector machine
TPM: transcript per million
