# Supplementary material for: ALS blood expression profiling identifies new biomarkers, patient subgroups, and evidence for neutrophilia and hypoxia
Source: J Transl Med. 2019 May 22;17:170. doi: 10.1186/s12967-019-1909-0 (PMC6530130; doi:10.1186/s12967-019-1909-0)
Supplement: Supplementary file 1 — Additional file 1. Whole-genome expression analyses of ALS and CTL patient blood samples. Table columns list the first author with publication year, gene expression profiling platform, PubMed identifier, GEO series accession (if available), cell types analyzed, and the number of samples used for expression profiling. Only studies of blood-derived cell types are included. Full references are listed below the table with additional relevant information (see footnotes). [file 12967_2019_1909_MOESM1_ESM.pdf]

Additional File 1. Whole-genome expression analyses of ALS and CTL patient blood samples. Table columns list the first author with publication year, gene expression profiling platform, PubMed identifier, GEO series accession (if available), cell types analyzed, and the number of samples used for expression profiling. Only studies of blood-derived cell types are included. Full references are listed below the table with additional relevant information (see footnotes).

**Study**	**Platform**	**PubMed**	**GEO Series**	**Tissue**	***n* (ALS)**	***n* (CTL)**
Saris 2009[a]	Illumina HumanRef-8 BeadChip	19712483	None	Whole blood	30[†]	30[†]
Zhang 2011[b]	Affymetrix HG-U133 Plus 2.0 Array	20884065	None	PBMCs	20	22
Mougeot 2011[c]	Agilent Whole Genome 4 × 44k Array	22027401	GSE28253	Lymphocytes	11	11
Lam 2015[d]	Illumina HiSeq 2000	26807342	None	PBMCs	9	1
Zhao 2017[e]	Illumina HiSeq 1500	28437540	None	Monocytes	43	22
Gagliardi 2018[f]	Illumina NextSeq 500	29402919	GSE115259	PBMCs	7	4
van Rheenen 2018[g]	Illumina HumanHT-12 V3.0 Beadchip	29939990	GSE112676	Whole blood	233	508
van Rheenen 2018[g]	Illumina HumanHT-12 V4.0 Beadchip	29939990	GSE112680	Whole blood	164	137

[a]Saris CG, Horvath S, van Vught PW, van Es MA, Blauw HM, Fuller TF, Langfelder P, DeYoung J, Wokke JH, Veldink JH, van den Berg LH, Ophoff RA. 2009. Weighted gene co-expression network analysis of the peripheral blood from Amyotrophic Lateral Sclerosis patients. BMC Genomics 10:405.

[b]Zhang R, Hadlock KG, Do H, Yu S, Honrada R, Champion S, Forshew D, Madison C, Katz J, Miller RG, McGrath MS. 2011. Gene expression profiling in peripheral blood mononuclear cells from patients with sporadic amyotrophic lateral sclerosis (sALS). J Neuroimmunol 230:114-123.

[c]Mougeot JL, Li Z, Price AE, Wright FA, Brooks BR. 2011. Microarray analysis of peripheral blood lymphocytes from ALS patients and the SAFE detection of the KEGG ALS pathway. BMC Med Genomics 4:74.

[d]Lam L, Halder RC, Montoya DJ, Rubbi L, Rinaldi A, Sagong B, Weitzman S, Rubattino R, Singh RR, Pellegrini M, Fiala M. 2015. Anti-inflammatory therapies of amyotrophic lateral sclerosis guided by immune pathways. Am J Neurodegener Dis 4:28-39.

[e]Zhao W, Beers DR, Hooten KG, Sieglaff DH, Zhang A, Kalyana-Sundaram S, Traini CM, Halsey WS, Hughes AM, Sathe GM, Livi GP, Fan GH, Appel SH. 2017. Characterization of Gene Expression Phenotype in Amyotrophic Lateral Sclerosis Monocytes. JAMA Neurol 74:677-685.

[f]Gagliardi S, Zucca S, Pandini C, Diamanti L, Bordoni M, Sproviero D, Arigoni M, Olivero M, Pansarasa O, Ceroni M, Calogero R, Cereda C. 2018. Long non-coding and coding RNAs characterization in Peripheral Blood Mononuclear Cells and Spinal Cord from Amyotrophic Lateral Sclerosis patients. Sci Rep 8:2378.

[g]van Rheenen W, Diekstra FP, Harschnitz O, Westeneng HJ, van Eijk KR, Saris CGJ, Groen EJN, van Es MA, Blauw HM, van Vught PWJ, Veldink JH, van den Berg LH. 2011. Whole blood transcriptome analysis in amyotrophic lateral sclerosis: A biomarker study. PLoS ONE 13:e0198874.

[†]The 30 samples listed correspond to the "discovery cohort" used to identify differentially expressed genes. This study included an additional 93 ALS and 93 CLT patients in two additional "replication" datasets (used to confirm findings discovery cohort findings).
