# Supplementary figures and images for: ALS blood expression profiling identifies new biomarkers, patient subgroups, and evidence for neutrophilia and hypoxia
Source: J Transl Med. 2019 May 22;17:170. doi: 10.1186/s12967-019-1909-0 (PMC6530130; doi:10.1186/s12967-019-1909-0)

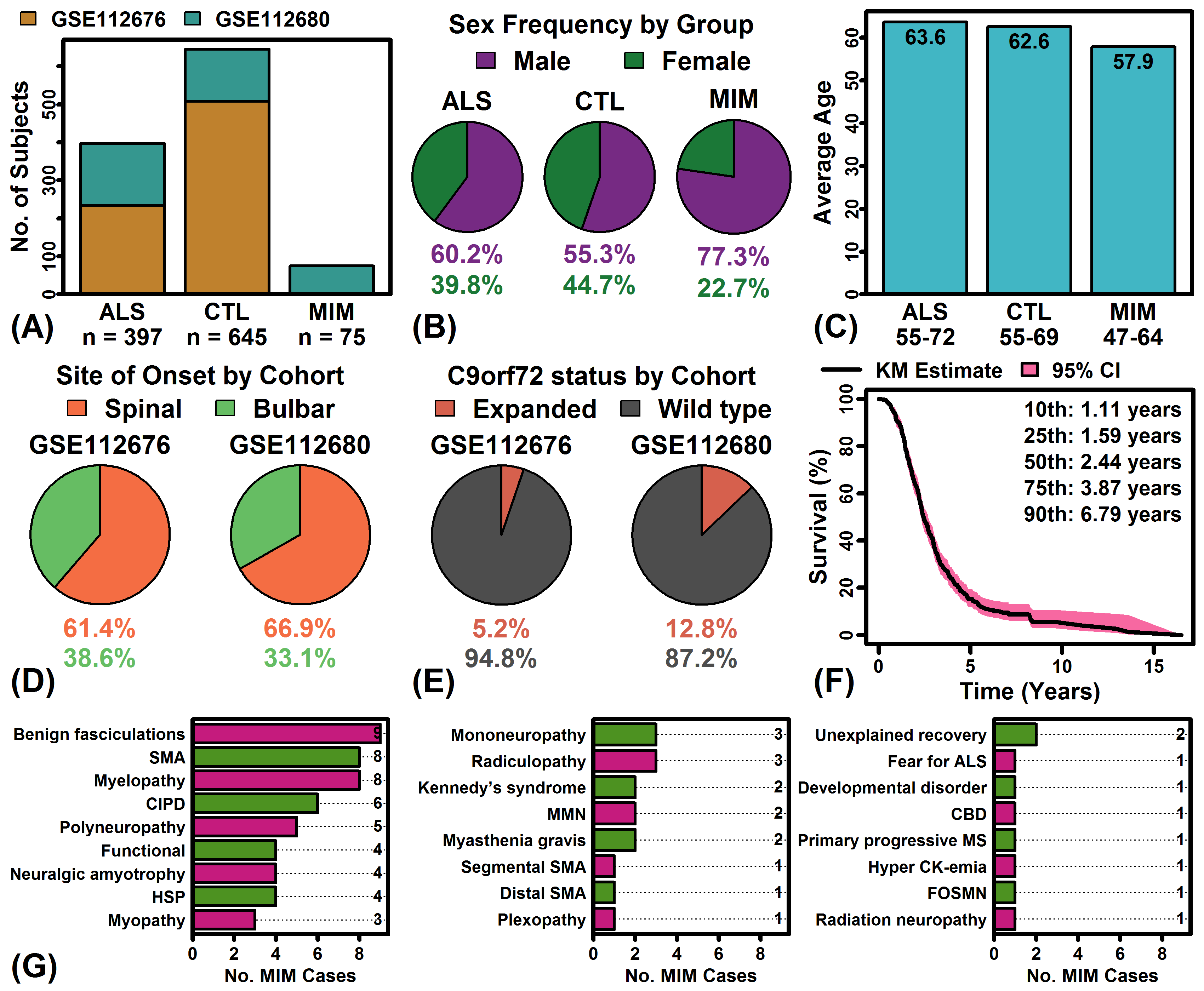

Supplement: Supplementary file 2 — Additional file 2. Description of patient cohorts. (A) Number of subjects per group. (B) Male and female frequencies. (C) Average age per group. (D) Site of onset. (E) C9orf72 status. (F) ALS patient survival (n = 397). The Kaplan–Meier (KM) survival estimate is shown with 95% confidence intervals (upper right: survivorship quantiles). (G) MIM cohort diagnoses (n = 75). The frequency of cases is shown for each condition (CBD: corticobasal degeneration; CIPD: chronic inflammatory demyelinating polyneuropathy; FOSMN: facial onset sensory and motor neuronopathy; HSP: hereditary spastic paraplegia; MMN: multifocal motor neuropathy; SMA: spinal muscular atrophy). [file 12967_2019_1909_MOESM2_ESM.tif]

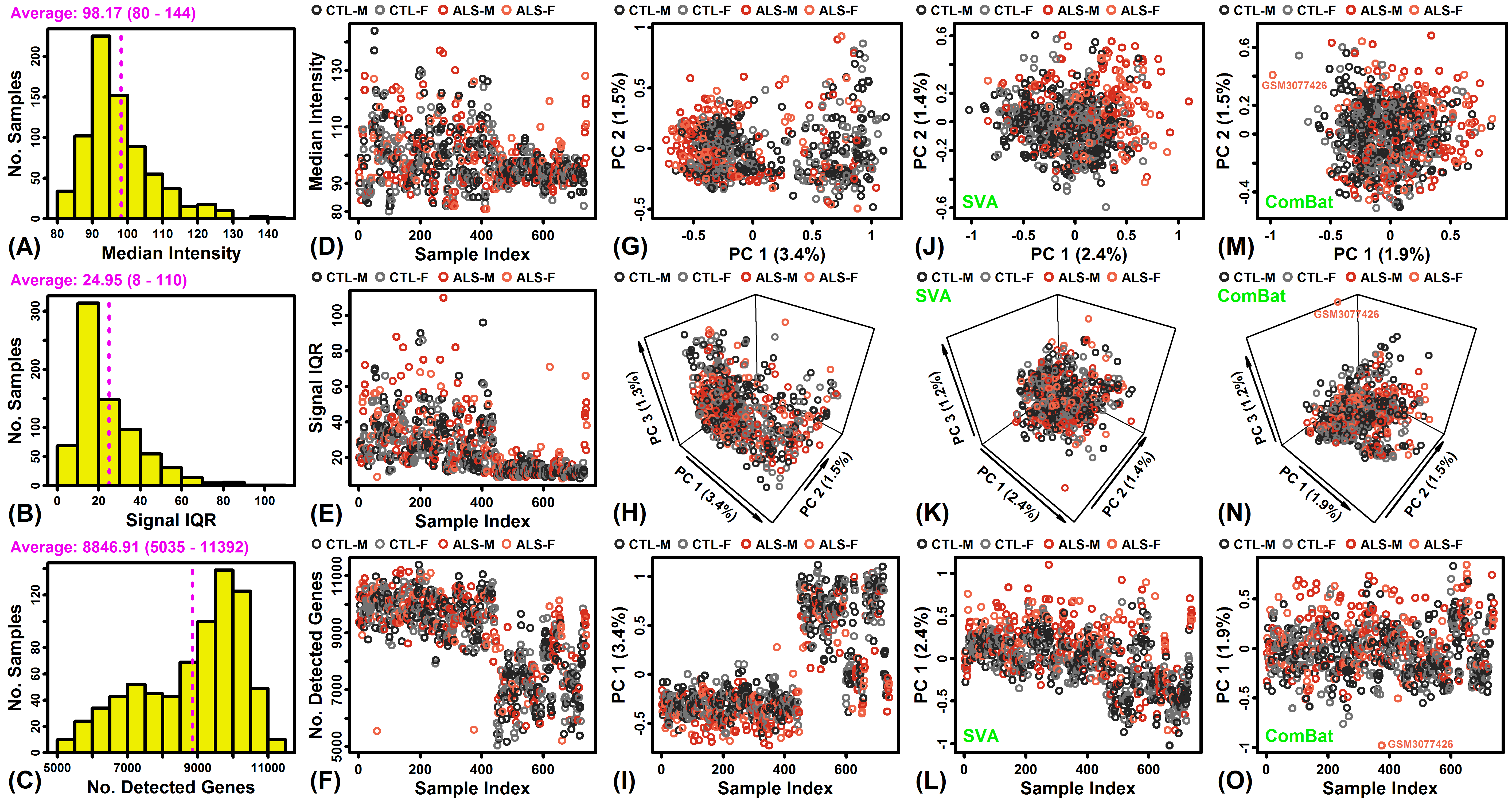

Supplement: Supplementary file 3 — Additional file 3. Microarray normalization and batch adjustment (GSE112676). (A) Median signal intensity histogram. (B) Interquartile range (IQR) histogram. (C) Number of protein-coding genes with detectable expression histogram. In (A–C), histograms show the distribution among 741 samples, and the median and range is given (top margin). (D) Median intensity sample index plot. (E) Interquartile range sample index plot. (F) Number of protein-coding genes with detectable expression sample index plot. In (D–F), the horizontal axis corresponds to the ordering of samples as listed in the GEO entry (GSM3076582–GSM3078510). (G) PC plot (2 dimensions). (H) PC plot (3 dimensions). (I) PC 1 sample index plot. (J) PC plot (2 dimensions) after SVA batch adjustment. (K) PC plot (3 dimensions) after SVA batch adjustment. (L) PC 1 sample index plot after SVA batch adjustment. (M) PC plot (2 dimensions) after ComBat batch adjustment. (N) PC plot (3 dimensions) after ComBat batch adjustment. (O) PC 1 sample index plot after ComBat batch adjustment. In (M)–(O), the sample designated as an outlier and removed from analyses is indicated (GSM3077426). [file 12967_2019_1909_MOESM3_ESM.tif]

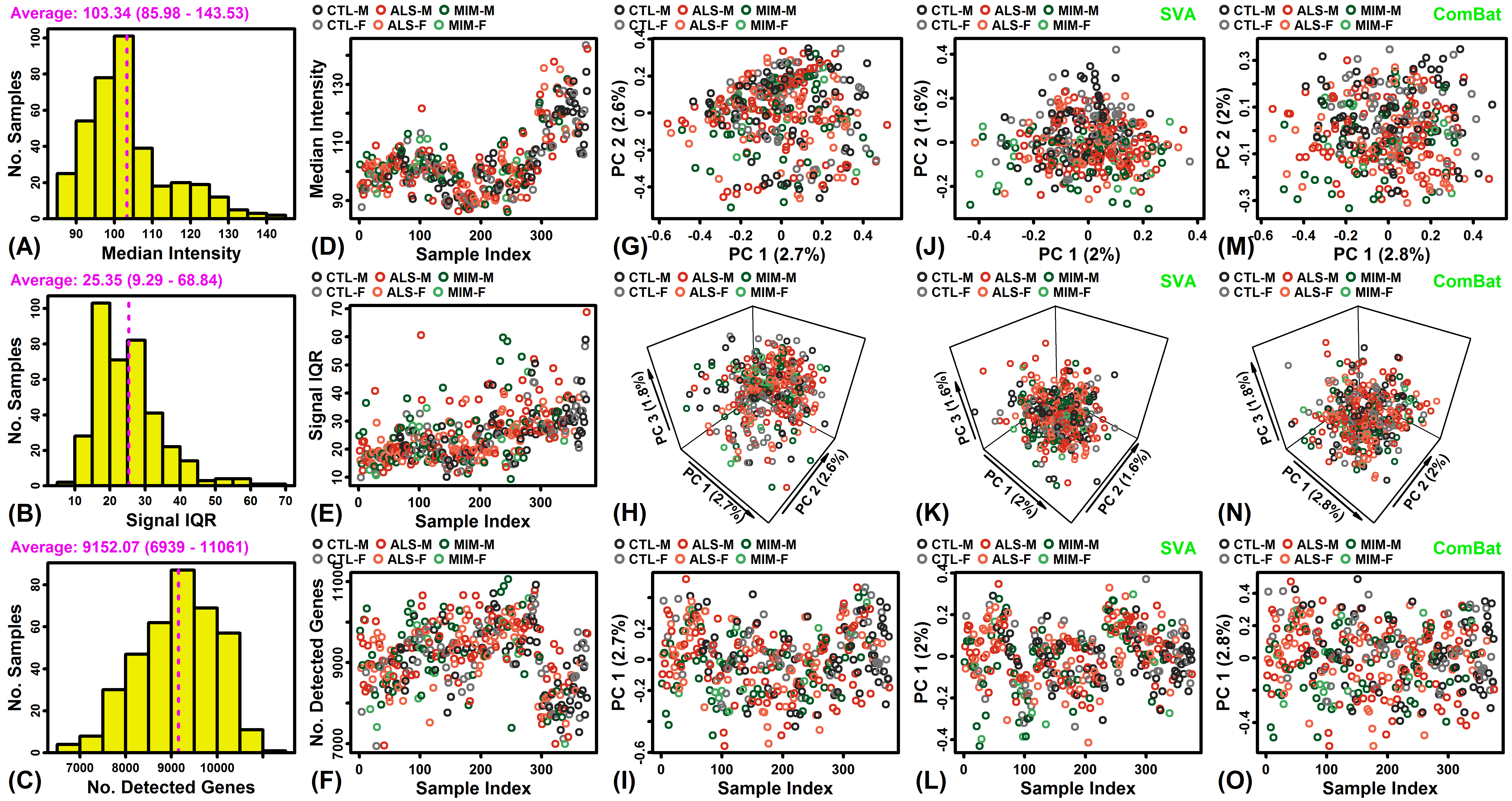

Supplement: Supplementary file 4 — Additional file 4. Microarray normalization and batch adjustment (GSE112680). (A) Median signal intensity histogram. (B) Interquartile range (IQR) histogram. (C) Number of protein-coding genes with detectable expression histogram. In (A–C), histograms show the distribution among 376 samples, and the median and range is given (top margin). (D) Median intensity sample index plot. (E) Interquartile range sample index plot. (F) Number of protein-coding genes with detectable expression sample index plot. In (D–F), the horizontal axis corresponds to the ordering of samples as listed in the GEO entry (GSM3076582–GSM3078510). (G) PC plot (2 dimensions). (H) PC plot (3 dimensions). (I) PC 1 sample index plot. (J) PC plot (2 dimensions) after SVA batch adjustment. (K) PC plot (3 dimensions) after SVA batch adjustment. (L) PC 1 sample index plot after SVA batch adjustment. (M) PC plot (2 dimensions) after ComBat batch adjustment. (N) PC plot (3 dimensions) after ComBat batch adjustment. (O) PC 1 sample index plot after ComBat batch adjustment. [file 12967_2019_1909_MOESM4_ESM.tif]

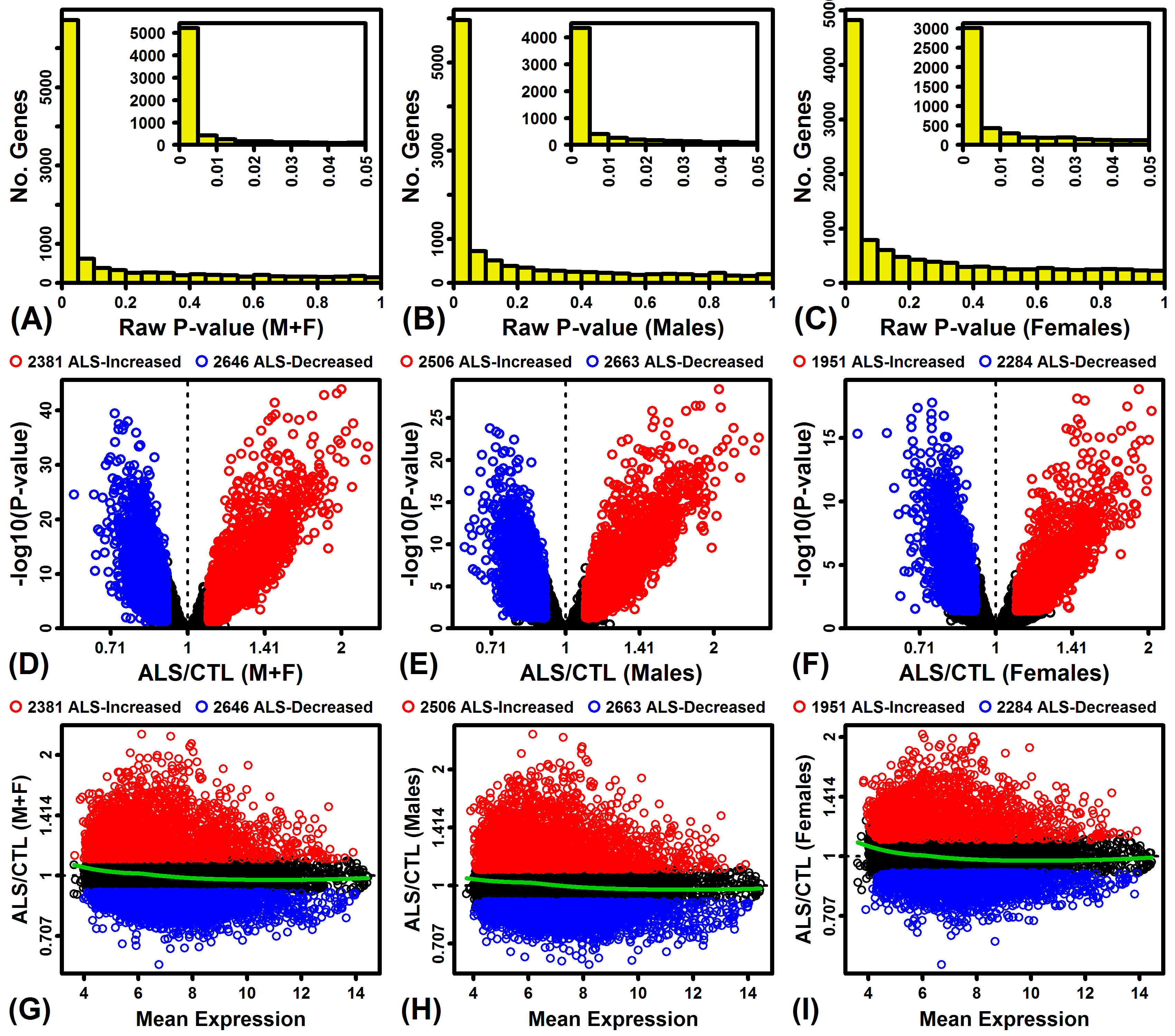

Supplement: Supplementary file 5 — Additional file 5. Differential expression plots (GSE112676). (A) P-value distribution (males + females). (B) P-value distribution (male only analysis). (C) P-value distribution (female only analysis). In (A)–(C), the distribution of raw p-values among protein-coding genes is shown. The inset (upper right) shows the distribution of p-values less than 0.05. (D) Volcano plot (males + females). (E) Volcano plot (male only analysis). (F) Volcano plot (female only analysis). (G) MA plot (males + females). (H) MA plot (males only analysis). (I) MA plot (female only analysis). In (D)–(I), the number of increased (red) and decreased (blue) DEGs is shown in the upper margin (FDR < 0.10 with FC > 1.10 or FC < 0.91). [file 12967_2019_1909_MOESM5_ESM.tif]

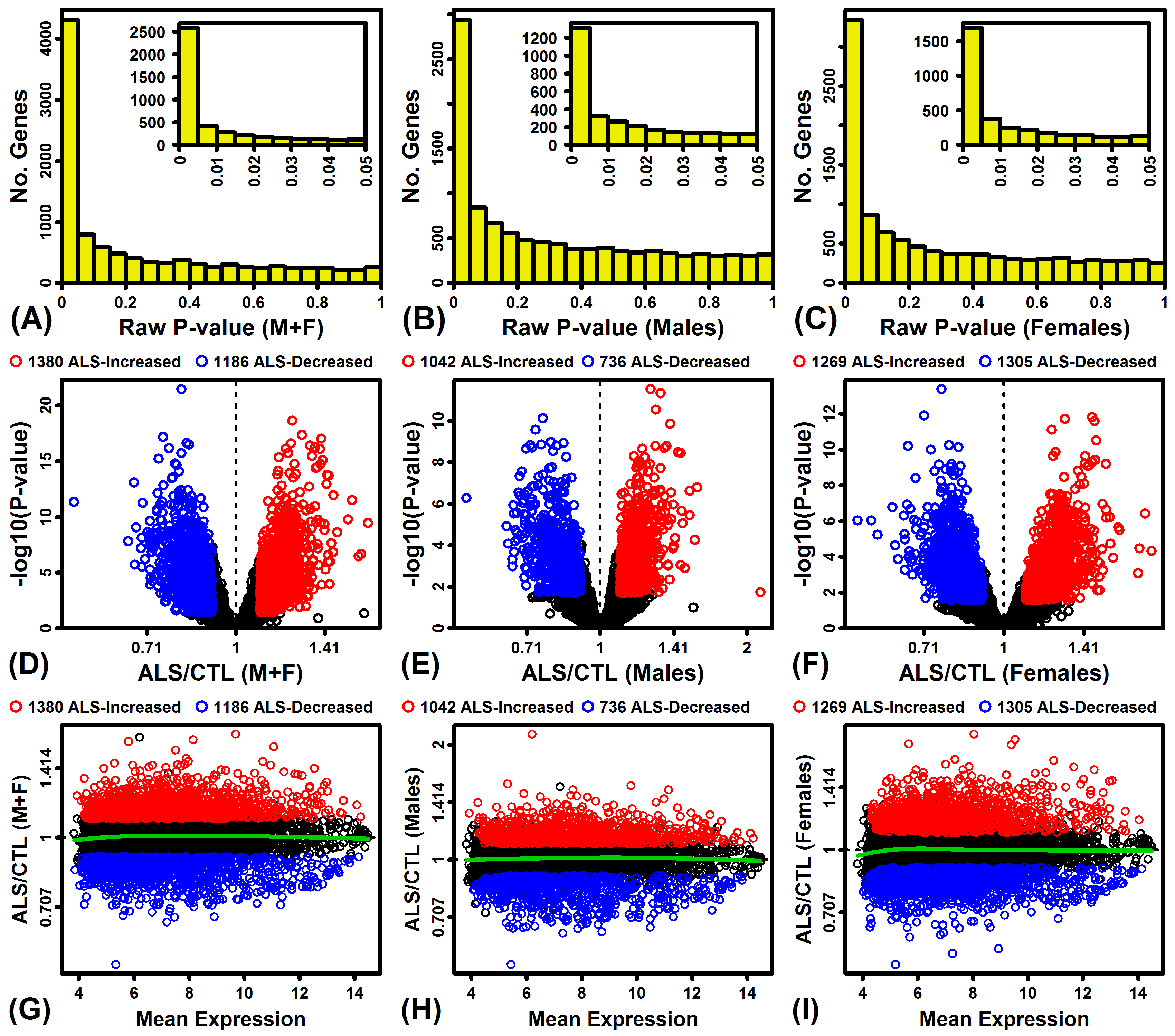

Supplement: Supplementary file 6 — Additional file 6. Differential expression plots (GSE112680). (A) P-value distribution (males + females). (B) P-value distribution (male only analysis). (C) P-value distribution (female only analysis). In (A)–(C), the distribution of raw p-values among protein-coding genes is shown. The inset (upper right) shows the distribution of p-values less than 0.05. (D) Volcano plot (males + females). (E) Volcano plot (male only analysis). (F) Volcano plot (female only analysis). (G) MA plot (males + females). (H) MA plot (males only analysis). (I) MA plot (female only analysis). In (D)–(I), the number of increased (red) and decreased (blue) DEGs is shown in the upper margin (FDR < 0.10 with FC > 1.10 or FC < 0.91). [file 12967_2019_1909_MOESM6_ESM.tif]

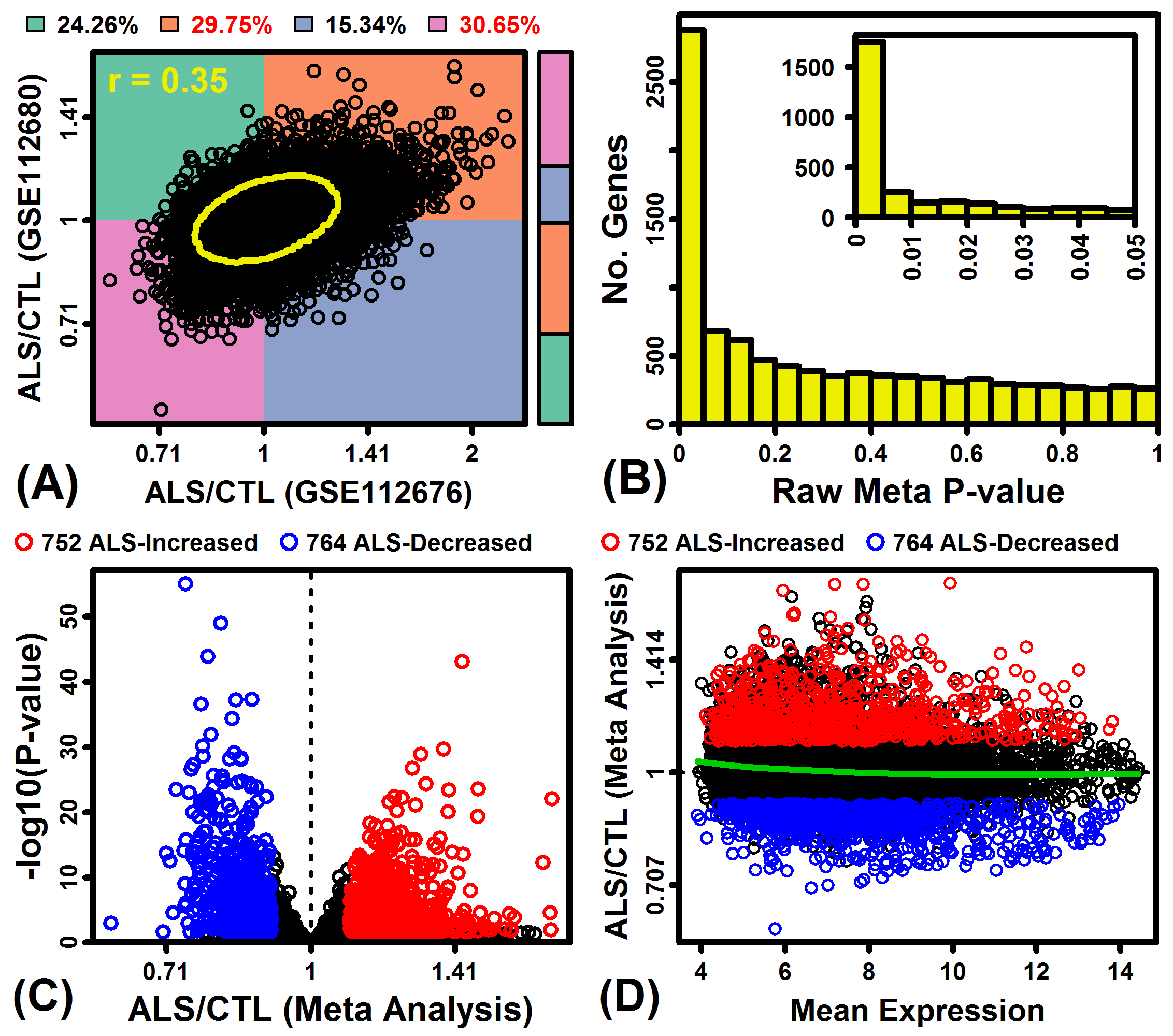

Supplement: Supplementary file 7 — Additional file 7. Meta-analysis differential expression plots. (A) FC scatterplot (GSE112676 versus GSE112680; 9822 protein-coding genes). The yellow ellipse outlines the middle 50% of genes closest to the bivariate median (Mahalanobis distance). The spearman rank correlation coefficient is shown (upper-left). The percentage of genes in each quadrant is indicated (top margin; red font: P < 0.05, Fisher’s exact test). The color-coded vertical bar (right margin) reflects the proportion of genes in each quadrant. (B) P-value distribution (inset: p-values less than 0.05). (C) Volcano plot. (D) MA plot. In (C) and (D), the number of increased (red) and decreased (blue) DEGs is shown in the upper margin (FDR < 0.10 with FC > 1.10 or FC < 0.91). [file 12967_2019_1909_MOESM7_ESM.tif]

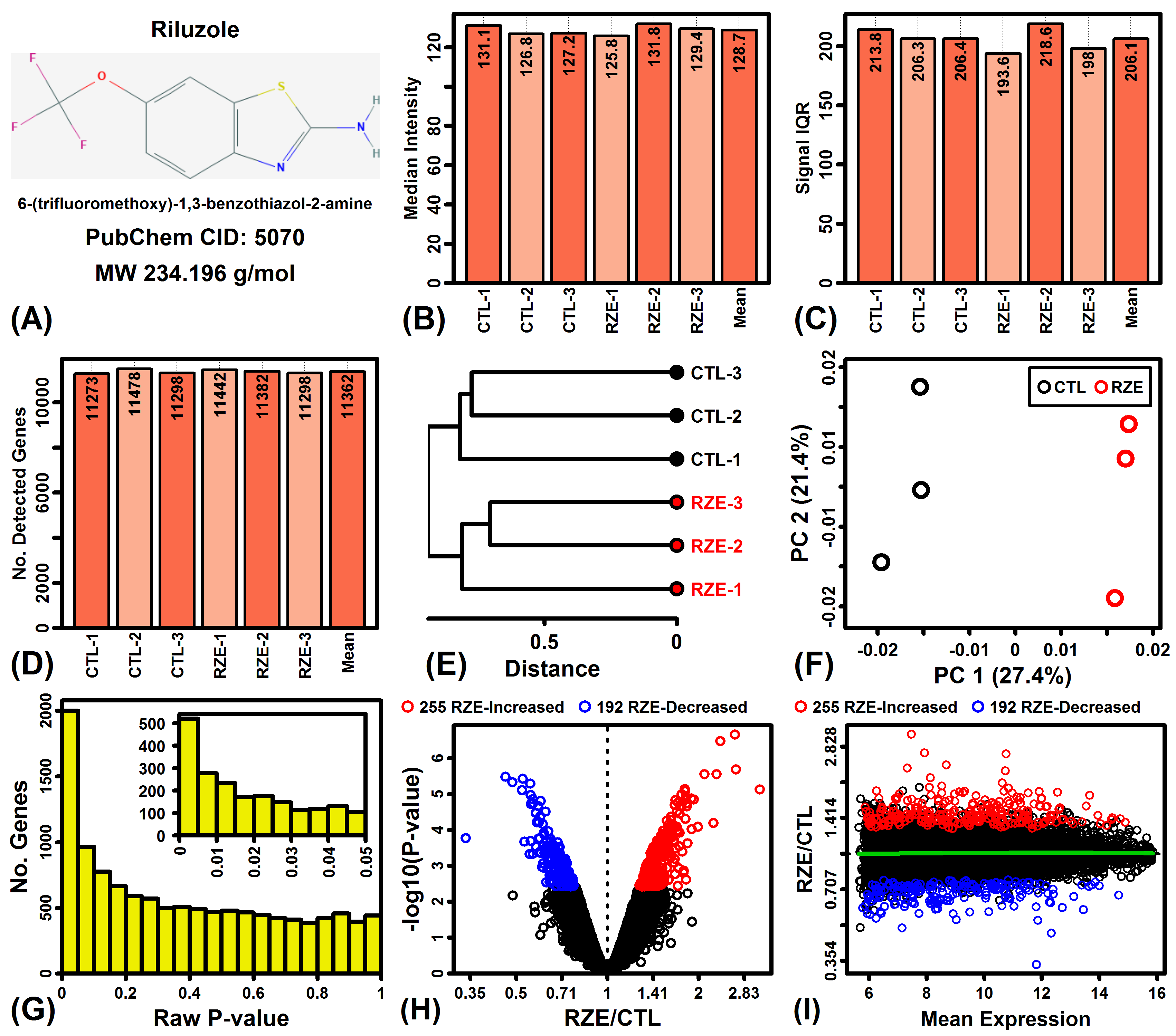

Supplement: Supplementary file 8 — Additional file 8. Gene expression responses to riluzole (GSE96653). (A) Riluzole chemical structure. (B) Median intensity prior to quantile normalization. (C) Signal IQR prior to quantile normalization. (D) Number of protein-coding genes with detectable expression in each sample (P < 0.05). (E) Cluster analysis. The 6 samples were clustered hierarchically based upon the Euclidean distance with average linkage. (F) PC plot. The 6 samples are plotted with respect to the first 2 PC axes. (G) Differential expression analysis raw p-value distribution (RZE vs. CTL; 11,868 protein-coding genes). (H) Volcano plot. (I) MA plot. In (H) and (I), the number of increased (red) and decreased (blue) DEGs is shown in the upper margin (FDR < 0.10 with FC > 1.10 or FC < 0.91). [file 12967_2019_1909_MOESM8_ESM.tif]

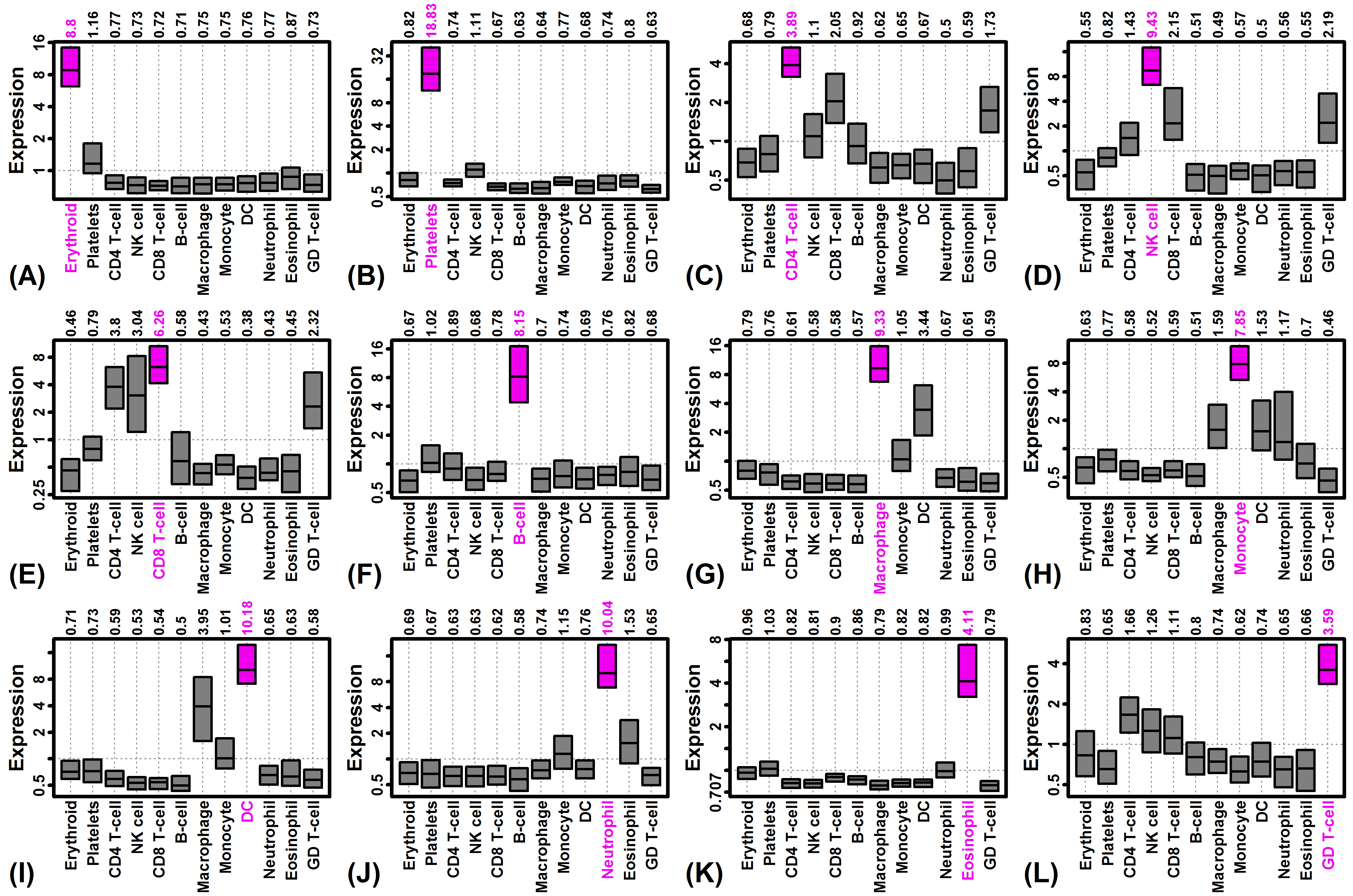

Supplement: Supplementary file 9 — Additional file 9. Signature gene expression in 12 cell types. (A) Erythroid lineage. (B) Platelets. (C) CD4 + T cells. (D) NK cells. (E) CD8 + T cells. (F) B cells. (G) Macrophages. (H) Monocytes. (I) Dendritic cells. (J) Neutrophils. (K) Eosinophils. (L) Gamma-delta T cells. In (A)–(L), expression is shown for the 100 signature genes identified for each cell type. Boxes outline average expression among samples for the middle 50% of signature genes (midline: median expression). Median expression for each cell type is listed in the top margin. Relative gene expression is normalized to an average value of 1 across the 12 cell types. [file 12967_2019_1909_MOESM9_ESM.tif]

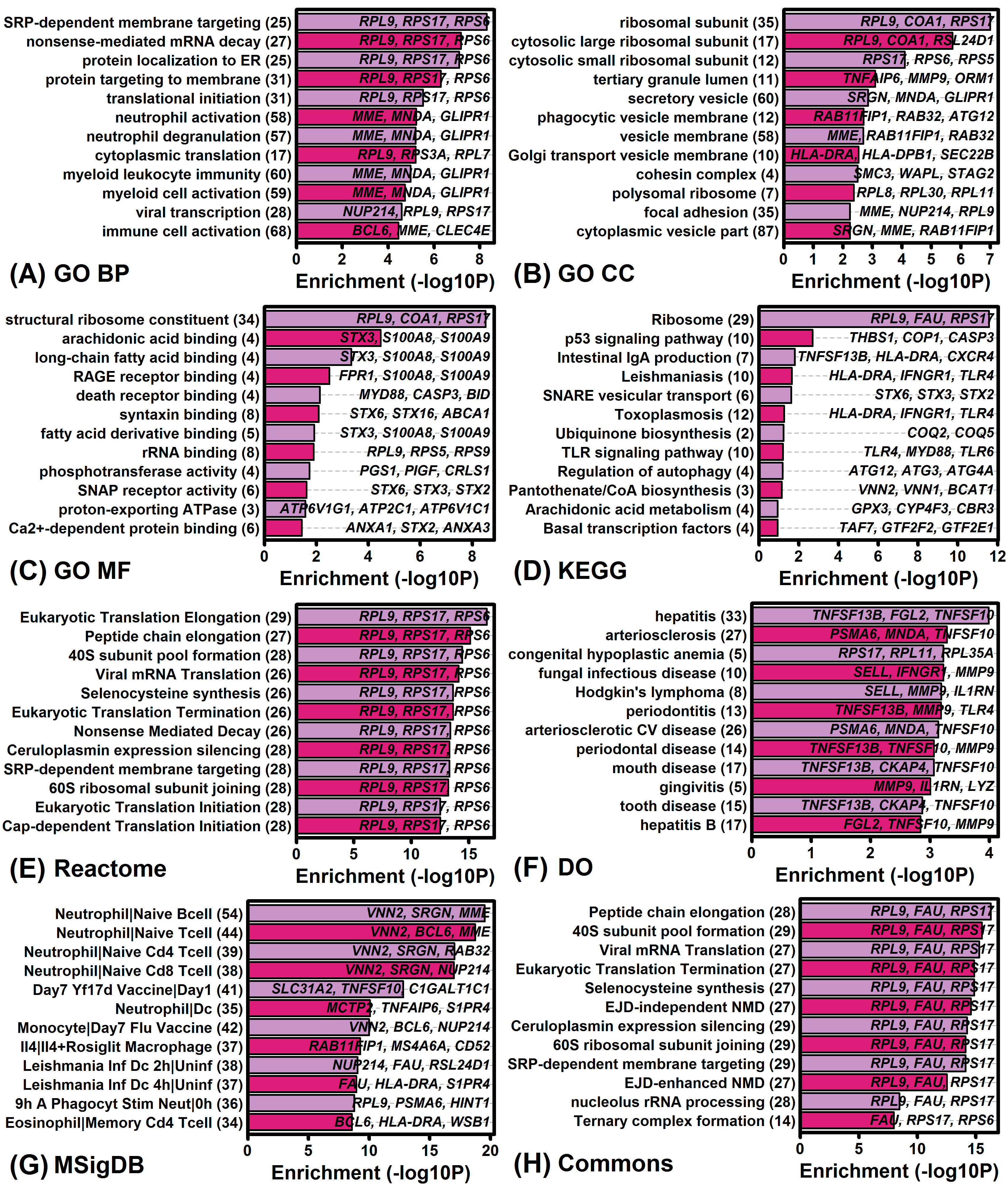

Supplement: Supplementary file 11 — Additional file 11. Gene annotations enriched among ALS-increased DEGs. (A) Gene Ontology biological processes. (B) Gene Ontology cell components. (C) Gene Ontology molecular functions. (D) Kyoto Encyclopedia of Genes and Genomes (KEGG) pathways. (E) Reactome pathway database. (F) Disease Ontology. (G) Molecular signatures database (MSigDB). (H) Pathway commons. In (A)–(H), enrichment was evaluated with respect to 580 ALS-increased DEGs (FC > 1.10 with FDR < 0.10). The 12 most significantly over-represented annotations are listed for each analysis (Fisher’s exact test or conditional hypergeometric test). The number of genes associated with each annotation is listed in parentheses, and exemplar ALS-increased genes associated with each annotation are shown. In part (G), MSigDB annotations correspond to gene sets generated from the comparison of two sample groups (X|Y), with genes in each set having higher expression in group X as compared to group Y. [file 12967_2019_1909_MOESM11_ESM.tif]

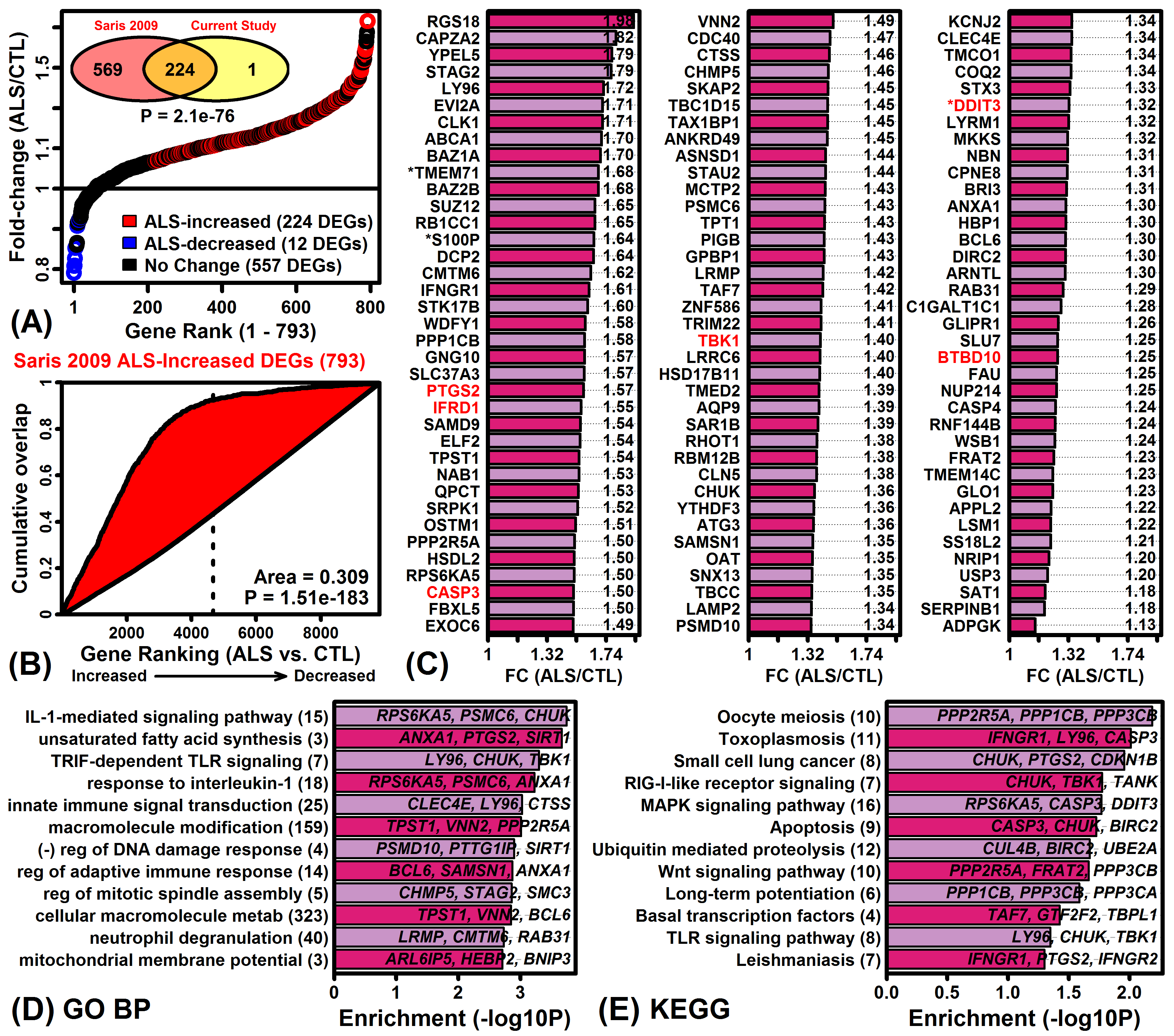

Supplement: Supplementary file 12 — Additional file 12. ALS-increased DEG overlap with genes identified by Saris et al. [32]. (A) FC distribution for 793 genes previously identified as elevated in ALS patient whole blood (red symbols: ALS-increased DEGs, FDR < 0.10, FC > 1.10; blue symbols: ALS-decreased DEGs, FDR < 0.10, FC < 0.91). The Venn diagram (top-left) shows the overlap between ALS-increased genes from both studies (p-value: Fisher’s exact test). (B) GSEA analysis. Genes are ranked based upon their expression difference in ALS vs. CTL subjects from the current study (horizontal axis), and cumulative overlap with ALS-increased genes from Saris et al. [32] is shown (vertical axis) (p-value, lower right, Wilcoxon rank sum test). (C) Top ALS-increased DEGs ranked by FC (red font: ALS-associated genes; *riluzole-increased DEG, FDR < 0.10). Meta-FC estimates were obtained using a random effects meta-analysis model to integrate results from all 3 blood studies (i.e., GSE112676, GSE112680 and Saris et al. [32]). (D) Gene Ontology biological processes. (E) Kyoto Encyclopedia of Genes and Genomes (KEGG) pathways. In (D) and (E), enrichment was evaluated with respect to 572 ALS-increased DEGs (FC > 0.90 with FDR < 0.10). The 12 most significantly over-represented annotations are listed for each analysis (Fisher’s exact test or conditional hypergeometric test). The number of genes associated with each annotation is listed in parentheses, and exemplar ALS-increased genes associated with each annotation are shown. [file 12967_2019_1909_MOESM12_ESM.tif]

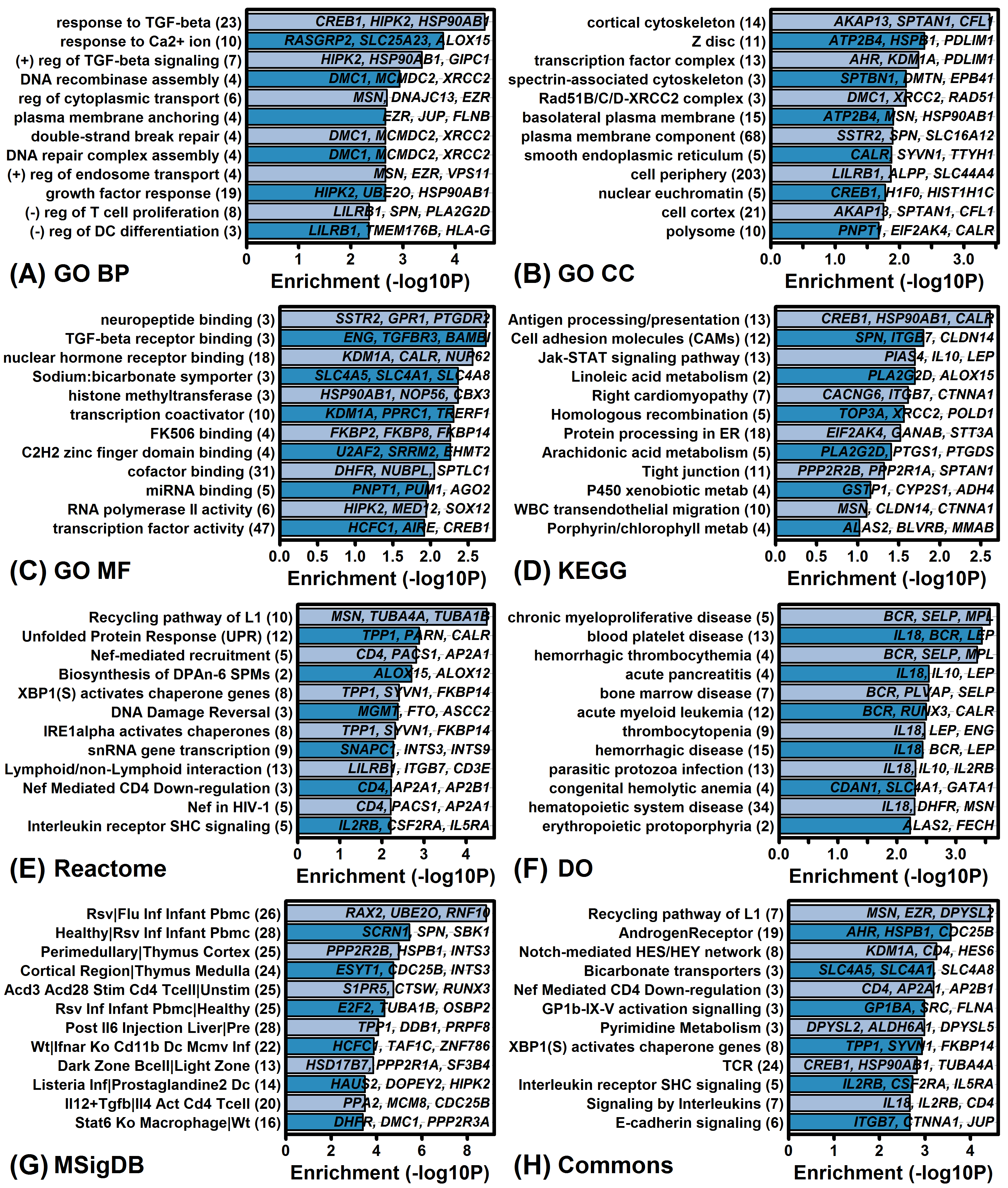

Supplement: Supplementary file 13 — Additional file 13. Gene annotations enriched among ALS-decreased DEGs. (A) Gene Ontology biological processes. (B) Gene Ontology cell components. (C) Gene Ontology molecular functions. (D) Kyoto Encyclopedia of Genes and Genomes (KEGG) pathways. (E) Reactome pathway database. (F) Disease Ontology. (G) Molecular signatures database (MSigDB). (H) Pathway commons. In (A)–(H), enrichment was evaluated with respect to 666 ALS-decreased DEGs (FC < 0.91 with FDR < 0.10). The 12 most significantly over-represented annotations are listed for each analysis (Fisher’s exact test or conditional hypergeometric test). The number of genes associated with each annotation is listed in parentheses, and exemplar ALS-decreased genes associated with each annotation are shown. In part (G), MSigDB annotations correspond to gene sets generated from the comparison of two sample groups (X|Y), with genes in each set having higher expression in group X as compared to group Y. [file 12967_2019_1909_MOESM13_ESM.tif]

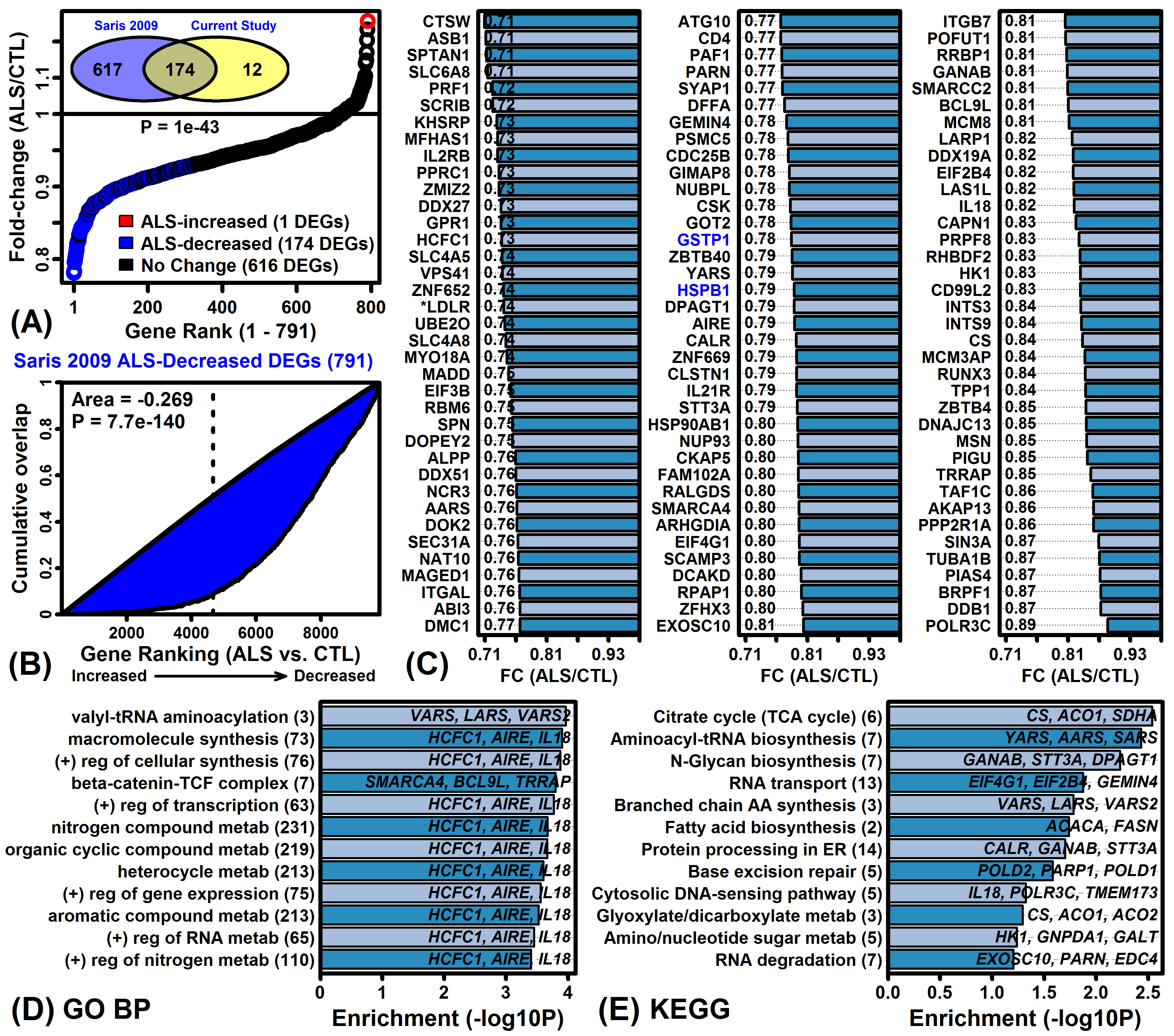

Supplement: Supplementary file 14 — Additional file 14. ALS-decreased DEG overlap with genes identified by Saris et al. [32]. (A) FC distribution for 791 genes previously identified as decreased in ALS patient whole blood (red symbols: ALS-increased DEGs, FDR < 0.10, FC > 1.10; blue symbols: ALS-decreased DEGs, FDR < 0.10, FC < 0.91). The Venn diagram (top-left) shows the overlap between ALS-decreased genes from both studies (p-value: Fisher’s exact test). (B) GSEA analysis. Genes are ranked based upon their expression difference in ALS vs. CTL subjects from the current study (horizontal axis), and cumulative overlap with ALS-decreased genes from Saris et al. [32] is shown (vertical axis) (p-value, lower right, Wilcoxon rank sum test). (C) Top ALS-decreased DEGs ranked by FC (blue font: ALS-associated genes; *riluzole-decreased DEG, FDR < 0.10). Meta-FC estimates were obtained using a random effects meta-analysis model to integrate results from all 3 blood studies (i.e., GSE112676, GSE112680 and Saris et al. [32]. (D) Gene Ontology biological processes. (E) Kyoto Encyclopedia of Genes and Genomes (KEGG) pathways. In (D) and (E), enrichment was evaluated with respect to 441 ALS-decreased DEGs (FC < 0.91 with FDR < 0.10). The 12 most significantly over-represented annotations are listed for each analysis (Fisher’s exact test or conditional hypergeometric test). The number of genes associated with each annotation is listed in parentheses, and exemplar ALS-decreased genes associated with each annotation are shown. [file 12967_2019_1909_MOESM14_ESM.tif]

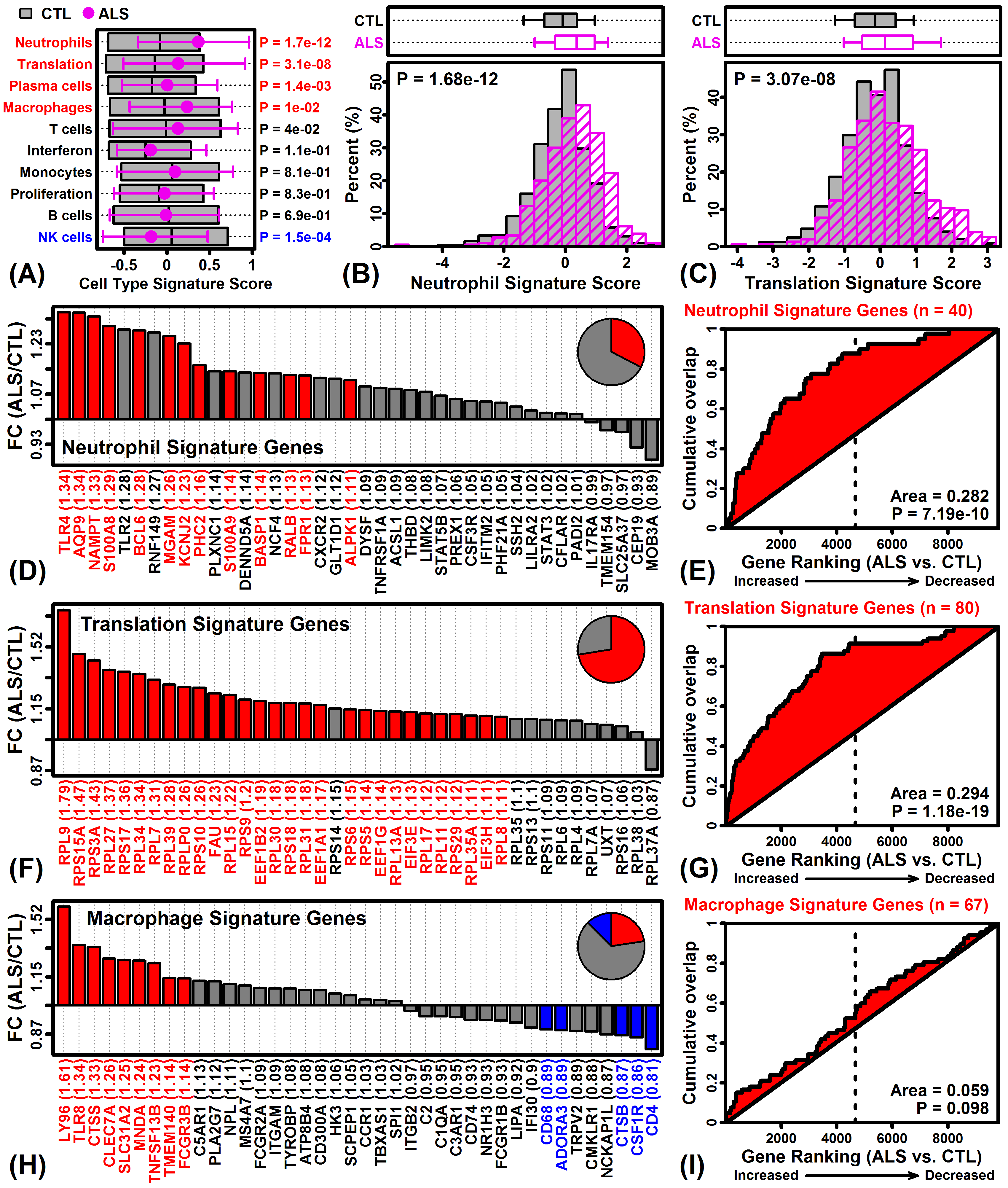

Supplement: Supplementary file 15 — Additional file 15. Cell type enrichment (ImSig algorithm). (A) Cell type ranking. Grey boxes outline the middle 50% of ImSig cell type scores for CTL subjects (n = 645; middle line: median). Magenta bars span the middle 50% of ImSig scores for ALS patients (n = 396; circle: median). P-values obtained from the test of an ALS vs. CTL score difference are listed (right margin; two-tailed t-test; red: FDR < 0.05 with ALS > CTL; blue: FDR < 0.05 with CTL > ALS). Cell type scores (horizontal axis) were normalized using a Z-score transformation and combined across the two cohorts (GSE112676 and GSE112680). (B) Neutrophil score histograms. (C) Translation score histograms. In (B) and (C), cell type scores from each cohort (GSE112676 and GSE112680) were normalized using the Z-score transformation and expression values from the two cohorts were combined. Boxplots (top margin) outline the middle 50% of expression values in each group (whiskers: 10th to 90th percentile; p-values: two-tailed t-test). (D) Top 40 neutrophil signature genes with lowest p-value (ALS vs. CTL). (E) Neutrophil signature gene GSEA analysis. (F) Top 40 translation signature genes with lowest p-value (ALS vs. CTL). (G) Translation signature GSEA analysis. (H) Top 40 macrophage signature genes with lowest p-value (ALS vs. CTL). (I) Macrophage signature GSEA analysis. In (D), (F) and (H), red bars/font indicates ALS-increased DEGs (FDR < 0.10 with FC > 1.10) and blue bars/font indicates ALS-decreased DEGs (FDR < 0.10 with FC < 0.91). The pie chart (upper right) indicates the proportion of genes not significantly altered in the ALS vs. CTL comparison (black), ALS-increased DEGs (red) and ALS-decreased DEGs (blue). In (E), (G) and (I), genes are ranked based upon their expression difference in ALS vs. CTL subjects (horizontal axis), and cumulative overlap of cell type signature genes is shown (vertical axis) (p-value, lower right, Wilcoxon rank sum test). [file 12967_2019_1909_MOESM15_ESM.tif]

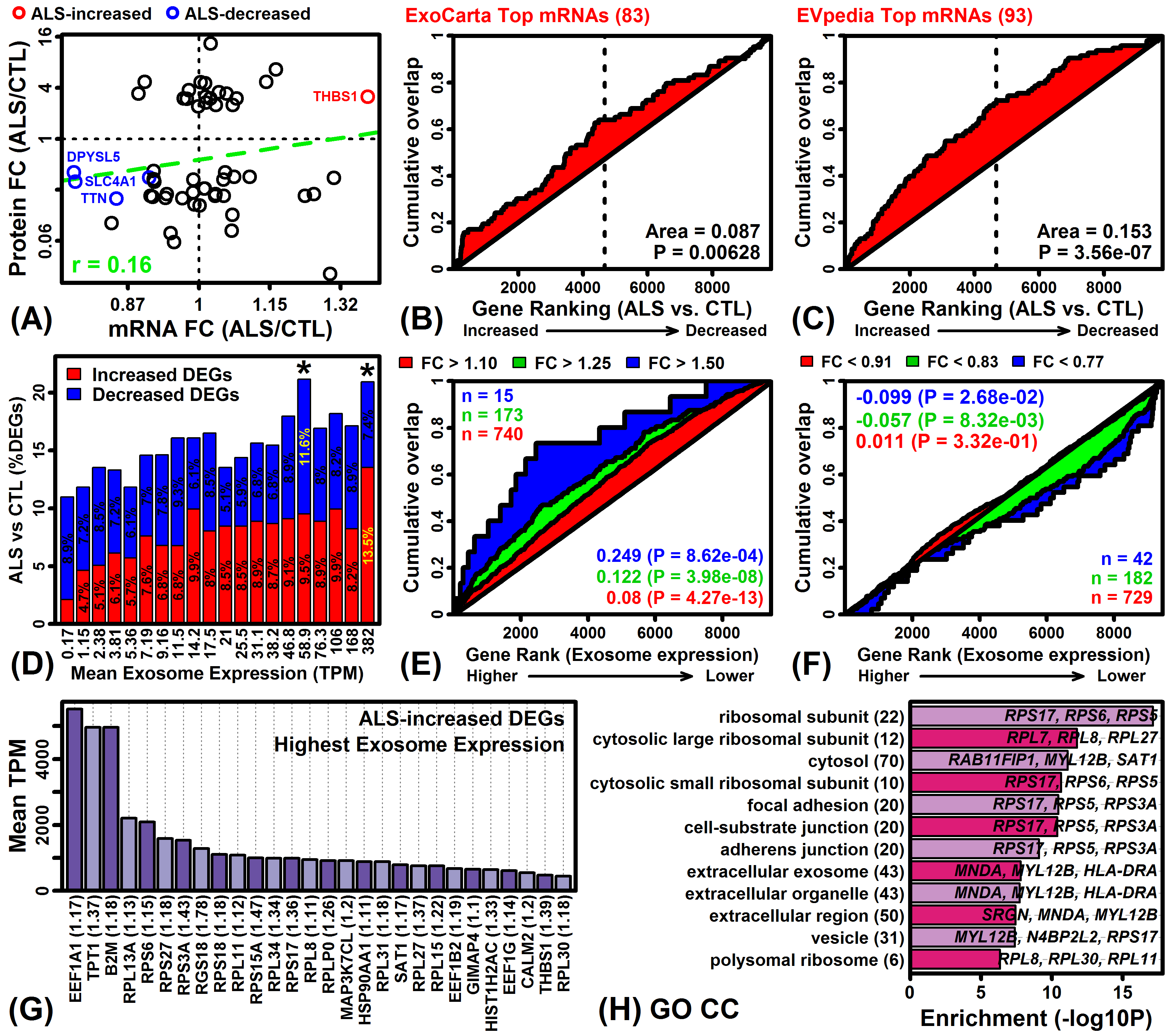

Supplement: Supplementary file 16 — Additional file 16. ALS DEG exosome expression. (A) Comparison of blood mRNA and exosome protein changes. FC estimates from the current study (ALS/CTL) were compared to those estimated in protein exosomes by Tomlinson et al. [65]. Red symbols denote mRNAs and proteins with ALS-increased expression in both studies (mRNA: FC > 1.10, FDR < 0.10; protein: FC > 2.0, P < 0.05) and blue symbols denote mRNAs and proteins with ALS-decreased expression in both studies (mRNA: FC < 0.909, FDR < 0.10; protein: FC < 0.50, P < 0.05). The spearman rank correlation is shown (lower left) with robust regression fit (dotted green line). (B) ExoCarta exosome-associated mRNA GSEA analysis. (C) EVpedia exosome-associated mRNA GSEA analysis. In (B) and (C), genes are ranked based upon their expression change in ALS vs. CTL patients (horizontal axis) and cumulative overlap with exosome-associated mRNAs is shown (vertical axis) (p-values: Wilcoxon rank sum test). (D) Percentage of ALS DEGs (vertical axis) in groups of genes stratified according to average blood exosome expression (horizontal axis) (n = 32 subjects; yellow font or black asterisk: P < 0.05, Fisher’s exact test; GSE100206). (E) ALS-increased DEG GSEA analysis. (F) ALS-decreased DEG GSEA analysis. In (E) and (F), genes were ranked based upon their average expression in blood exosomes (n = 32 subjects; GSE100206) (horizontal axis), and cumulative ALS DEG abundance is shown (vertical axis). The analysis was repeated with DEGs selected at varying FC thresholds (see legend; top margin) and large above-diagonal (positive) or below-diagonal (negative) indicate bias towards high and low exosome expression, respectively (p-values: Wilcoxon rank sum test). (G) ALS-increased DEGs with highest mean exosome expression. (H) GO CC terms. Enrichment was evaluated with respect to the 100 ALS-increased DEGs with highest mean exosome expression (GSE100206). The 12 most significantly over-represented annotations are listed (conditional hypergeomet [file 12967_2019_1909_MOESM16_ESM.tif]

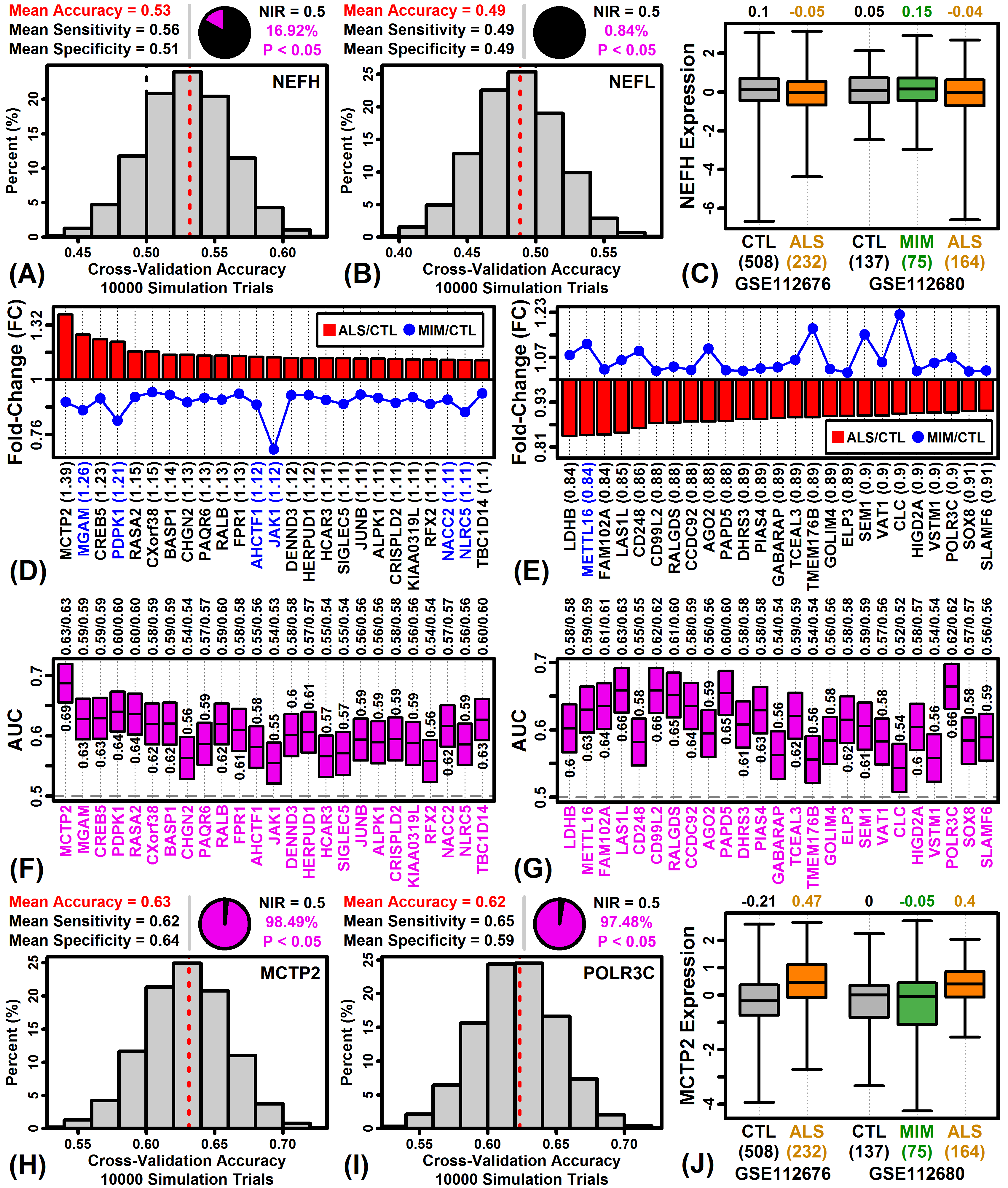

Supplement: Supplementary file 17 — Additional file 17. Single gene biomarkers for ALS diagnosis. (A, B) Cross-validation analysis of NEFH and NEFL prediction accuracy (10,000 simulations; logistic regression; training set: 296 ALS patients vs. 296 CTL/MIM subjects; testing set: 100 ALS patients vs. 100 CTL/MIM subjects). Histograms show the accuracy obtained across cross-validation trials. The proportion of trials in which accuracy was significantly greater than the non-information rate (NIR) of 50% is indicated (upper-right). (C) NEFH expression. Boxes outline the middle 50% of Z-score normalized expression values (whiskers: 10th to 90th percentiles). (D) ALS-increased DEGs most strongly decreased in MIM patients. Genes are ranked based upon the ALS/CTL FC estimate (parentheses, bottom margin; blue font: significantly decreased in MIM patients, FDR < 0.10, FC < 0.91). (E) ALS-decreased DEGs most strongly increased in MIM patients. Genes are ranked based upon the ALS/CTL FC estimate (parentheses, bottom margin). (F, G) AUC estimates. Boxes outline AUC 95% confidence intervals (middle bar: AUC point estimate; magenta font: 95% lower confidence limit > 0.50; upper margin: sensitivity/specificity). (H, I) Cross-validation analysis of MCTP2 and POLR3C prediction accuracy (as above). (J) MCTP2 expression (as above). [file 12967_2019_1909_MOESM17_ESM.tif]

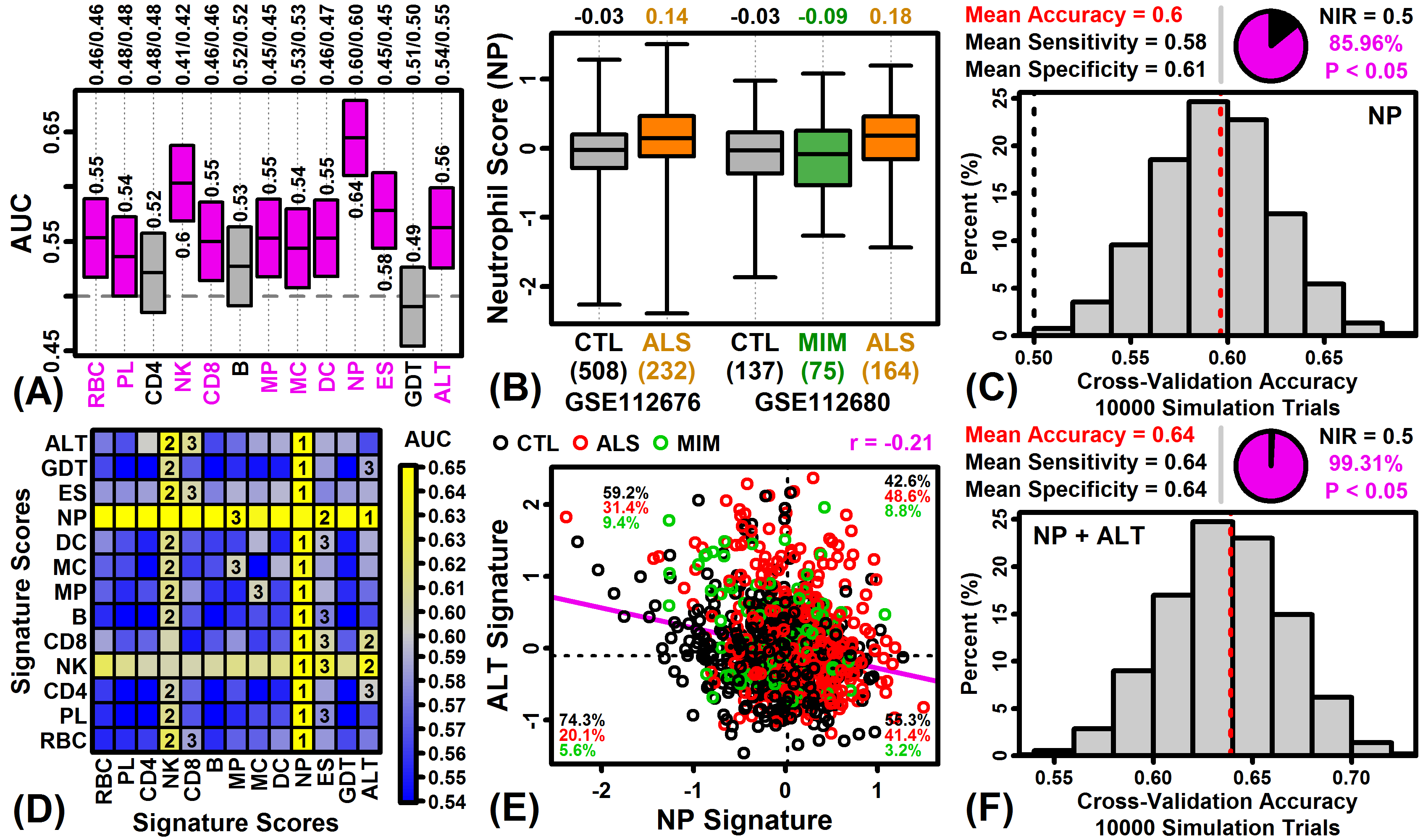

Supplement: Supplementary file 18 — Additional file 18. Gene signature biomarkers for ALS diagnosis. (A) AUC estimates. Boxes outline AUC 95% confidence intervals (middle bar: AUC point estimate; magenta font: 95% lower confidence limit > 0.50; upper margin: sensitivity/specificity). (B) Neutrophil signature scores. Boxes outline the middle 50% of scores in each group (whiskers: 10th to 90th percentiles). (C) Cross-validation analysis of NP signature prediction accuracy (10,000 simulations; logistic regression; training set: 296 ALS patients vs. 296 CTL/MIM subjects; testing set: 100 ALS patients vs. 100 CTL/MIM subjects). Histograms show the accuracy obtained across cross-validation trials. The proportion of trials in which accuracy was significantly greater than the non-information rate (NIR) of 50% is indicated (i.e., McNemar’s test; upper-right). (D) AUC estimates (logistic regression bivariate models). The heatmap shows AUC estimates for each bivariate combination (diagonal: univariate model AUCs). The 3 highest AUC estimates for each row are numbered (1 = highest AUC). (E) NP vs. ALT signature scatterplot. Dotted lines denote the median NP and ALS values and the percentage of ALS, MIM and CTL patients in each quadrant is indicated (magenta line: least squares regression estimate). (F) Cross-validation analysis of NP + ALT signature prediction accuracy (as above). [file 12967_2019_1909_MOESM18_ESM.tif]

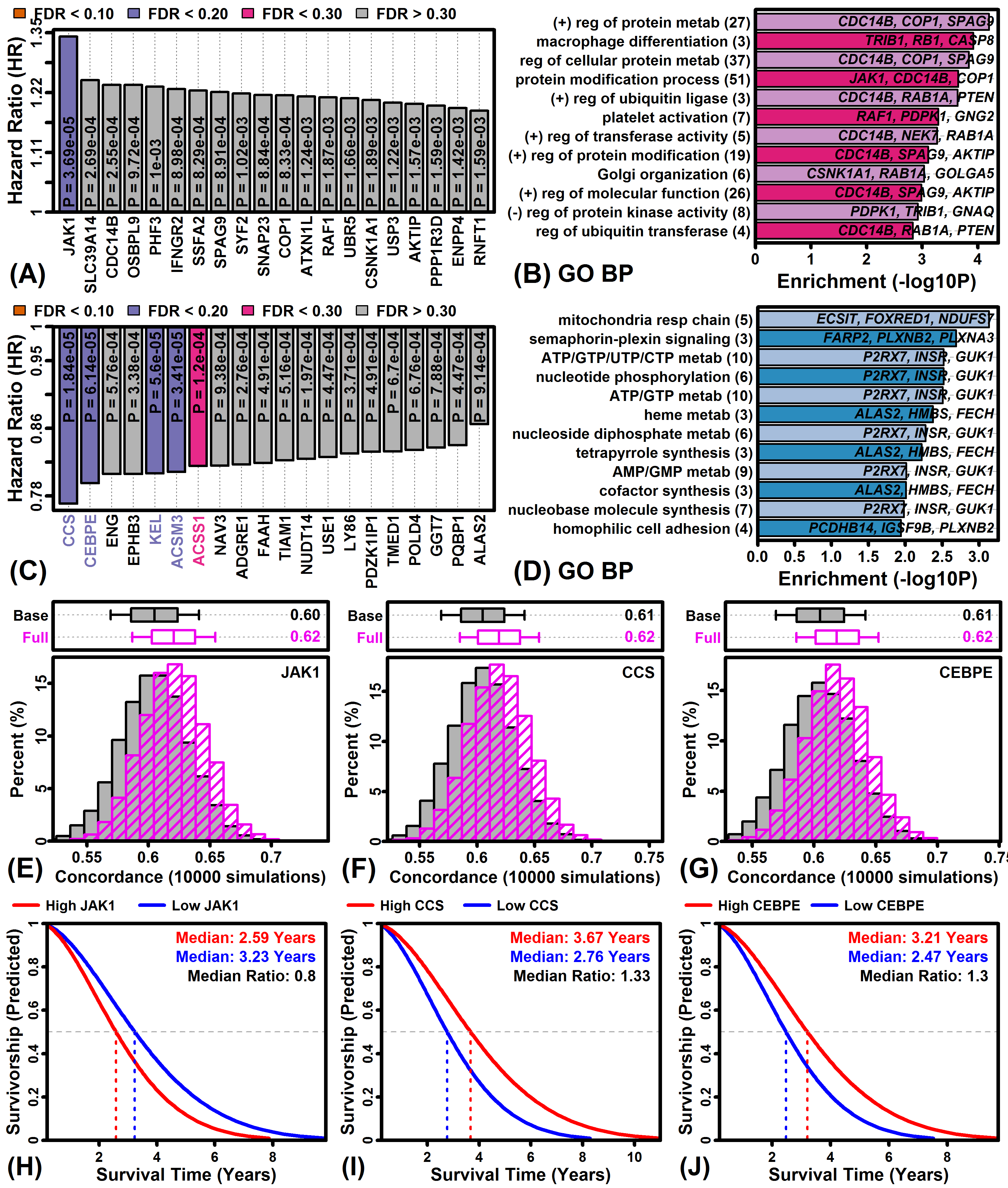

Supplement: Supplementary file 20 — Additional file 20. Genes with survival-associated expression. (A, C) Genes with expression (A) negatively associated with survival (HR > 1.00) or (C) positively associated with survival (HR < 1.00). Hazard ratios were estimated using Cox PH models (covariates: age, sex, site of onset, and cohort; n = 396 patients). Top-ranked genes were selected from among 11,480 protein-coding genes with detectable expression in at least 20% of ALS patients (> 80/396). (B, D) Gene Ontology Biological Process terms. Enrichment was evaluated with respect to survival-associated genes (P < 0.01; B: HR > 0; C: HR < 0). The 12 most significantly over-represented annotations are listed (conditional hypergeometric test). The number of genes associated with each annotation is listed (parentheses) with exemplar survival-associated genes. (E–G) Cross-validation analysis of survival prediction accuracy (10,000 simulations; Cox PH model; training set: 296 ALS patients; testing set: 100 ALS patients). Concordance index distributions are shown for the base model (clinical covariates only) and full model (clinical covariates + expression of the indicated gene). Boxes (top) outline the middle 50% of outcomes (middle line: median; whiskers: 10th to 90th percentile). (H–J) Predicted survivorship with low (20th percentile) and high (80th percentile) expression of (H) SPAG9, (I) KEL and (J) CCS. Median survivorship for each group is shown (dashed lines) with the median ratio between groups (upper-right). [file 12967_2019_1909_MOESM20_ESM.tif]

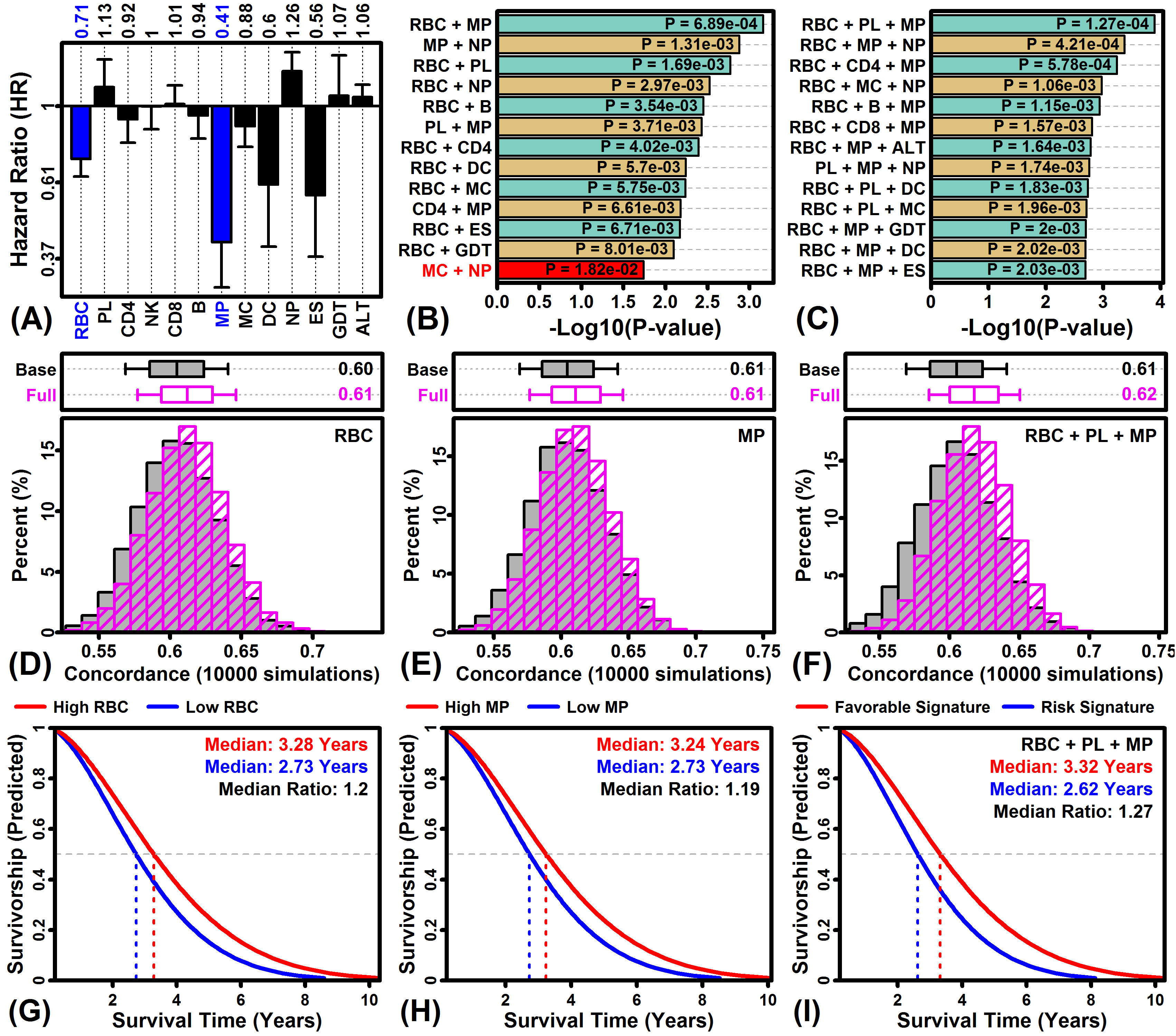

Supplement: Supplementary file 21 — Additional file 21. Cell type and altitude signature scores association with survival. (A) Cell type signature scores and hazard ratios (HRs) (blue font: P < 0.05; n = 396 patients; covariates: age, sex, site of onset, and cohort). (B) Top 2-way score combinations. The top 12 score combinations are listed. The MC + NP combination is shown for comparison (red bar). (C) Top 3-way score combinations. In (B) and (C), p-values were calculated by comparing full models (cell type scores + covariates) to reduced models (covariates only) using likelihood ratio tests. (D–F) Cross validation evaluation of immune cell score prediction accuracy (10,000 simulations; Cox PH model; training set: 296 ALS patients; testing set: 100 ALS patients). Concordance index distributions are shown for the base model (covariates only) and full model (covariates + immune cell scores). Boxes (top) outline the middle 50% of outcomes (middle line: median; whiskers: 10th to 90th percentile). (G, H) Predicted survivorship with low (20th percentile) and high (80th percentile) signature scores. (I) Predicted survivorship with favorable and risk-associated expression signatures (RBC + PL + MP + covariates; favorable signature: patient with 80th percentile predicted survival time; risk signature: patient with 20th percentile predicted survival time). In (G)–(I), median survivorship for each group is shown (dashed lines) with the median ratio between groups (upper-right). [file 12967_2019_1909_MOESM21_ESM.tif]

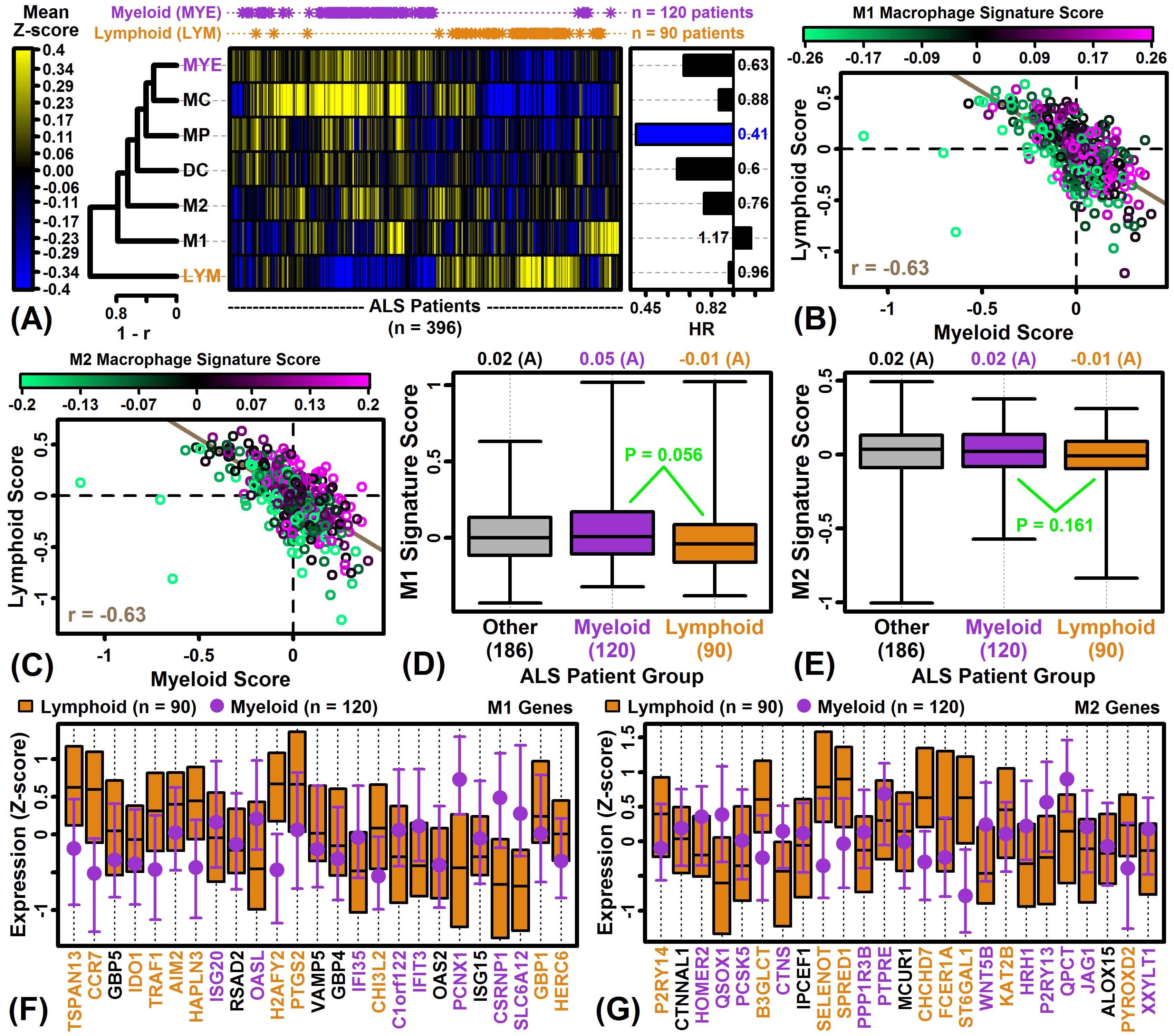

Supplement: Supplementary file 22 — Additional file 22. M1 and M2 macrophage signature scores in myeloid and lymphoid patient subgroups. (A) Monocyte-lineage signature scores and hazard ratios (HRs). Scores were calculated by averaging Z-score normalized expression of the 150 genes most specifically expressed in each cell type. Composite myeloid (MYE) and lymphoid (LYM) scores are also shown (MYE: DC, MP, MC, NP, PL, RBC and ES; LYM: CD8, CD4, GDT, NK and B). Heatmap rows and columns are clustered (rows: 1–correlation; columns: Euclidean distance; right: hazard ratios; covariates: age, sex, site of onset, and cohort; n = 396 patients). (B) M1 macrophage signature scores. (C) M2 macrophage signature scores. In (B) and (C), the 396 patients are plotted with respect to myeloid and lymphoid signature scores and colors denote M1 or M2 scores for each patient. (D) M1 score group comparison. (E) M2 score group comparison. In (D) and (E), boxes outline the middle 50% of patients in each group (middle line: median) and whiskers span the 10th to 90th percentiles. The group median is shown (top margin) with shared letters (parentheses) indicating no significant difference among groups (P > 0.05; Fisher’s Least Significant Difference). The p-value (green font) was obtained by comparing scores from the myeloid and lymphoid groups (Wilcoxon rank sum test). (F) Genes with elevated expression in M1 macrophages (compared to non-polarized macrophages; GSE5099). (G) Genes with elevated expression in M2 macrophages (compared to non-polarized macrophages; GSE5099). In (F) and (G), boxes outline the middle 50% of expression values for lymphoid group patients, and error bars outline the middle 50% of values for myeloid group patients. Genes with a significant difference in expression between groups (FDR < 0.10) are shown in colored font (bottom margin). [file 12967_2019_1909_MOESM22_ESM.tif]
