# Supplementary material for: ALS blood expression profiling identifies new biomarkers, patient subgroups, and evidence for neutrophilia and hypoxia
Source: J Transl Med. 2019 May 22;17:170. doi: 10.1186/s12967-019-1909-0 (PMC6530130; doi:10.1186/s12967-019-1909-0)
Supplement: Supplementary file 19 — Additional file 19. Published sensitivity and specificity estimates for ALS sample classification. The table lists the first author of each study and publication year, PubMed identifier (PMID), biofluid source, samples sizes (ALS and CTL groups), type of CTL group, biomarker or rule applied for classification, reported sensitivity (Sens) and specificity (Spec). For the “CTL type” column, values are healthy controls (HC), diseased control (DC), or the combination of healthy and diseased controls (HC + DC). [file 12967_2019_1909_MOESM19_ESM.pdf]

Additional File 19. Published sensitivity and specificity estimates for ALS sample classification. The table lists the first author of each study and publication year, PubMed identifier (PMID), biofluid source, samples sizes (ALS and CTL groups), type of CTL group, biomarker or rule applied for classification, reported sensitivity (Sens) and specificity (Spec). For the "CTL type" column, values are healthy controls (HC), diseased control (DC), or the combination of healthy and diseased controls (HC+DC).

Study	PMID	Fluid	*n* (ALS)	*n* (CTL)	CTL Type	Biomarker	Sens	Spec
Ranganathan 2005	28799854	CSF	10	10	DC	Transthyretin	70.0	60.0
Ranganathan 2005	28799854	CSF	5	7	HC+DC	7B2CT + TTR	80.0	71.0
Ranganathan 2005	28799854	CSF	10	10	DC	10 protein panel	80.0	60.0
Ranganathan 2005	28799854	CSF	5	7	HC+DC	19 protein panel	80.0	100.0
Brettschneider 2006	16567701	CSF	69	33	HC	NFH	71.0	88.0
Brettschneider 2006	16567701	CSF	69	33	HC	Tau	52.0	75.0
Brettschneider 2006	16567701	CSF	69	73	DC	NFH	89.0	92.0
Brettschneider 2006	16567701	CSF	69	73	DC	Tau	81.0	78.0
Pasinetti 2006	16481598	CSF	36	21	HC	4.8 kDa protein	48.0	86.0
Pasinetti 2006	16481598	CSF	36	21	HC	6.7 kDa protein	91.0	86.0
Pasinetti 2006	16481598	CSF	36	21	HC	13.4 kDa protein	91.0	50.0
Pasinetti 2006	16481598	CSF	36	21	HC	3 protein model	91.0	97.0
Mitchell 2009	18987350	CSF	41	33	DC	5 protein panel	87.5	91.2
Reijn 2009	19296046	CSF	32	26	DC	pNFH > 502 ng/L	72.0	80.0
Reijn 2009	19296046	CSF	32	26	DC	NFL > 22.6 ng/L	75.0	79.0
Ryberg 2010	20583124	CSF	100	41	HC	CRP > 9 mg/ml	51.0	85.0
Ryberg 2010	20583124	CSF	100	41	HC	Transthyretin	63.0	70.0
Ryberg 2010	20583124	CSF	100	41	HC	41 Biomarker Panel	63.0	94.0
Ryberg 2010	20583124	CSF	100	141	HC+DC	CRP	65.0	60.0
Ryberg 2010	20583124	CSF	100	141	HC+DC	Cystatin C	60.0	71.0
Ryberg 2010	20583124	CSF	100	141	HC+DC	Transthyretin	63.0	47.0
Ganesalingam 2011	21418221	CSF	26	31	HC+DC	pNFH	95.0	93.0
Ganesalingam 2011	21418221	CSF	26	31	HC+DC	pNFH/C3 ratio	96.0	90.0
Ganesalingam 2011	21418221	CSF	45	61	HC+DC	pNFH > 0.635 ng/mL	84.4	93.5
Ganesalingam 2011	21418221	CSF	45	61	HC+DC	pNFH/C3 > 0.000125	91.1	88.7
Ganesalingam 2011	21418221	CSF	45	61	HC+DC	pNFH/C3 > 0.21	77.8	95.2
Ganesalingam 2011	21418221	CSF	45	61	HC+DC	pNFH/CRP > 0.3755	61.4	87.1

Ganesalingam 2011	21418221	CSF	45	61	HC+DC	pNFH > 0.635 + pNFH/C3 > 0.000125	84.4	95.2
Ganesalingam 2011	21418221	CSF	45	61	HC+DC	pNFH/C3 > 0.00021 + pNFH/CRP > 0.3755	52.2	100.0
Ganesalingam 2011	21418221	CSF	45	61	HC+DC	pNFH/C3 > 0.00021 or pNFH/CRP > 0.3755	86.4	83.9
Ganesalingam 2011	21418221	CSF	45	61	HC+DC	pNFH > 0.635 + pNFH/C3 > 0.000125 + pNFH/CRP > 0.3755	59.1	100.0
Tortelli 2012	22680408	CSF	37	46	DC+DC	NFL > 1981 ng/l	78.4	72.5
Conraux 2013	24224000	Blood	10	10	HC	C18 SVM Peptide Model	97.9	99.7
Conraux 2013	24224000	Blood	10	10	HC	C8 SVM Peptide Model	87.8	96.9
Gaiottino 2013	24073237	Blood	46	67	HC	NFL > 26.6 pg/ml	91.3	91.0
Hwang 2013	23639787	Blood	61	120	HC+DC	HMGB1 autoAb > 0.81 μg/mL	64.0	93.0
Lawton 2014	24984169	Blood	172	50	HC	Random Forest w/ 367 metabolites	76.0	78.0
Lawton 2014	24984169	Blood	172	73	DC	SVM w/ 367 metabolites	62.0	81.0
Lawton 2014	24984169	Blood	172	73	DC	Lasso w/ 367 metabolites	65.0	81.0
Lawton 2014	24984169	Blood	172	73	DC	Random Forest w/ 367 metabolites	62.0	66.0
Lawton 2014	24984169	Blood	172	73	DC	LASSO w/ 32 metabolites	58.0	90.0
Lawton 2014	24984169	Blood	172	73	DC	SVM w/ 32 metabolites	49.0	90.0
Shepheard 2014	24475283	Urine	28	12	HC	p75NTR	93.0	100.0
Shepheard 2014	24475283	Urine	28	19	DC	p75NTR	93.0	79.0
Gonçalves 2015	25261856	CSF	29	19	DC	pNFH > 385 pg/ml	82.8	64.7
Gonçalves 2015	25261856	CSF	29	19	DC	N-glycans > 5.96%	41.4	82.4
Lu 2015	25934855	CSF	38	20	HC	NFL > 1781 pg/ml	97.0	95.0
Lu 2015	25934855	Blood	64	36	HC	NFL > 36 pg/ml	89.0	75.0
Lu 2015	25934855	Blood	103	42	HC	NFL > 36.2 pg/ml	90.0	71.0
Chen 2016	27634542	CSF	40	40	DC	pNFH > 437 ng/L	97.3	83.8
Chen 2016	27634542	CSF	40	40	DC	Chitotriosidase > 1593.779 ng/L	83.8	81.1

Chen 2016	27634542	CSF	40	40	DC	pNFH + Chitotriosidase	83.8	91.9
Oeckl 2016	27415180	CSF	75	75	HC+DC	pNFH >568.5 pg/mL	78.7	93.3
Oeckl 2016	27415180	CSF	75	75	HC+DC	NFL > 1431 pg/mL	79.0	86.4
Steinacker 2016	26296871	CSF	253	85	DC	NFL > 2200 pg/mL	77.0	88.0
Steinacker 2016	26296871	CSF	253	85	DC	pNFH > 560 pg/mL	83.0	80.0
Sheinerman 2017	29121998	Blood	50	50	HC	miR-206 + miR-338-3p	68.0	74.0
Sheinerman 2017	29121998	Blood	50	50	HC	miR-9* + miR-129-3p	65.0	67.0
Sheinerman 2017	29121998	Blood	50	50	HC	miR-335-5p + miR-338-3p	62.0	74.0
Sheinerman 2017	29121998	Blood	50	50	HC	miRNA classifier	84.0	82.0
Sheinerman 2017	29121998	Blood	50	50	DC	miR-31 + miR-206	78.0	78.0
Sheinerman 2017	29121998	Blood	50	50	DC	miR-125b + miR-335-5p	62.0	72.0
Sheinerman 2017	29121998	Blood	50	50	DC	miR-107 + miR-491-5p	74.0	70.0
Sheinerman 2017	29121998	Blood	50	50	DC	miRNA classifier	92.0	94.0
Sheinerman 2017	29121998	Blood	50	50	DC	miR-9* + miR-206	78.0	78.0
Sheinerman 2017	29121998	Blood	50	50	DC	miR-155 + miR-206	80.0	74.0
Sheinerman 2017	29121998	Blood	50	50	DC	miR-7 + miR-206	80.0	76.0
Sheinerman 2017	29121998	Blood	50	50	DC	miRNA classifier	82.0	80.0
Li 2018	30210445	CSF	53	32	DC	pNFH > 1104 pg/mL	100.0	68.8
Li 2018	30210445	CSF	53	32	DC	NFL > 1139 pg/mL	96.2	56.3
Salter 2018	29941342	Blood	40	34	HC	EpiSwitch Signature	83.3	76.9
Salter 2018	29941342	Blood	8	8	HC	EpiSwitch Signature	87.5	75.0
