# Supplementary material for: ALS blood expression profiling identifies new biomarkers, patient subgroups, and evidence for neutrophilia and hypoxia
Source: J Transl Med. 2019 May 22;17:170. doi: 10.1186/s12967-019-1909-0 (PMC6530130; doi:10.1186/s12967-019-1909-0)
Supplement: Supplementary file 23 — Additional file 23. 61 gene signature for prediction of ALS patient survival. The table lists the 61 genes incorporated into the final Cox PH regression model developed to predict ALS patient survival. Columns 2 and 3 provide hazard ratio (HR) estimates from unigenic Cox PH models, which were fit with five predictor variables, including expression of the gene listed in column 1 with four covariates (age, sex, site of onset, cohort). The second column gives the HR estimate with 95% confidence limits (HR > 1: increased expression = worse survival; HR < 1: increased expression = improved survival). Columns 4 and 5 provide results from likelihood ratio tests (LRT) comparing goodness of fit between full models (61 predictor genes + 4 covariates) and reduced models (60 predictor genes + 4 covariates, with removal of the gene listed in column 1). The χ2 statistic obtained from each LRT is given (column 4) with p-value obtained from the χ2 null distribution with 1 degree of freedom (column 5). Genes for which expression contributes most to goodness of fit have larger χ2 statistics (and lower p-values). [file 12967_2019_1909_MOESM23_ESM.pdf]

Additional File 23. 61 gene signature for prediction of ALS patient survival. The table lists the 61 genes incorporated into the final Cox PH regression model developed to predict ALS patient survival. Columns 2 and 3 provide hazard ratio (HR) estimates from unigenic Cox PH models, which were fit with five predictor variables, including expression of the gene listed in column 1 with four covariates (age, sex, site of onset, cohort). The second column gives the HR estimate with 95% confidence limits (HR > 1: increased expression = worse survival; HR < 1: increased expression = improved survival). Columns 4 and 5 provide results from likelihood ratio tests (LRT) comparing goodness of fit between full models (61 predictor genes + 4 covariates) and reduced models (60 predictor genes + 4 covariates, with removal of the gene listed in column 1). The χ^2 statistic obtained from each LRT is given (column 4) with p-value obtained from the χ^2 null distribution with 1 degree of freedom (column 5). Genes for which expression contributes most to goodness of fit have larger χ^2 statistics (and lower p-values).

Symbol	Unigenic Cox PH Model		Multigenic Cox PH Model	
	HR	P-Value	χ^2 Statistic	P-value
SLC39A14	1.25 (1.11, 1.4)	0.000269	13.72	0.00021
CDC14B	1.24 (1.1, 1.39)	0.000255	8.96	0.0028
OSBPL9	1.24 (1.09, 1.4)	0.000972	10.35	0.0013
IFNGR2	1.23 (1.09, 1.39)	0.000898	7.5	0.0062
ATXN1L	1.21 (1.08, 1.36)	0.00124	2.24	0.13
UBR5	1.21 (1.07, 1.36)	0.00166	11.81	0.00059
USP3	1.2 (1.07, 1.34)	0.00122	4.4	0.036
RAB1A	1.2 (1.06, 1.36)	0.00396	6.9	0.0086
RNF38	1.19 (1.06, 1.34)	0.0045	10.98	0.00092
ELAC1	1.19 (1.06, 1.34)	0.00334	26.6	2.5E-07
RNFT1	1.18 (1.07, 1.32)	0.00159	9.53	0.002
JPH3	1.18 (1.04, 1.33)	0.00825	10.06	0.0015
TM6SF1	1.18 (1.05, 1.32)	0.00464	7.77	0.0053
ITPR2	1.18 (1.05, 1.32)	0.00552	5.75	0.016
ME2	1.18 (1.04, 1.33)	0.00959	5.7	0.017
SLC40A1	1.17 (1.03, 1.32)	0.0124	9.75	0.0018
YWHAH	1.17 (1.05, 1.3)	0.00505	8.46	0.0036
C16orf86	1.16 (1.04, 1.3)	0.00824	8.72	0.0031
KIDINS220	1.16 (1.04, 1.29)	0.00777	10.68	0.0011

CLEC7A	1.15 (1.04, 1.27)	0.00498	3.54	0.06
MTFMT	1.15 (1.02, 1.3)	0.0277	3.78	0.052
RAB21	1.15 (1.03, 1.28)	0.0121	20.42	6.2E-06
FZD3	1.14 (1.02, 1.29)	0.0237	7.19	0.0074
MAN2A2	1.14 (1.03, 1.27)	0.0158	3.41	0.065
CLEC4D	1.14 (1.02, 1.26)	0.0172	5.95	0.015
PRKCB	1.13 (1.02, 1.26)	0.0168	8.05	0.0046
NUP58	1.13 (1.01, 1.26)	0.0349	18.85	0.000014
FBXO4	1.13 (1.01, 1.25)	0.0259	14.32	0.00015
SEC62	1.13 (1.01, 1.25)	0.0293	5.84	0.016
TRIM22	1.11 (1.003, 1.24)	0.043	6.51	0.011
IGSF11	0.9 (0.8, 0.997)	0.043	22.94	1.7E-06
RGS17	0.89 (0.8, 0.996)	0.0423	2.15	0.14
SERPINH1	0.89 (0.8, 0.99)	0.0269	7.29	0.0069
C9orf78	0.89 (0.8, 0.99)	0.027	6.16	0.013
TRPM7	0.88 (0.8, 0.98)	0.0156	15.75	0.000072
SLC38A10	0.88 (0.79, 0.98)	0.0216	14.82	0.00012
ZNF598	0.88 (0.79, 0.98)	0.0202	4.91	0.027
DCAF4L1	0.88 (0.78, 0.99)	0.0303	12.6	0.00039
ZNF429	0.88 (0.78, 0.98)	0.0265	35.59	2.4E-09
PHLDA2	0.88 (0.79, 0.97)	0.0135	9.29	0.0023
C12orf10	0.87 (0.79, 0.96)	0.00583	24.89	6.1E-07
MIS18A	0.87 (0.78, 0.97)	0.0124	25.47	4.5E-07
TASP1	0.87 (0.78, 0.98)	0.0198	14.11	0.00017
CCDC51	0.87 (0.77, 0.97)	0.0123	4.44	0.035
LMNA	0.86 (0.78, 0.95)	0.00336	21.68	3.2E-06
MTMR11	0.86 (0.78, 0.96)	0.00543	7.3	0.0069
NME4	0.86 (0.77, 0.95)	0.00374	3.46	0.063
RNASE2	0.86 (0.77, 0.95)	0.00307	7.44	0.0064

VRK3	0.85 (0.76, 0.96)	0.00691	5.11	0.024
CDIP1	0.85 (0.76, 0.96)	0.00674	4.46	0.035
PCDHB14	0.85 (0.77, 0.94)	0.00229	13.15	0.00029
APCDD1	0.85 (0.77, 0.94)	0.00127	13.04	0.0003
WDR6	0.85 (0.76, 0.95)	0.00466	14.8	0.00012
ACADS	0.85 (0.76, 0.94)	0.00234	15.72	0.000073
ITLN1	0.85 (0.76, 0.94)	0.00165	12.72	0.00036
LAMTOR2	0.84 (0.76, 0.94)	0.00176	4.77	0.029
TMEM147	0.84 (0.76, 0.94)	0.00152	4.47	0.034
C19orf44	0.84 (0.76, 0.93)	0.00126	5.96	0.015
GGT7	0.84 (0.75, 0.93)	0.000788	7.28	0.007
CEBPE	0.79 (0.71, 0.89)	6.14E-05	6.7	0.0096
CCS	0.77 (0.68, 0.87)	1.84E-05	12.85	0.00034
